# Supplementary material for: Looking at the fringes of MedTech innovation: a mapping review of horizon scanning and foresight methods
Source: BMJ Open. 2023 Sep 14;13(9):e073730. doi: 10.1136/bmjopen-2023-073730 (PMC10503360; doi:10.1136/bmjopen-2023-073730)
Supplement: Supplementary data [file bmjopen-2023-073730supp002.pdf]

Appendix B

Figure B.1. MEDLINE search strategy

1	Forecasting/
2	(horizon scan or horizon scanning).ti,ab,kf,tw.
3	(environmental scan or environmental scanning).ti,ab,kf,tw.
4	(foresight or forecasting).ti,ab,kf,tw.
5	("Early awareness" and "alert systems").ti,ab,kf,tw.
6	(readiness adj3 preparedness).ti,ab,kf,tw.
7	(monitor* adj6 ("health technolog*" or "medical technolog*")).ti,ab,kf,tw.
8	("road map" or roadmap).ti,ab,kf,tw.
9	patent analys*s.ti,ab,kf,tw.
10	bibliometric analys*s.ti,ab,kf,tw.
11	trend* analys*s.ti,ab,kf,tw.
12	backcasting.ti,ab,kf,tw.
13	delphi.ti,ab,kf,tw.
14	*delphi technique/
15	or/1-14
16	biomedical technology/
17	exp technology assessment, biomedical/
18	((new or innovat* or emerg* or early) adj6 ("health technolog*" or "medical technolog*")).ti,ab.
19	or/16-18
20	15 and 19

Databases searched

Medline (OVID)

Database: Ovid MEDLINE(R) and Epub Ahead of Print, In-Process, In-Data-Review & Other Non-Indexed Citations, Daily and Versions(R) <1946 to March 25, 2021>

Date: 26/03/2021

Search results: 1003

Search Strategy:

----

1 Forecasting/ (87084)

2 (horizon scan or horizon scanning).ti,ab,kf,tw. (247)

3 (environmental scan or environmental scanning).ti,ab,kf,tw. (1780)

4 (foresight or forecasting).ti,ab,kf,tw. (10290)

5 ("Early awareness" and "alert systems").ti,ab,kf,tw. (9)

6 (readiness adj3 preparedness).ti,ab,kf,tw. (203)

7 (monitor* adj6 ("health technolog*" or "medical technolog*")).ti,ab,kf,tw. (93)

8 ("road map" or roadmap).ti,ab,kf,tw. (7122)

9 patent analys*s.ti,ab,kf,tw. (75)

10 bibliometric analys*s.ti,ab,kf,tw. (2302)

11 trend* analys*s.ti,ab,kf,tw. (5160)

12 backcasting.ti,ab,kf,tw. (29)

13 delphi.ti,ab,kf,tw. (12256)

14 *delphi technique/ (1233)

15 or/1-14 (123740)

16 biomedical technology/ (6719)

17 exp technology assessment, biomedical/ (11322)

18 ((new or innovat* or emerg* or early) adj6 ("health technolog*" or "medical technolog*")).ti,ab. (1640)

19 or/16-18 (18729)

20 15 and 19 (1003)

2. Embase

Database: Embase <1974 to 2021 March 25>

Date: 26/03/2021

Search results: 1295

Search Strategy:

----

1 forecasting/ (46958)

2 (horizon scan or horizon scanning).ti,ab,kw. (341)

3 (environmental scan or environmental scanning).ti,ab,kw. (2175)

4 (foresight or forecasting).ti,ab,kw. (12472)

5 (readiness adj3 preparedness).ti,ab,kw. (218)

6 (monitor* adj6 ("health technolog*" or "medical technolog*")).ti,ab,kw. (142)

7 ("road map" or roadmap).ti,ab,kw. (8918)

8 patent analys*s.ti,ab,kw. (181)

9 bibliometric analys*s.ti,ab,kw. (2705)

10 trend* analys*s.ti,ab,kw. (7382)

11 backcasting.ti,ab,kw. (31)

12 delphi.ti,ab,kw. (16900)

13 *delphi study/ (2074)

14 *bibliometrics/ (3371)

15 *trend study/ (865)

16 or/1-15 (95349)

17 medical technology/ (34204)

18 biomedical technology assessment/ (15008)

19 ((new or innovat* or emerg* or early) adj6 ("health technolog*" or "medical technolog*")).ti,ab. (2248)

20 or/17-19 (49615)

21 16 and 20 (1295)

3. IEEE Xplore

Database URL: https://ieeexplore-ieee-org.libproxy.ncl.ac.uk/search

Search Date: 05032021

Search strategy: database does not allow to combine search strings or save searches. Searched string by string and assessed relevance of results on screen by title and abstract. Those results judged relevant as first screening have been downloaded for further assessment.

No time limit, No language limits

Showing 1-4 of 4 for ("Document Title":"horizon scan" OR "Abstract":"horizon scan" OR "Index Terms":"horizon scan") – search retrieved no relevant results

Showing 1-25 of 437 for ("Publication Title":"foresight" OR "Abstract":"foresight" OR "Index Terms":"foresight") – all 437 results downloaded for further assessment

Conferences (352)

Journals (50)

Magazines (21)

Early Access Articles (12)

Books (1)

Courses (1)

Showing 1-25 of 71 for (("Document Title":"forecast" OR "Abstract":"forecast" OR "Index Terms":"forecast") AND "Document Title":"health") – all 71 results downloaded for further assessment

Conferences (59)

Journals (11)

Magazines (1)

Showing 1-19 of 19 for (("Document Title":"roadmap" OR "Abstract":"roadmap" OR "Index Terms":"roadmap") AND "Document Title":"health") – search retrieved no relevant results

Showing 1-17 of 17 for (("Document Title":"patent" OR "Document Title":"bibliometric" OR "Document Title":"trend" OR "Document Title":"backcasting" OR "Document Title":"delphi") AND "Document Title":"health") – 1 reference downloaded for further assessment/all references downloaded for further assessment.

4. Compendex – Engineering Village (Elsevier)

Database URL: https://www.engineeringvillage.com/search/quick.url

Search date: 26/03/2020

Search strategy:

((((((environmental scanning NEAR/6 health) OR (horizon scanning NEAR/6 health) OR (Forecasting NEAR/6 health) OR (Foresight NEAR/06 health) OR (Roadmap NEAR/06 health) OR (patent analys*s NEAR/06 health) OR (bibliometric analys*s NEAR/06 health) OR (trend analys*s NEAR/06 health) OR (backcasting NEAR/06 health) OR (delphi NEAR/06 health)) WN KY)) NOT ({cp} WN DT)) NOT (({ch} OR {bk} OR {er}) WN DT))

Removed conference proceedings, books and book chapters:

Retrieved results: 3,465

5. Scopus

Advanced search interface

Database URL: https://www.scopus.com/search/form.uri?display=advanced

Search date: 09/03/2021

Retrieved results: 2,396

Search strategy:

(TITLE-ABS-KEY ("horizon scanning") OR TITLE-ABS-KEY (forecasting) OR TITLE-ABS-KEY ("road map" OR roadmap) OR TITLE-ABS-KEY (patent AND anlys*s) OR TITLE-ABS-KEY (bibliometric AND analys*s) OR TITLE-ABS-KEY (trend AND analys*s) OR TITLE-ABS-KEY (backcasting) OR TITLE-ABS-KEY (delphi)) AND (TITLE-ABS-KEY ("medical technology") OR TITLE-ABS-KEY ("health technology"))

6. Web of Science CORE Collection

Advanced search interface

Database URL:
https://apps.webofknowledge.com/WOS_AdvancedSearch_input.do?product=WOS&search_mode=AdvancedSearch&SID=D5cLI4VsqgVb6Yc6hBB&locale=en_US

Date searched 09/03/2021

Retrieved results: 214 all downloaded for further assessment

Search strategy:

Set	Results	Save History / Create AlertOpen Saved History
# 12	214	#11 AND #8<br>*Indexes=SCI-EXPANDED, SSCI, A&HCI, CPCI-S, CPCI-SSH, ESCI Timespan=All years*
# 11	11,875	#10 OR #9<br>*Indexes=SCI-EXPANDED, SSCI, A&HCI, CPCI-S, CPCI-SSH, ESCI Timespan=All years*
# 10	7,049	TI=("health technology" OR "health technologies") OR AB=("health technology" OR "health technologies")<br>*Indexes=SCI-EXPANDED, SSCI, A&HCI, CPCI-S, CPCI-SSH, ESCI Timespan=All years*
# 9	4,942	TI=("medical technology" OR "medical technologies") OR AB=("medical technology" OR "medical technologies")<br>*Indexes=SCI-EXPANDED, SSCI, A&HCI, CPCI-S, CPCI-SSH, ESCI Timespan=All years*
# 8	236,408	#7 OR #6 OR #5 OR #4 OR #3 OR #2 OR #1<br>*Indexes=SCI-EXPANDED, SSCI, A&HCI, CPCI-S, CPCI-SSH, ESCI Timespan=All years*
# 7	19,579	TI=(delphi) OR AB=(delphi)<br>*Indexes=SCI-EXPANDED, SSCI, A&HCI, CPCI-S, CPCI-SSH, ESCI Timespan=All years*
# 6	345	TI=(backcasting) OR AB=(backcasting)<br>*Indexes=SCI-EXPANDED, SSCI, A&HCI, CPCI-S, CPCI-SSH, ESCI Timespan=All years*
# 5	10,036	TI=("trend analysis" OR "trend analyses") OR AB=("trend analysis" OR "trend analyses")<br>*Indexes=SCI-EXPANDED, SSCI, A&HCI, CPCI-S, CPCI-SSH, ESCI Timespan=All years*
# 4	5,608	TI=("bibliometric analysis" OR "bibliometric analyses") OR AB=("bibliometric analysis" OR "bibliometric analyses")<br>*Indexes=SCI-EXPANDED, SSCI, A&HCI, CPCI-S, CPCI-SSH, ESCI Timespan=All years*
# 3	749	TI=("patent analysis" OR "patent analyses") OR AB=("patent analysis" OR "patent analyses")<br>*Indexes=SCI-EXPANDED, SSCI, A&HCI, CPCI-S, CPCI-SSH, ESCI Timespan=All years*
# 2	21,200	TI=("road map" OR roadmap) OR AB=("road map" OR roadmap)<br>*Indexes=SCI-EXPANDED, SSCI, A&HCI, CPCI-S, CPCI-SSH, ESCI Timespan=All years*
# 1	180,250	TI=(horizon scanning OR forecasting) OR AB=(horizon scanning OR forecasting)

Indexes=SCI-EXPANDED, SSCI, A&HCI, CPCI-S, CPCI-SSH, ESCI Timespan=All years

7. International HTA Database

URL: https://database.inahta.org/search/advanced

Date searched 04/02/2022

Retrieved results: 334

Search strategy:

(forecast*)[Title] OR (horizon scan*)[Title] OR (environmental scan*)[Title] OR (foresight)[Title] OR ("early awareness" AND "alert systems")[Title] OR (roadmap* OR "road map*")[Title] OR ("bibliometric analys*")[Title] OR (backcasting)[Title] OR ("trend analys*")[Title] OR (delphi) AND ((new OR innovative OR early OR emerging))

Retrieved 334. Sifted on screen. 35 results downloaded for further assessment. Included in lit review: 0

Grey literature searches:

Platform: Google Chrome

Search strategy:

horizon scanning filetype:pdf

Date: 26/02/2021

Results: limited to 2020 onwards, checked the first 5 pages of results.

Included for further analysis:

https://www.pcori.org/sites/default/files/PCORI-Health-Care-COVID-19-Horizon-Scanning-System-Supplement-High-Impact-Report-September-2020.pdf

https://ec.europa.eu/info/sites/info/files/srip/2020/rec-19-003_srip_chap-15.pdf

https://www.mdpi.com/2071-1050/12/23/10194/pdf

https://htai.org/wp-content/uploads/2020/03/HTAi_APF-2019-Background-Paper.pdf

Database: CORE

Date searched 26/02/2021

Search strategy: “horizon scanning”

Retrieved results: 5,582

Hand searched the first 5 results pages.

Included for further assessment: 2

Blackburn, Steven, Cudd, Peter and Hawley, Mark (2010) A horizon scanning system for identifying new telehealth innovations. Global Telemedicine and eHealth Updates: Knowledge Resources, 3. pp. 326-330. ISSN 1998-5509 http://eprints.whiterose.ac.uk/11144/

Enhancing Horizon Scanning by utilizing pre-developed scenarios: analysis of current practice and specification of a process improvement to aid the identification of important 'weak signals'. https://www.sciencedirect.com/science/article/pii/S0040162517300707

Database: International HTA Database

Date searched: 25/01/2022

Search strategy: "horizon scanning" in title

Retrieved results: 310

Sifted on screen, none included
