# Supplementary material for: Looking at the fringes of MedTech innovation: a mapping review of horizon scanning and foresight methods
Source: BMJ Open. 2023 Sep 14;13(9):e073730. doi: 10.1136/bmjopen-2023-073730 (PMC10503360; doi:10.1136/bmjopen-2023-073730)
Supplement: Supplementary data [file bmjopen-2023-073730supp003.pdf]

Appendix C

Figure C.1. PRISMA flow chart

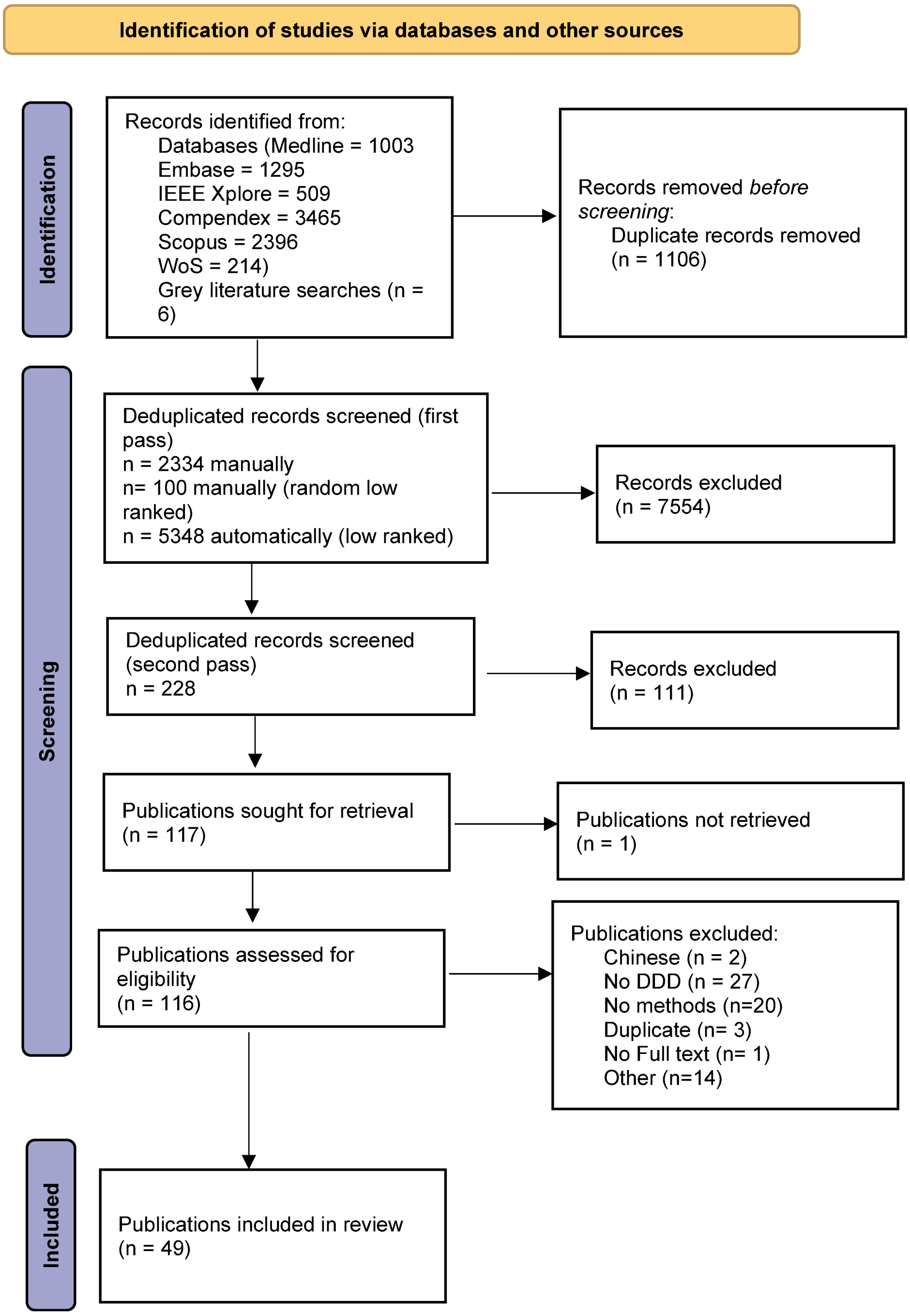

From: Page MJ, McKenzie JE, Bossuyt PM, Boutron I, Hoffmann TC, Mulrow CD, et al. The PRISMA 2020 statement: an updated guideline for reporting systematic reviews. BMJ 2021;372:n71. doi: 10.1136/bmj.n71
