# Supplementary material for: Looking at the fringes of MedTech innovation: a mapping review of horizon scanning and foresight methods
Source: BMJ Open. 2023 Sep 14;13(9):e073730. doi: 10.1136/bmjopen-2023-073730 (PMC10503360; doi:10.1136/bmjopen-2023-073730)
Supplement: Supplementary data [file bmjopen-2023-073730supp004.pdf]

Appendix D. Table 1: Included studies characteristics: methods, sources, technology types and time horizon

Authors	Organization/ Geography	Study Type	Main purpose	Methods used	Sources	Technology type	Horizon
**Apreda R, 2019**	University of Pisa and Research Institute for the Evaluation of Public Policies<br><br>(Italy)	Methods research paper	Early warning	Combination	Experts Stakeholders	Medical devices	Prospective<br><br>5-year horizon<br><br>Scan undertaken in 2010-2011
				Expert panel; Desk research on patents and literature searches for scientific publications; Functional analysis; Consultation of results with external stakeholders	Published and Unpublished literature		
					Patents		
**Baskici C, 2019**	Baskent University<br><br>(Turkey)	Conferen ce paper	Identify emerging trends	Combination	Experts	All	Retrospective<br><br>2013-2015 (retrospective search)
				Text mining; Web scrapping; Patent analysis; Expert consultation	Patents<br><br>Clinical trials		
					Published literature		

Authors	Organization/ Geography	Study Type	Main purpose	Methods used	Sources	Technology type	Horizon
**Bhavnani SP, 2017**	American College of Cardiology (USA)	Policy	Identify emerging trends	Combination	Experts	All (digital health, big data, and precision health)	Not specified
				Horizon scanning; Literature searches; network for gathering and sharing information	Published/ Unpublished literature		
**Brown IT, 2005**	Department of Electrical & Computer Systems Engineering, Monash University (Australia)	Published educatio nal note	Support decision making	Combination	Stakeholders; Experts	Medical devices for remote patient monitoring	Not specified
				Systematic literature review; Stakeholders' Survey; Experts consultation	Published literature		
					Approved commercial devices database		

Authors	Organization/ Geography	Study Type	Main purpose	Methods used	Sources	Technology type	Horizon
**Chalkidou A, 2019**	University College London (London, UK)	Conference abstract	Support decision making	Combination	Experts	MedTech: a hybrid theatre incorporating a biplane, intraoperative MRI (iMRI), multi-detector computed tomography (CT) scanners, and an EOS imaging system	Not specified
				Literature search; Experts	Published literature		
					Hospital data		
**Chang SWC, 2014**	National Tsing Hua University and National Chiao Tung University (Taiwan)	Conference paper	Identify emerging trends	Combination	Published literature	Dental implants	Retrospective 1998 - 2002
				Literature search; patent search	Patents		
**Dixon LC, 2016**	NIHR Horizon Scanning		Early warning	Combination	Clinical experts and patients	All (Devices, Diagnostics,	Prospective and Retrospective

Authors	Organization/ Geography	Study Type	Main purpose	Methods used	Sources	Technology type	Horizon
	Research & Intelligence Centre (HSRIC) (Birmingham, UK)	Horizon Scanning report		Literature search; Clinical experts; patients	Published/Unpublished literature	Digital and "Other")	Technologies licensed within the last 2 years or in development planned to launch within the next 18 months
**Douw K, 2003**	Collaboration between Public Health, HTA and HS organisations (Denmark & UK)	Methods research paper	Early warning	Single	Internet	All	Not specified
				Searching the internet			
**Flick C, 2020**	Collaboration across UK and non-UK universities and research institutes (EU)	Methods research paper	Identify future developments	Single	Published/Unpublished literature	All	Retrospective 2014-2015 (scan undertaken in 2015) and 2016-2017 (scan undertaken in 2017)
				Horizon scanning	Social media		

Authors	Organization/ Geography	Study Type	Main purpose	Methods used	Sources	Technology type	Horizon
**Gelderblom GJ, 2009**	European Commission Directorate General Information Society and Media (EU)	Conference paper	Identify emerging trends	Combination	Published (potentially including unpublished) literature	Robotics	Prospective 2009 to 2025
				Literature search; Expert and stakeholders survey and interviews; workshop for drafting the roadmap	Experts		
**Handa N, 2020**	Pharmaceuticals and Medical Devices Agency of Japan (PMDA) (Japan)	Horizon Scanning report	Technology assessment	Combination	Experts;	Artificial organs	Not specified
				Horizon scanning and expert consultation			
**Hussain M, 2017**	Aston University (Birmingham, UK)	Methods research paper	Identify future developments	Single	Stakeholders	Radio Frequency IDentification (RFID) technology	Not specified
				Scenario-Driven roadmapping			

Authors	Organization/ Geography	Study Type	Main purpose	Methods used	Sources	Technology type	Horizon
**Ibargoyen-Roteta N, 2009**	Horizon scanning organisation linked to EuroScan Network<br><br>(Spain)	Horizon Scanning methods paper	Support decision making	Single<br><br>Surveying other HS organizations	Stakeholders (other HS agencies)	Potentially Obsolete Technologies (POT)	Not applicable
**Itoh S, 2019**	Japan's Ministry of Health, Labour and Welfare and Ministry of Economy, Trade and Industry (METI) (MHLW)<br><br>(Japan)	Methods research paper	Support decision making	Single<br><br>Survey with experts, clinicians and companies	Stakeholders	Medical devices	Retrospective<br><br>2004 to 2014
**Lee J, 2019**	Researchers<br><br>(Korea)	Methods research paper	Identify business opportunities	Single<br><br>Twitter data related to health technology	Twitter	Medical health technologies including devices and digital health	Retrospective<br><br>January 2010 to October 2016
**Lerner JC, 2019**	ECRI Institute<br><br>(USA)	Horizon Scanning	Early warning	Single	Forecasts published by ECRI Institute	All (innovations that could	Retrospective<br><br>2007-2010

Authors	Organization/ Geography	Study Type	Main purpose	Methods used	Sources	Technology type	Horizon
		case study		Content analysis	used as case studies	create significant shifts in modes of care delivery, patient health outcomes, health care costs, technology utilization, and infrastructure needed to deliver care)	
**Migliore A, 2012**	Agenzia Nazionale per i Servizi Sanitari Regionali through its project COTE (Centro Osservazione Tecnologie sanitarie Emergenti) (Italy)	Horizon Scanning methods paper	Early warning	Single<br>Stakeholder engagement	Stakeholders	Medical devices	Not specified

Authors	Organization/ Geography	Study Type	Main purpose	Methods used	Sources	Technology type	Horizon
**Morrison A, 2012**	Canadian Agency for Drugs and Technologies in Health (CADTH) (Canada)	Horizon Scanning methods paper	Early warning	Combination	Stakeholders	Drug and non-drug medical health technologies including devices and diagnostics	Prospective 2 to 3 years to licence or diffuse
				Horizon scanning and environmental scans; Stakeholder engagement	Published/ Unpublished literature		
**Mühlroth C, 2020**	Friedrich-Alexander-Universität Erlangen-Nürnberg (Germany)	Methods research paper	Identify emerging trends	Single	Not reported	All	Not specified
				Strategic foresight			
**Mundy L, 2005**	Australia and New Zealand Horizon Scanning Network (ANZHSN) (Australia & New Zealand)	Horizon Scanning methods paper	Early warning	Single	Published (potentially including unpublished) literature	Medical devices, diagnostics and procedures	Prospective 3 years to licence
				Horizon Scanning	News		
					Regulatory information		

Authors	Organization/ Geography	Study Type	Main purpose	Methods used	Sources	Technology type	Horizon
**O'Malley S P, 2009**	Australia and New Zealand Horizon Scanning Network (ANZHSN) (Australia & New Zealand)	Horizon Scanning methods paper	Early warning	Single	Stakeholders (other HS agencies) Experts	Medical devices and diagnostics	Prospective > 10 years
				Horizon Scanning	Published/ Unpublished literature		
					News		
					Regulatory information		
**Zhao-Lian Ouyang Yu-Bo Fan, 2014**	Beijing University of Aeronautics & Astronautics and Chinese Academy of Medical Sciences (China)	Methods research paper	Identify future developments	Single	Patents	Medical radiation diagnosis devices	Retrospective 2003 to 2012
				Patent bibliometric analysis			
**Packer C, 2005**	NIHR Horizon Scanning Research & Intelligence Centre (HSRIC)	Horizon scanning methods paper	Technology assessment	Single	Experts; Industry; Stakeholders (other HS agencies)	All	Not specified

Authors	Organization/ Geography	Study Type	Main purpose	Methods used	Sources	Technology type	Horizon
	(Birmingham, UK)			Horizon Scanning	Published/Unpublished literature		
**Postma T, 2017**	University of Groningen and Dutch Cancer Society and Dutch Cancer Society (The Netherlands)	Methods research paper	Support decision making	Combination<br>Group expert opinion forecasting using the Delphi-technique, and a market-oriented capacity analysis using projective scenarios	Experts	All	Not specified
**Pretorius L, 2017**	University of Pretoria (South Africa)	Horizon Scanning case study	Adoption and diffusion	Single<br>Trend analysis	Bibliometric data from Google Scholar and SciDirect	Medical devices and diagnostic	Retrospective<br>10-15 years
**Robert G, 1999**	NHS Research and Development HTA programme	Horizon scanning	Early warning	Single	Experts	All	Not specified

Authors	Organization/ Geography	Study Type	Main purpose	Methods used	Sources	Technology type	Horizon
	(UK)	methods paper		Delphi technique			
**Rotolo D, 2015**	University of Sussex; University of Valencia; University of Amsterdam; University of Sussex (EU)	Methods research paper	Support decision making	Single	Published literature	All	Retrospective 1982 to 2011
				Scientometric mapping approach: overlay mapping	Patents		
**Ruggeri M, 2020**	National Centre for HTA, Istituto Superiore di Sanità (Italy)	Horizon scanning case study	Support decision making	Combination	Experts; Stakeholders	All (devices and digital apps)	Not specified
				Horizon scanning and experts panel			
**Sheikh NS, 2016**	Department of Technology and Society, University of Korea (Korea)	Conference paper	Identify emerging trends	Combination	Bibliometric data	Devices (biosensor technologies based on human saliva, breath or blood)	Not specified
				Bibliometric and trend analyses	Patents		

Authors	Organization/ Geography	Study Type	Main purpose	Methods used	Sources	Technology type	Horizon
**Shekelle PG, 2018**	Researchers at University of Southern California Institutional Report Board and the RAND Corporation Human Subject Protection Committee (USA)	Methods research paper	Planning & Evaluation	Combination	Experts	Medical devices and diagnostics	Retrospective analysis of a prospective prediction 20 years
				Systematic literature searches and multidisciplinary experts	Published (potentially including unpublished) literature		
**Shen Y C, 2020**	Research department of Biobusiness Management (Taiwan)	Methods research paper	Identify business opportunities	Single	Published literature	Medical devices	Retrospective from database inception to 2015
				Literature search and patent search	Patents		
**Simpson S, 2018**	NIHR Horizon Scanning Research &	Methods research paper	Early warning	Combination	Patients and the Public	All	Not specified

Authors	Organization/ Geography	Study Type	Main purpose	Methods used	Sources	Technology type	Horizon
	Intelligence Centre (HSRIC)<br>(Birmingham, UK)			Horizon scanning and PPIE			
**Smith J, 2015**	NIHR Horizon Scanning Research & Intelligence Centre (HSRIC)<br>(Birmingham, UK)	Horizon scanning report	Technology assessment	Combination<br>Horizon scanning, consultation with clinical experts and PPIE	Patients and the Public<br>Experts	All	Not specified
**de Souza Antunes AM, 2012**	Instituto Nacional de Propriedade Industrial<br>(Brazil)	Methods research paper	Identify emerging trends	Single<br>Patent trend analysis	Patents	Nanotechnology applied to the screening, diagnosis, and treatment of disease	Retrospective<br>2000 to 2010
**Stafinski T, 2020**	Canadian Agency for Drugs and Technologies in Health (CADTH)<br>(Canada)	Methods research paper	Support decision making	Single<br>Expert consultation	Experts	All	Prospective<br>5 years ahead

Authors	Organization/<br>Geography	Study Type	Main purpose	Methods used	Sources	Technology type	Horizon
**Storz P, 2007**	German HTA in collaboration with the Federal Association of the Local Health Insurance Funds (AOK Bundesverband)<br>(Germany)	Horizon scanning report	Support decision making	Combination	Published/<br>Unpublished literature	Genetic testing	Not specified
				Horizon Scanning; Literature searching	Internet<br>ZIM (private) database		
**Takayoshi H, 2017**	VALUENEX Japan Inc<br>(Japan)	Methods research paper	Identify emerging trends	Single	Patents	Artificial intelligence applied to health and wellbeing	Retrospective<br>Published between 2001 and 2016
				Panoramic view analytics			
**Tal O, 2012**	Israeli Center for Technology Assessment in Health Care (ICTAHC)<br>(Israel)	Horizon Scanning methods paper	Technology assessment	Single	Experts<br>Industry	Medical devices	Prospective<br>Three horizons: short term 1 year; medium term and long-term
				Horizon scanning using 3 time horizons	Published/		

Authors	Organization/ Geography	Study Type	Main purpose	Methods used	Sources	Technology type	Horizon
					Unpublished literature<br>Internet<br>Clinical trials		
**Tark YJ, 2015**	Horizon Scanning Service of Innovative Global Health Technology (H-SIGHT)<br>(South Korea)	Horizon scanning report	Predict impact in healthcare services	Combination<br>Horizon scanning; Literature report; Clinical trials search	Published literature<br>Clinical trials	Medical device (polyurethane scaffolds)	Retrospective evidence search<br>Prospective prediction of impact (1 to 5 years) from launch
**Thorleucht er D, 2013**	Fraunhofer Institute for Technological Trend Analysis INT<br>(Germany)	Methods research paper	Identify emerging trends	Single<br>Web mining and latent semantic indexing	Internet	All	Not specified
**Trappey CV, 2014**	National Tsing Hua University and National		Identify future developments	Combination	Patents	Dental implant connectors	Retrospective (published

Authors	Organization/ Geography	Study Type	Main purpose	Methods used	Sources	Technology type	Horizon
	Chiao Tung University (Taiwan)	Methods research paper		Text mining and literature searches	Published clinical trials		between 1992 and 2012)
**Trevitt S, 2016**	NIHR Horizon Scanning Research & Intelligence Centre (HSRIC) (Birmingham, UK)	Horizon Scanning report	Early warning	Single	Published/ Unpublished literature	Wearable medical devices second and third generation Artificial Pancreas Device systems	Not specified
				Horizon scanning	Clinical trials		
					Internet; Internal databases; Third party databases; Manufacturer's websites		
**Vaganova EV, 2015**	National Research Tomsk State University (Russia)	Conference paper	Identify emerging trends	Single	Published literature	Medical devices and digital interventions (e-health and m-health, telemedicine and personalised smart devices)	Not specified
				Forecasting	Patents		

Authors	Organization/ Geography	Study Type	Main purpose	Methods used	Sources	Technology type	Horizon
**Varela-Lema L, 2012**	Galician Agency for Health Technology Assessment (avalia-t) (Spain)	Methods research paper	Identify emerging trends	Combination	Experts	Medical devices, procedures and diagnostics	Retrospective (4 to 5 years)
				Horizon scanning and expert consultation	Published/ Unpublished literature		
**Varela-Lema L, 2014**	Galician Agency for Health Technology Assessment (avalia-t) (Spain)	Methods research paper	Identify emerging trends	Single	Published literature	Medical devices, procedures and diagnostics	Prospective (1 to 2 years from availability)
				Bibliographic database search			
**Verbakel Jan Y, 2017**	National Institute for Health Research (NIHR) Diagnostic Evidence Co-operative Oxford at Oxford Health Foundation Trust (Oxford, UK)	Horizon scanning report	Identify evidence gaps	Single	Horizon scanning reports	Diagnostic tests	Not specified
				Content analysis			
				Single	Patents	Telehealth	

Authors	Organization/ Geography	Study Type	Main purpose	Methods used	Sources	Technology type	Horizon
**Wang J, 2019**	National Chung Hsing University (Taiwan)	Methods research paper	Identify business opportunities	Patent trend analysis			Retrospective (1991 to 2015)
**Wong Q W, 2020**	Singapore HS Organization (Singapore)	Methods research paper	Early warning	Single<br>Horizon Scanning	Commercial developer websites<br>Regulatory authorities (FDA and EMA)<br>Medical news media; Scientific journals; Conference proceedings<br>Reports from other horizon scanning organizations (CADTH, NIHR IO)<br>Trial registries	All (disruptive medicines, devices, digital and diagnostics)	Prospective (3 years to regulatory approval for drugs, different time horizon for DDD)
**Ye C, 2013**				Single			Not specified

Authors	Organization/ Geography	Study Type	Main purpose	Methods used	Sources	Technology type	Horizon
	Beijing City University (China)	Conferen ce paper	Identify future developments	Technology roadmap	Nanotechnology Roadmap of U.S. NASA	All (not specified)	
