# Supplementary material for: Looking at the fringes of MedTech innovation: a mapping review of horizon scanning and foresight methods
Source: BMJ Open. 2023 Sep 14;13(9):e073730. doi: 10.1136/bmjopen-2023-073730 (PMC10503360; doi:10.1136/bmjopen-2023-073730)
Supplement: Author's manuscript [file bmjopen-2023-073730.draft_revisions.pdf]

BMJ Open

BMJ Open is committed to open peer review. As part of this commitment we make the peer review history of every article we publish publicly available.

When an article is published we post the peer reviewers' comments and the authors' responses online. We also post the versions of the paper that were used during peer review. These are the versions that the peer review comments apply to.

The versions of the paper that follow are the versions that were submitted during the peer review process. They are not the versions of record or the final published versions. They should not be cited or distributed as the published version of this manuscript.

BMJ Open is an open access journal and the full, final, typeset and author-corrected version of record of the manuscript is available on our site with no access controls, subscription charges or pay-per-view fees (http://bmjopen.bmj.com).

If you have any questions on BMJ Open's open peer review process please email info.bmjopen@bmj.com

BMJ Open

Looking at the fringes of MedTech innovation: a literature review of horizon scanning and foresight methods

Journal:	*BMJ Open*
Manuscript ID	bmjopen-2023-073730
Article Type:	Original research
Date Submitted by the Author:	16-Mar-2023
Complete List of Authors:	Garcia Gonzalez-Moral, Sonia; Newcastle University, NIHR Innovation Observatory at Population Health Sciences Institute<br>Beyer, Fiona ; Newcastle University, NIHR Innovation Observatory at Population Health Sciences Institute<br>Oyewole, Anne; Newcastle University, Population Health Sciences Institute<br>Richmond, Catherine; Newcastle University, NIHR Innovation Observatory at Population Health Sciences Institute<br>Wainwright, Luke; Newcastle University, Population Health Sciences Institute<br>Craig, Dawn; Newcastle University, NIHR Innovation Observatory at Population Health Sciences Institute
Keywords:	Decision Making, Health policy < HEALTH SERVICES ADMINISTRATION & MANAGEMENT, BIOTECHNOLOGY & BIOINFORMATICS

SCHOLARONE™
Manuscripts

I, the Submitting Author has the right to grant and does grant on behalf of all authors of the Work (as defined in the below author licence), an exclusive licence and/or a non-exclusive licence for contributions from authors who are: i) UK Crown employees; ii) where BMJ has agreed a CC-BY licence shall apply, and/or iii) in accordance with the terms applicable for US Federal Government officers or employees acting as part of their official duties; on a worldwide, perpetual, irrevocable, royalty-free basis to BMJ Publishing Group Ltd ("BMJ") its licensees and where the relevant Journal is co-owned by BMJ to the co-owners of the Journal, to publish the Work in this journal and any other BMJ products and to exploit all rights, as set out in our licence.

The Submitting Author accepts and understands that any supply made under these terms is made by BMJ to the Submitting Author unless you are acting as an employee on behalf of your employer or a postgraduate student of an affiliated institution which is paying any applicable article publishing charge ("APC") for Open Access articles. Where the Submitting Author wishes to make the Work available on an Open Access basis (and intends to pay the relevant APC), the terms of reuse of such Open Access shall be governed by a Creative Commons licence – details of these licences and which Creative Commons licence will apply to this Work are set out in our licence referred to above.

Other than as permitted in any relevant BMJ Author's Self Archiving Policies, I confirm this Work has not been accepted for publication elsewhere, is not being considered for publication elsewhere and does not duplicate material already published. I confirm all authors consent to publication of this Work and authorise the granting of this licence.

For peer review only

Title: Looking at the fringes of MedTech innovation: a literature review of horizon scanning and foresight methods

Authors:

Lead and Corresponding author: Gonzalez-Moral, Sonia G[1] Dip(HE) LIS, BSc (Hons), MSc, ORCID 0000-0003-0431-4771 Sonia.garcia-gonzalez-moral@ncl.ac.uk

Second Author: Beyer, Fiona R[1] PGDip Information and Library Management, ORCID 0000-0002-6396-3467 Fiona.beyer@ncl.ac.uk

Third Author: Oyewole, Anne O[2] PhD, MRes, Bsc ORCID 0000-0002-1311-1485 Anne.oyewole@newcastle.ac.uk

Fourth Author: Richmond, Catherine[1] BSc (Hons), MA wassatwork@gmail.com

Fifth Author: Wainwright, Luke[2] PhD ORCID 0000-0003-3742-6411 luke.wainwright@nhs.net

Last Author: Craig, Dawn[1] Professor of Evidence Synthesis, ORCID 0000-0002-5808-0096 Dawn.craig@ncl.ac.uk

Abstract

Horizon-scanning (HS) is a method used to examine signs of change and may be used in foresight practice. HS methods used for the identification of innovative medicinal products cannot be applied in MedTech due to differences in development and regulatory processes.

This literature review aims to identify HS and other methodologies used for MedTech foresight. Database and grey literature searches identified 8,888 records. After deduplication, manual and automated title, abstracts and full-text screening, forty-nine papers met the inclusion criteria and were data extracted.

Twenty-five single different methods were identified, often used in combination, of these, only three were novel (appearing only once in the literature). Text mining or artificial intelligence solutions appear as early as 2012, often practiced in patent and social media sources. The time horizon used in scanning was not

[1] NIHR Innovation Observatory, Population Health Sciences Institute, Newcastle University
[2] Visiting Researcher, Population Health Sciences Institute, Newcastle University

For peer review only

often justified. Some studies regarded experts both as a source and as a method. Literature searching remains one of the most used methods for innovation identification. HS methods were vaguely reported, but often involved consulting with experts and stakeholders.

HS is a complex time-consuming process that requires a range of skills, knowledge and access to great amount of data for identification, interpretation, and filtration of signals. This review revealed an array of known methods being used in combination to overcome limitations posed by single methods. The review also revealed inconsistency in methods reporting, with a lack of any consensus regarding best practice. Greater transparency in methods reporting and consistency in methods use would contribute to increased output quality to support informed timely decision-making.

Strengths and limitations of this study

- This review updates the methodological knowledge on horizon scanning practice for health and care decision-making of MedTech innovations.
- This is the first time a systematic literature search is expanded beyond traditional horizon scanning methods to identify other foresight methods applied in MedTech innovation detection and prediction.
- Quality appraisal of the included studies was deemed out of scope and was not performed; however, the systematic lack of reporting methods was manifested as a constant methodological limitation throughout this review.

Keywords:

Review; Foresight; Horizon Scanning; Medical Technologies; Technology Assessment, Biomedical; Decision Making

Funding:

This study/project is funded by the National Institute for Health Research (NIHR) [NIHRIO/project reference HSRIC-2016-10009]. The views expressed are those of the author(s) and not necessarily those of the NIHR or the Department of Health and Social Care.

For peer review only

Word count: 3795

Introduction

Horizon-scanning (HS) is a reliable method to inform legal, regulatory or procurement decisions on new and emerging health technologies.[1,2] The strategic importance of HS is recognised and endorsed at the highest level; for example, in the UK, the 2016 independent Accelerated Access Review recommended that the NHS should develop an enhanced HS process to identify products that have the most potential to deliver improved outcomes or efficiencies, acknowledging a more comprehensive and transparent HS system is necessary, especially for non-pharmaceutical products. This task was mandated to the NHS Accelerated Access Collaborative (AAC) by the Secretary of State in 2018. The 2019 'NHS Long Term Plan' sets out to accelerate innovation and patient access to innovative therapies.[3] More recently the 2021 Government's 'Life Sciences Vision' policy statement aims to stimulate industry growth and success for the benefit of patients and the NHS.[4] Both high level national policies advocate for the use of HS to achieve their goals, a sentiment that is echoed in the Government's 'Saving and Improving Lives: The Future of UK Clinical Research Delivery' policy paper (2021) and associated Implementation Plan (2022) which reinforces the AAC's commitment 'to improve identification of the most needed treatments and technologies and rapidly bring these into clinical use' through horizon scanning.

Medical technologies (MedTech) have the potential to transform care pathways, improve health and contribute to cost efficiencies in the healthcare system.[5] HS methods used to signal new medicinal products cannot be directly translated and applied to MedTech due to the challenges posed by the intrinsic characteristics of these technologies' development and regulatory pathways. These challenges are wide ranging and multi-layered, however for the purposes of this paper we will concentrate on those that affect the identification of these technologies ahead of their placement on the market. Firstly, in order to discern where the true value for the healthcare system lies a common definition and interpretation of 'innovation' is needed.[6] Innovation in MedTech may encompass a completely new device class or newer iterations of the same product.[7] Secondly, different regulators of MedTech follow different criteria and requisites for their assessment. These differences have an impact on how and when information is shared with regulators, and whether it can be made available to the wider public. In turn, this has an impact on the

For peer review only - http://bmjopen.bmj.com/site/about/guidelines.xhtml

ability of HS searches to retrieve and identify signals of commercial readiness. For example, whilst the US Food and Drug Administration (FDA) maintains medical devices databases that can be freely accessed,[8] the EU regulator has not made this information available to the wider public yet.[9] Thirdly, traditional published literature sources might not fully contribute to identify emerging or early innovative MedTech. Whilst this is also the case in HS practice for innovative medicinal products, for MedTech, grey literature, mainly originated by manufacturers, acts as the main source in up to 50% of the time.[10]

Foresight and HS methods share similar concepts and purpose but differ as follows. The objectives of foresight activities can vary across projects but generally aim to open discussion and debate in anticipation of strategy formulation with the aim of equipping organisations navigating an uncertain future. In HS, however, the aim is the identification of emerging issues or signals and their targets and connections within to elicit a plan of action.[11] Well-conducted HS is a complex and time-consuming process that requires a range of skills and a depth of knowledge to facilitate the identification, interpretation, and filtration of relevant signals, in addition to highly developed technological tools to support the processing and management of data and knowledge and facilitate its dissemination in a timely manner. Other analytical foresight methods such as patent, bibliometric and trend analyses as well as non-analytical methods such as backcasting, roadmaps or Delphi studies are available that could complement or help solve some of these challenges when applied to the identification and prediction of MedTech innovation, but to date there is no evidence to support their use.

Previous reviews into HS methods in healthcare have been undertaken[1,12] however, none of them have looked at how these methods perform when applied to the MedTech innovation pipeline. This literature review aims to build from what is already known by broadening the scope to identify foresight methods applied to MedTech innovation identification or prediction, including but not limited to HS; and, to explore the drivers that lead to the application of these methodologies in the context of MedTech to gain an insight into the rationale for choosing a particular method over others. This work is the first step for devising a methodological framework to guide foresight practice in MedTech innovation decision-making.

Methods

For peer review only

In line with the core principles for conducting mapping reviews outlined by the Campbell Collaboration[13], this review aimed to identify evidence of use of HS and foresight methods for MedTech innovation detection. A protocol was agreed amongst the review team which guided the inclusion and exclusion of studies as well as the systematic literature searching in databases and grey literature sources. This review did not undertake quality appraisal of the included studies as this was considered out of scope. PRISMA-S extension for reporting systematic literature searches was followed and reported in Appendix A, Table A.1.[14]

Patient and public involvement

Members of the public were not involved in this review.

Inclusion criteria

Studies were eligible for inclusion if: i) any of the following methods were mentioned in the text: environmental scanning, HS, foresight, forecasting, roadmapping, patent, bibliometric or trend analyses, backcasting or Delphi studies; ii) a medical health technology such as a medical device, diagnostic, digital intervention, or all was included; iii) published in English.

Search strategy

The search strategy consisted of a combination of subject headings and text words in title, abstracts and key word fields. The strategy was peer-reviewed by an independent information specialist using the PRESS review statement.[15] The final MEDLINE strategy was translated and run separately in: Embase, Scopus, Web of Science, IEEE Xplore and Compendex Engineering Village. Grey literature sources (Google and CORE[16]) and International Horizon Scanning organizations (via International HTA Database) were searched. No time or language limits were used. Detailed search strategies are included in Appendix B. Reference lists of included papers were browsed to identify potential papers missed. Search results were downloaded to Endnote20 (Clarivate Analytics) and deduplicated.

Data collection

For peer review only

For peer review only - http://bmjopen.bmj.com/site/about/guidelines.xhtml

Results were de-duplicated and screened in two stages using Rayyan, a semi-automated screening tool for systematic reviews.[17] At stage one the main reviewer screened the title and abstracts, with each vote for inclusion or exclusion, Rayyan produced a 5-star ranking for all the references uploaded. A random sample of 100 of low-ranked (three stars or less) references were screened to assess the consensus of the semi-automated ranking with the reviewer's manual screening decisions.

At second stage, two reviewers manually double screened the full texts and selected those that met the eligibility criteria. Disagreements were reconciled by discussion between the two reviewers.

Data extraction

A custom data extraction form was designed in Excel to record the relevant characteristics of the included papers. The extracted data captured: 1) author name and publication year, 2) title, 3) objective of the article, 4) study type, 5) methods used, 6) sources used, 7) technology type, 8) time horizon, 9) type of output produced and 10) automated methods used.

Data synthesis

Extracted data was further categorised by objectives described and information on methods used to allow for analysis as some discrepancies in the reporting were present. Data was synthesised visually, tabulated and in narrative formats.

Results

Database and grey literature searches identified 8888 records. After de-duplication 7782 remained. The first screening pass included a total of 228 references (more details on this stage on Appendix C, fig.C.1). Two reviewers manually screened the title and abstract of these and excluded a further 111 references. At second stage, 117 full-text papers were manually double screened, concluding with 49 papers published between 1999 and 2020 that met the inclusion criteria. PRISMA flow-chart included in Appendix C, fig. C.1.

Analysis of objectives

Ten different categories of aims and objectives were identified (fig. 1).

Figure 1. Aims and objectives for using HS/foresight methods in MedTech innovation identification

For peer review only

Early warning[18-29], identification of emerging trends[30-41] with supporting decision-making[42-50] were the most frequently reported reasons for the use of HS or foresight methods followed by identification of future developments,[51-55] supporting technology assessment processes[56-59] and identification of business opportunities[60-62]. More scarcely used were early assessment to predict the impact on health care services,[63] group judgement process for forecasts to support planning and evaluation,[64] a case study in electromagnetic field using forecasts for prediction of technology adoption and diffusion[65] and, lastly, the identification of evidence gaps by the analysis of published HS reviews.[66]

Methods and sources

The included studies reported 25 single different methods. Twenty-nine studies used a single method[20-22,24-26,28,29,33,36,38,39,41,44,45,47,49,50,53,55,57,59,60,62,65,66] and 20 used multiple methods in combination.[18,19,23,30-32,34,37,40,42,43,46,48,51,54,56,58,61,63,64] The methods that overlap between the group of papers that reported using one method and the group the used more than one in combination were: expert panels, patent searches/analyses, literature searches, stakeholders, HS, roadmapping, Delphi studies, trend analyses, patient engagement and text mining. A small number of uniquely used methods in the combination group encompassed clinical trial analysis, searching approved medical devices databases, hospital data analysis, environmental scan and projective scenarios; while other were uniquely employed by the single-method group: content analysis, forecasting, internet searching, forecast report analysis, panoramic view analytics, scenario-driven road mapping, scientometric mapping approach to overlay mapping, strategic foresight or surveying other horizon scanning organisations.

Figure 2. Methods and sources overlap

Figure 2 represents a Venn diagram for the overlap of methods as reported, the black writing denotes sources and red and purple denotes methods. Methods and sources were often not differentiated. Across both groups, the most frequent and clearly reported method was HS (n=15) followed by surveying or consulting with experts (n=14), literature searching (n=13), patent searching/analysis (n=10), stakeholder engagement (n=4), text/data mining (n=4), trend analysis (n=3), and roadmaps (n=3). Cited less often

For peer review only

were report content analyses (n=2),[21,66] Delphi (n=2),[26,46] clinical trial searches (n=2),[30,63] web scraping or mining (n=2),[30,38] and social media searching (n=2).[51,60]

Methods mentioned only once included: environmental scanning,[23] overlay mapping,[47] panoramic view analytics,[35] strategic foresight,[36] surveying companies[45] and surveying other HS organisations.[44]

Forecasting was mentioned by four different reports.[21,39,46,65] One used a combination of expert group opinion forecasting approach, i.e. the Delphi-technique with a market oriented capacity analysis by using projective scenarios.[46] The three remaining cite forecasting as a single method using multiple sources including literature and patent searches.[21,39,65]

Less clearly reported methods, often embedded in the reporting of the sources used, were engagement with stakeholders (7)[18,22,23,42,45,48,52] and surveying or consulting with patients (3).[19,27,58]

Eleven reports published between 2012 and 2020 reported the use of automated methods.[30,33,35,36,38,39,54,55,60-62] The most frequently cited was text mining with seven reports describing its use; detailed descriptions of the software packages used was not provided in all instances. Web scraping or URL crawling was described by two reports separately, machine learning (including unsupervised mode) was cited by two reports, data mining was mentioned by two reports and natural language processing (NLP) by one.

The great majority of the included reports (88%) described the sources of data/intelligence used (fig 3).

Figure 3. Type of sources used as described in the included reports

Thirteen reports described the use of published literature sources.[24,30,32,34,39,40,42,43,47,54,61,63,64] Of these, 3 did not specify whether they also searched unpublished literature [24,34,64] and a further 12 used published and unpublished sources of literature.[18,19,23,25,28,29,31,41,50,51,57,59]

Literature searching was used in different ways to support the identification of signals. Whilst some reports used it as the basis of the signal identification,[19,23,28,30,40-42,64] others used it as a complement to enhance the strength of signals already identified by other means,[18,31,43,50,63] some reports used it in combination

with other sources to study relationships[32,39,47,54,67] or expand the signal identification process.[24,25,29,34,51,57,59]

All studies that reported literature searching as a method also used it as a source, however some studies cited literature searching as a source but did not describe it as a method.[23-25,28,29,39,47,50,54,57,59]

The analysis of methods reported in the literature in conjunction with the sources used, identified seven reports[24,25,28,29,50,57,59] that used HS as a single method whilst searching multiple sources. Three of those reports[25,57,59] additionally practised some level of expert, stakeholder or patient engagement.

From the group of nine papers that reported the use of HS in combination with other methods, seven included consultation with experts, stakeholders or patients.[23,27,31,41,48,56,58] Since half of those studies that reported using HS as a method also reported the use of experts or stakeholders, it cannot be extrapolated that HS has always involved consulting with experts unless explicitly specified.

Types of technologies included

The most frequently searched for technology were medical devices (n=28), followed by 16 diagnostics, nine digital health interventions and three procedures. Four reports included medical devices, diagnostics and digital health interventions.[19,29,37]

Fifteen reports recorded the inclusion of all technologies but did not provide details and have been analysed as “all inclusive”.[20,21,26,27,30,31,38,44,46,47,49,51,55,57,58]

Two reports did not specify any technology within the MedTech scope.[25,36]. Table 1 presents an overview of the included reports in relation to the technologies in scope and the main purpose of the study.

This review did not identify a relationship between the technologies in scope and the method used for their identification.

For peer review only

Table 1. Characteristics of included studies

Authors	Organization/ Geography	Study Type	Main purpose	Methods used	Sources	Technology type	Horizon
**Apreda R, 2019**	University of Pisa and Research Institute for the Evaluation of Public Policies<br>(Italy)	Methods research paper	Early warning	Combination	Experts<br>Stakeholders	Medical devices	Prospective<br>5-year horizon<br>Scan undertaken in 2010-2011
				Expert panel; Desk research on patents and literature searches for scientific publications; Functional analysis; Consultation of results with external stakeholders	Published and Unpublished literature		
					Patents		
**Baskici C, 2019**	Baskent University<br>(Turkey)	Conference paper	Identify emerging trends	Combination	Experts	All	Retrospective<br>2013-2015 (retrospective search)
				Text mining; Web scrapping; Patent analysis; Expert consultation	Patents<br>Clinical trials		
					Published literature		

For peer review only

Authors	Organization/ Geography	Study Type	Main purpose	Methods used	Sources	Technology type	Horizon
**Bhavnani SP, 2017**	American College of Cardiology (USA)	Policy	Identify emerging trends	Combination	Experts	All (digital health, big data, and precision health)	Not specified
				Horizon scanning; Literature searches; network for gathering and sharing information	Published/ Unpublished literature		
**Brown IT, 2005**	Department of Electrical & Computer Systems Engineering, Monash University (Australia)	Published educatio nal note	Support decision making	Combination	Stakeholders; Experts	Medical devices for remote patient monitoring	Not specified
				Systematic literature review; Stakeholders' Survey; Experts consultation	Published literature		
					Approved commercial devices database		

For peer review only

Authors	Organization/ Geography	Study Type	Main purpose	Methods used	Sources	Technology type	Horizon
**Chalkidou A, 2019**	University College London (London, UK)	Conference abstract	Support decision making	Combination	Experts	MedTech: a hybrid theatre incorporating a biplane, intraoperative MRI (iMRI), multi-detector computed tomography (CT) scanners, and an EOS imaging system	Not specified
				Literature search; Experts	Published literature		
					Hospital data		
**Chang SWC, 2014**	National Tsing Hua University and National Chiao Tung University (Taiwan)	Conference paper	Identify emerging trends	Combination	Published literature	Dental implants	Retrospective 1998 - 2002
				Literature search; patent search	Patents		
**Dixon LC, 2016**	NIHR Horizon Scanning	Horizon Scanning	Early warning	Combination	Clinical experts and patients	All (Devices, Diagnostics,	Prospective and Retrospective

For peer review only

Authors	Organization/ Geography	Study Type	Main purpose	Methods used	Sources	Technology type	Horizon
	Research & Intelligence Centre (HSRIC) (Birmingham, UK)	report		Literature search; Clinical experts; patients	Published/Unpublished literature	Digital and "Other")	Technologies licensed within the last 2 years or in development planned to launch within the next 18 months
**Douw K, 2003**	Collaboration between Public Health, HTA and HS organisations (Denmark & UK)	Methods research paper	Early warning	Single	Internet	All	Not specified
				Searching the internet			
**Flick C, 2020**	Collaboration across UK and non-UK universities and research institutes (EU)	Methods research paper	Identify future developments	Single	Published/Unpublished literature	All	Retrospective 2014-2015 (scan undertaken in 2015) and 2016-2017 (scan undertaken in 2017)
				Horizon scanning	Social media		

For peer review only

Authors	Organization/ Geography	Study Type	Main purpose	Methods used	Sources	Technology type	Horizon
**Gelderblom GJ, 2009**	European Commission Directorate General Information Society and Media (EU)	Conference paper	Identify emerging trends	Combination	Published (potentially including unpublished) literature	Robotics	Prospective 2009 to 2025
				Literature search; Expert and stakeholders survey and interviews; workshop for drafting the roadmap	Experts		
**Handa N, 2020**	Pharmaceuticals and Medical Devices Agency of Japan (PMDA) (Japan)	Horizon Scanning report	Technology assessment	Combination	Experts;	Artificial organs	Not specified
				Horizon scanning and expert consultation			
**Hussain M, 2017**	Aston University (Birmingham, UK)	Methods research paper	Identify future developments	Single	Stakeholders	Radio Frequency IDentification (RFID) technology	Not specified
				Scenario-Driven roadmapping			

For peer review only

Authors	Organization/ Geography	Study Type	Main purpose	Methods used	Sources	Technology type	Horizon
**Ibargoyen-Roteta N, 2009**	Horizon scanning organisation linked to EuroScan Network (Spain)	Horizon Scanning methods paper	Support decision making	Single<br>Surveying other HS organizations	Stakeholders (other HS agencies)	Potentially Obsolete Technologies (POT)	Not applicable
**Itoh S, 2019**	Japan's Ministry of Health, Labour and Welfare and Ministry of Economy, Trade and Industry (METI) (MHLW) (Japan)	Methods research paper	Support decision making	Single<br>Survey with experts, clinicians and companies	Stakeholders	Medical devices	Retrospective<br>2004 to 2014
**Lee J, 2019**	Researchers (Korea)	Methods research paper	Identify business opportunities	Single<br>Twitter data related to health technology	Twitter	Medical health technologies including devices and digital health	Retrospective<br>January 2010 to October 2016
**Lerner JC, 2019**	ECRI Institute (USA)	Horizon Scanning case	Early warning	Single	Forecasts published by ECRI Institute	All (innovations that could	Retrospective<br>2007-2010

For peer review only

Authors	Organization/ Geography	Study Type	Main purpose	Methods used	Sources	Technology type	Horizon
		study		Content analysis	used as case studies	create significant shifts in modes of care delivery, patient health outcomes, health care costs, technology utilization, and infrastructure needed to deliver care)	
**Migliore A, 2012**	Agenzia Nazionale per i Servizi Sanitari Regionali through its project COTE (Centro Osservazione Tecnologie sanitarie Emergenti) (Italy)	Horizon Scanning methods paper	Early warning	Single<br>Stakeholder engagement	Stakeholders	Medical devices	Not specified

For peer review only

Authors	Organization/ Geography	Study Type	Main purpose	Methods used	Sources	Technology type	Horizon
**Morrison A, 2012**	Canadian Agency for Drugs and Technologies in Health (CADTH)<br>(Canada)	Horizon Scanning methods paper	Early warning	Combination	Stakeholders	Drug and non-drug medical health technologies including devices and diagnostics	Prospective<br>2 to 3 years to licence or diffuse
				Horizon scanning and environmental scans; Stakeholder engagement	Published/<br>Unpublished literature		
**Mühlroth C, 2020**	Friedrich-Alexander-Universität Erlangen-Nürnberg<br>(Germany)	Methods research paper	Identify emerging trends	Single	Not reported	All	Not specified
				Strategic foresight			
**Mundy L, 2005**	Australia and New Zealand Horizon Scanning Network (ANZHSN)<br>(Australia & New Zealand)	Horizon Scanning methods paper	Early warning	Single	Published (potentially including unpublished) literature	Medical devices, diagnostics and procedures	Prospective<br>3 years to licence
				Horizon Scanning	News		
					Regulatory information		

For peer review only

Authors	Organization/ Geography	Study Type	Main purpose	Methods used	Sources	Technology type	Horizon
**O'Malley S P, 2009**	Australia and New Zealand Horizon Scanning Network (ANZHSN)<br><br>(Australia & New Zealand)	Horizon Scanning methods paper	Early warning	Single	Stakeholders (other HS agencies)<br><br>Experts	Medical devices and diagnostics	Prospective<br><br>> 10 years
				Horizon Scanning	Published/<br><br>Unpublished literature		
					News		
					Regulatory information		
**Zhao-Lian Ouyang Yu-Bo Fan, 2014**	Beijing University of Aeronautics & Astronautics and Chinese Academy of Medical Sciences<br><br>(China)	Methods research paper	Identify future developments	Single	Patents	Medical radiation diagnosis devices	Retrospective<br><br>2003 to 2012
				Patent bibliometric analysis			
**Packer C, 2005**	NIHR Horizon Scanning Research & Intelligence Centre (HSRIC)	Horizon scanning methods paper	Technology assessment	Single	Experts; Industry; Stakeholders (other HS agencies)	All	Not specified

For peer review only

Authors	Organization/ Geography	Study Type	Main purpose	Methods used	Sources	Technology type	Horizon
	(Birmingham, UK)			Horizon Scanning	Published/Unpublished literature		
**Postma T, 2017**	University of Groningen and Dutch Cancer Society and Dutch Cancer Society (The Netherlands)	Methods research paper	Support decision making	Combination<br>Group expert opinion forecasting using the Delphi-technique, and a market-oriented capacity analysis using projective scenarios	Experts	All	Not specified
**Pretorius L, 2017**	University of Pretoria (South Africa)	Horizon Scanning case study	Adoption and diffusion	Single<br>Trend analysis	Bibliometric data from Google Scholar and SciDirect	Medical devices and diagnostic	Retrospective<br>10-15 years
**Robert G, 1999**	NHS Research and Development HTA programme	Horizon scanning methods	Early warning	Single	Experts	All	Not specified

For peer review only

Authors	Organization/ Geography	Study Type	Main purpose	Methods used	Sources	Technology type	Horizon
	(UK)	paper		Delphi technique			
**Rotolo D, 2015**	University of Sussex; University of Valencia; University of Amsterdam; University of Sussex (EU)	Methods research paper	Support decision making	Single	Published literature	All	Retrospective 1982 to 2011
				Scientometric mapping approach: overlay mapping	Patents		
**Ruggeri M, 2020**	National Centre for HTA, Istituto Superiore di Sanità (Italy)	Horizon scanning case study	Support decision making	Combination	Experts; Stakeholders	All (devices and digital apps)	Not specified
				Horizon scanning and experts panel			
**Sheikh NS, 2016**	Department of Technology and Society, University of Korea (Korea)	Conference paper	Identify emerging trends	Combination	Bibliometric data	Devices (biosensor technologies based on human saliva, breath or blood)	Not specified
				Bibliometric and trend analyses	Patents		

For peer review only

Authors	Organization/ Geography	Study Type	Main purpose	Methods used	Sources	Technology type	Horizon
**Shekelle PG, 2018**	Researchers at University of Southern California Institutional Report Board and the RAND Corporation Human Subject Protection Committee (USA)	Methods research paper	Planning & Evaluation	Combination	Experts	Medical devices and diagnostics	Retrospective analysis of a prospective prediction 20 years
				Systematic literature searches and multidisciplinary experts	Published (potentially including unpublished) literature		
**Shen Y C, 2020**	Research department of Biobusiness Management (Taiwan)	Methods research paper	Identify business opportunities	Single	Published literature	Medical devices	Retrospective from database inception to 2015
				Literature search and patent search	Patents		
**Simpson S, 2018**	NIHR Horizon Scanning Research & Intelligence	Methods research paper	Early warning	Combination	Patients and the Public	All	Not specified

For peer review only

Authors	Organization/ Geography	Study Type	Main purpose	Methods used	Sources	Technology type	Horizon
	Centre (HSRIC)<br><br>(Birmingham, UK)			Horizon scanning and PPIE			
**Smith J, 2015**	NIHR Horizon Scanning Research & Intelligence Centre (HSRIC)<br><br>(Birmingham, UK)	Horizon scanning report	Technology assessment	Combination<br><br>Horizon scanning, consultation with clinical experts and PPIE	Patients and the Public<br><br>Experts	All	Not specified
**de Souza Antunes AM, 2012**	Instituto Nacional de Propriedade Industrial<br><br>(Brazil)	Methods research paper	Identify emerging trends	Single<br><br>Patent trend analysis	Patents	Nanotechnology applied to the screening, diagnosis, and treatment of disease	Retrospective<br><br>2000 to 2010
**Stafinski T, 2020**	Canadian Agency for Drugs and Technologies in Health (CADTH)<br><br>(Canada)	Methods research paper	Support decision making	Single<br><br>Expert consultation	Experts	All	Prospective<br><br>5 years ahead

For peer review only

Authors	Organization/ Geography	Study Type	Main purpose	Methods used	Sources	Technology type	Horizon
**Storz P, 2007**	German HTA in collaboration with the Federal Association of the Local Health Insurance Funds (AOK Bundesverband)<br>(Germany)	Horizon scanning report	Support decision making	Combination<br>Horizon Scanning; Literature searching	Published/ Unpublished literature<br>Internet<br>ZIM (private) database	Genetic testing	Not specified
**Takayoshi H, 2017**	VALUENEX Japan Inc<br>(Japan)	Methods research paper	Identify emerging trends	Single<br>Panoramic view analytics	Patents	Artificial intelligence applied to health and wellbeing	Retrospective<br>Published between 2001 and 2016
**Tal O, 2012**	Israeli Center for Technology Assessment in Health Care (ICTAHC)<br>(Israel)	Horizon Scanning methods paper	Technology assessment	Single<br>Horizon scanning using 3 time horizons	Experts<br>Industry<br>Published/ Unpublished literature	Medical devices	Prospective<br>Three horizons: short term 1 year; medium term and long-term

For peer review only

Authors	Organization/<br>Geography	Study Type	Main purpose	Methods used	Sources	Technology type	Horizon
					Internet<br>Clinical trials		
**Tark YJ, 2015**	Horizon Scanning Service of Innovative Global Health Technology (H-SIGHT)<br>(South Korea)	Horizon scanning report	Predict impact in healthcare services	Combination<br>Horizon scanning; Literature report; Clinical trials search	Published literature<br>Clinical trials	Medical device (polyurethane scaffolds)	Retrospective evidence search<br>Prospective prediction of impact (1 to 5 years) from launch
**Thorleuchter D, 2013**	Fraunhofer Institute for Technological Trend Analysis INT<br>(Germany)	Methods research paper	Identify emerging trends	Single<br>Web mining and latent semantic indexing	Internet	All	Not specified
**Trappey CV, 2014**	National Tsing Hua University and National	Methods research	Identify future developments	Combination	Patents	Dental implant connectors	Retrospective (published between 1992

For peer review only

Authors	Organization/<br>Geography	Study Type	Main purpose	Methods used	Sources	Technology type	Horizon
	Chiao Tung University<br>(Taiwan)	paper		Text mining and literature searches	Published clinical trials		and 2012)
**Trevitt S, 2016**	NIHR Horizon Scanning Research & Intelligence Centre (HSRIC)<br>(Birmingham, UK)	Horizon Scanning report	Early warning	Single	Published/<br>Unpublished literature	Wearable medical devices second and third generation Artificial Pancreas Device systems	Not specified
				Horizon scanning	Clinical trials		
					Internet; Internal databases; Third party databases; Manufacturer's websites		
**Vaganova EV, 2015**	National Research Tomsk State University<br>(Russia)	Conferen ce paper	Identify emerging trends	Single	Published literature	Medical devices and digital interventions (e-health and m-health, telemedicine and personalised smart devices)	Not specified
				Forecasting	Patents		

For peer review only

For peer review only - http://bmjopen.bmj.com/site/about/guidelines.xhtml

Authors	Organization/ Geography	Study Type	Main purpose	Methods used	Sources	Technology type	Horizon
**Varela-Lema L, 2012**	Galician Agency for Health Technology Assessment (avalia-t) (Spain)	Methods research paper	Identify emerging trends	Combination	Experts	Medical devices, procedures and diagnostics	Retrospective (4 to 5 years)
				Horizon scanning and expert consultation	Published/ Unpublished literature		
**Varela-Lema L, 2014**	Galician Agency for Health Technology Assessment (avalia-t) (Spain)	Methods research paper	Identify emerging trends	Single	Published literature	Medical devices, procedures and diagnostics	Prospective (1 to 2 years from availability)
				Bibliographic database search			
**Verbakel Jan Y, 2017**	National Institute for Health Research (NIHR) Diagnostic Evidence Co-operative Oxford at Oxford Health Foundation Trust (Oxford, UK)	Horizon scanning report	Identify evidence gaps	Single	Horizon scanning reports	Diagnostic tests	Not specified
				Content analysis			
**Wang J,**	National Chung	Methods	Identify	Single	Patents	Telehealth	Retrospective

Authors	Organization/ Geography	Study Type	Main purpose	Methods used	Sources	Technology type	Horizon
**2019**	Hsing University (Taiwan)	research paper	business opportunities	Patent trend analysis			(1991 to 2015)
**Wong Q W, 2020**	Singapore HS Organization (Singapore)	Methods research paper	Early warning	Single<br>Horizon Scanning	Commercial developer websites<br>Regulatory authorities (FDA and EMA)<br>Medical news media; Scientific journals; Conference proceedings<br>Reports from other horizon scanning organizations (CADTH, NIHR IO)<br>Trial registries	All (disruptive medicines, devices, digital and diagnostics)	Prospective (3 years to regulatory approval for drugs, different time horizon for DDD)
**Ye C, 2013**	Beijing City	Conferen	Identify future	Single	Nanotechnology	All (not	Not specified

For peer review only

Authors	Organization/ Geography	Study Type	Main purpose	Methods used	Sources	Technology type	Horizon
	University (China)	ce paper	developments	Technology roadmap	Roadmap of U.S. NASA	specified)	

For peer review only

Time horizons

Time horizons were not always described accurately nor was a rationale for the use of a given time limit provided. Some reports mentioned the time horizon as described in the reporting of the searches and often provided both an upper and a lower time limit for technology identification. The timeframes were variable and depended on the type of methods used and aim of the study. The longest timeframe analysed was 29 years and the shortest 18 months. Longer timeframes have been associated with patent mining or searching. Time horizons were used retrospectively or prospectively depending on the time endpoint and aim of the study. For example, retrospective searching was used to identify technologies in scope for established early warning systems[19] or to identify opportunities for future technology development by retrospective searching of patents and analysing results[32]. Prospective methods mostly involved consultation with experts such as clinicians, industry representatives and decision-makers for the prioritisation, prediction or validation of signals.[24,49,51]

Figure 4. Development of methods over a publication timeline

Ten studies published between 1999 and 2009 used HS, surveys, a Delphi study and internet searches or a combination of methods mainly consisting of literature searches, expert engagement via interviews and stakeholder surveys. A decade later the range of methods had exponentially increased, more authors use a combination of methods, and these are applied to different decision-making scenarios. Automated and machine learning approaches appear as early as 2012 and underpin almost a third of the methodologies reported.

Discussion

This review set out to study how foresight methodologies are used to identify innovative MedTech to support healthcare decision-making. To our knowledge, this review is the first to study the range of foresight methods alongside HS in the context of healthcare decision-making. This review also presents up-to-date information on the sources used for MedTech identification and intelligence analysis in this field. Despite the common understanding that MedTech reports are not usually disseminated by published

For peer review only

literature channels, published literature remains as one of the main sources used for MedTech identification which undermines the ability to discover innovation in a timely fashion.[10]

We discovered some interesting methods that laid outside the limits of the established HS techniques more traditionally defined and used by organisations operating in the health technology space. Methods such as a scientometric mapping approach using overlay mapping of three geographical, social and cognitive spaces[47] provide a visual approach to synthesize complex information and help strategic decision making. Panoramic view analytics[35] also exploit the benefits of visualisation to explore multi-dimensional technological information. Scenario-driven road-mapping[52], a hybrid method developed from traditional road-mapping and scenarios for technology foresight, overcomes some of the limitations of using these methods independently and provides a viable novel method particularly useful in forecasting technology adoption.

The timeframes used by the included studies revealed great variability and lacked justification. Considering the short life cycles of MedTech products, 18 to 24 months is an appropriate recommended timeframe. Automation techniques generally used for the identification of signals had been reported within the last decade. Patents were the most frequently used source for the exploration and testing of these techniques, often in combination with other sources such as clinical trial data and social media platforms such as Twitter or the Internet in general.

This review has unveiled the broad and vaguely reported use of the term 'horizon scanning' often used as an umbrella to describe the range of methodologies used for the identification of signals, which frequently involve searching in multiple sources and potentially consulting with experts, stakeholders or patients and the public. This inconsistency has been reported previously by Hines et al.[1] and most recently by Charlton and colleagues.[68] Given the important role that HS plays in health and care policy and decision-making, there is an urgent need for consistency and robust methods standardisation and reporting. We think that HS methods terminology and robust reporting standards will contribute to increase the precision in the identification of published HS literature. An internationally agreed methods guide could provide the groundwork for better standardisation and retrieval of HS reports. The International Horizon Scanning Initiative[69] and the International HealthTechScan[70] are two networks operating in this space that have the

For peer review only

potential to contribute towards greater methods standardisation and the Innovation Observatory has a seat in those two networks to influence and share in enhanced methods initiatives.

Finally, experts, broadly encompassing clinicians, patients and the public, were often used as a method to identify, prioritize or predict technology innovation, as well as a source to compare signals identified by other means. Expert engagement methods were not always specified nor were reported the type of experts (patients, public, and professionals) or their professional backgrounds (clinicians, entrepreneurs, regulators, consultancy companies, industry). There is evidence that suggests expert engagement alone can bias predictions[18] therefore it would be advisable to practice transparency in the reporting of expert selection and engagement methods.

The use of a systematic literature search and reporting methods to identify published and unpublished literature allowed for a robust and reproducible search in a broad range of databases beyond the traditional medical databases. Further, we believe that this is the first literature review that aims to discover foresight and HS methods for MedTech innovations to support decision-making. The analysis of objectives, methods and sources together may contribute to answer some of the epistemological questions around which foresight method to use to detect future MedTech development relevant to healthcare decision-makers.[6] This review has some limitations. Firstly, the search was geared towards identifying several 'known' methods and that may have limited the possibility of identifying new or unknown methods. However, as already demonstrated, new methods were identified possibly due to the broad inclusion criteria. Secondly, screening was undertaken mostly by one author with the support of an automated screening software and a second checker. Although some random sampling for screening was undertaken, some potentially valid records could have been missed due to poor indexing or lack of abstract. Concept visualisation of those records could have been used to identify further potentially relevant records; however, due to time and resource constrains this option was not explored. Lastly, the last search was undertaken in 2021 and no further searches have been run since then. Although it is possible that new methods have been published since, they are unlikely to change the conclusions of this review.

Conclusion

For peer review only

In the healthcare field there is global interest in understanding the MedTech innovation pipeline, with a view on system preparedness and acceleration of technology in areas of unmet need. Exploiting HS and foresight methods will support advanced signalling and system preparedness to harness the opportunities posed by innovative MedTech, but also allow us time to address some of the future challenges ahead of those technologies being placed on the market. This review has revealed that there is need for a flexible approach to methods development, the combination of existing methods[52] or the application of modern computing and artificial intelligence solutions to deal with vast amounts of data,[35] are potential solutions to the current methods limitations. Most importantly, this review clearly highlighted a scientific hindrance due to the lack of methods standardisation and reporting.

Studying impact of HS intelligence in the decision-making process was out of scope of this review, nevertheless, is an important future research need. This review has provided the foundation work for the next steps in the programme of the National Institute for Health and Care Research Innovation Observatory[71] work which will focus on the development of agreed standards for reporting methods and the development of HS methods guides.

Box 1. Recommendations for future methods standardisation

1. Uniformly use of terminology for the description of methods
2. Robust reporting of sources searched, and strategies used
3. Rationale for the use of time limits
4. Practice transparency when reporting the background of experts involved
5. Report functionality of automated methods when used

Author Statement:

Sonia G Gonzalez-Moral was responsible for the conceptualization of this project, methodology, writing of the original draft, visualization of results and the overall project administration. Fiona R Beyer, Anne O Oyewole and Luke Wainwright were involved in the revising and editing of this review and the supervision of this work. Catherine Richmond was involved in the validation of the search strategy, record

For peer review only

326 sifting and writing of original draft. Dawn Craig was responsible for writing and editing of this review,
327 supervision of this work and funding acquisition.

328 "All authors have completed the ICMJE uniform disclosure form at http://www.icmje.org/disclosure-of-
329 interest/ and declare: no support from any organisation for the submitted work; no financial relationships
330 with any organisations that might have an interest in the submitted work in the previous three years; no
331 other relationships or activities that could appear to have influenced the submitted work."

332

333

334 **References**

1. Hines P, Hiu Yu L, Guy RH, et al. Scanning the horizon: a systematic literature review of methodologies. *BMJ Open* 2019;9(5):e026764-e64. doi: 10.1136/bmjopen-2018-026764
2. Amanatidou E, Butter M, Carabias V, et al. On concepts and methods in horizon scanning: Lessons from initiating policy dialogues on emerging issues. *Science and Public Policy* 2012;39(2):208-21. doi: doi.org/10.1093/scipol/scs017
3. National Health Service (NHS). The NHS Long Term Plan: NHS 2019.
4. HM Government. Life Sciences Vision, 2021.
5. MedTech Europe. MedTech Europe: About the Industry 2020 [Available from: https://www.medtecheurope.org/about-the-industry/].
6. Bauer W, Schimpf S. Back to the Future: Scoping Dynamics in Industrial Innovation. *IEEE Engineering Management Review* 2020;48(2):72-82.
7. Fuchs S, Olberg B, Panteli D, et al. HTA of medical devices: Challenges and ideas for the future from a European perspective. *Health Policy* 2017;121(3):215-29. doi: 10.1016/j.healthpol.2016.08.010
8. U.S. Food and Drugs Administration. FDA Medical Device Databases 2021 [Available from: https://www.fda.gov/medical-devices/device-advice-comprehensive-regulatory-assistance/medical-device-databases].
9. European Commission. EUDAMED - European Database on Medical Devices [Available from: https://ec.europa.eu/tools/eudamed/#/screen/home; accessed 22 January 2023].
10. Farrah K, Mierzwinski-Urban M. Almost half of references in reports on new and emerging nondrug health technologies are grey literature. *J Med Libr Assoc* 2019;107(1):43-48. doi: doi.org/10.5195/jmla.2019.539
11. Cuhls KE. Horizon Scanning in Foresight – Why Horizon Scanning is only a part of the game. *Futures & Foresight Science* 2020;2(1):e23. doi: 10.1002/ffo2.23
12. Doos L, Packer C, Ward D, et al. Past speculations of the future: a review of the methods used for forecasting emerging health technologies. *BMJ Open* 2016;6(3):e010479. doi: 10.1136/bmjopen-2015-010479
13. White H, Albers B, Gaarder M, et al. Guidance for producing a Campbell evidence and gap map. *Campbell Systematic Reviews* 2020;16(4):e1125. doi: 10.1002/cl2.1125
14. Rethlefsen ML, Kirtley S, Waffenschmidt S, et al. PRISMA-S: an extension to the PRISMA Statement for Reporting Literature Searches in Systematic Reviews. *Syst Rev* 2021;10(1):39. doi: 10.1186/s13643-020-01542-z PMCID: PMC7839230. [published Online First: 2021/01/28]
15. McGowan J, Sampson M, Salzwedel DM, et al. PRESS Peer Review of Electronic Search Strategies: 2015 Guideline Statement. *Journal of Clinical Epidemiology* 2016;75:40-46. doi: 10.1016/j.jclinepi.2016.01.021
16. The Open University. CORE [updated 27/02/2023. Available from: https://core.ac.uk/].
17. Ouzzani M, Hammady H, Fedorowicz Z, et al. Rayyan-a web and mobile app for systematic reviews. *Syst Rev* 2016;5(1):210. doi: 10.1186/s13643-016-0384-4 PMCID: PMC5139140. [published Online First: 2016/12/07]

For peer review only

18. Apreda R, Bonaccorsi A, dell'Orletta F, et al. Expert forecast and realized outcomes in technology foresight. *Technological Forecasting and Social Change* 2019;141:277-88. doi: 10.1016/j.techfore.2018.12.006
19. Dixon LC, Ward DJ, Smith J, et al. New and emerging technologies for the diagnosis and monitoring of chronic obstructive pulmonary disease. *Chronic Respiratory Disease* 2016;13(4):321-36. doi: 10.1177/1479972316636994
20. Douw K, Vondeling H, Eskildsen D, et al. Use of the internet in scanning the horizon for new and emerging health technologies: A survey of agencies involved in horizon scanning. *Journal of Medical Internet Research* 2003;5(1):33-45. doi: 10.2196/jmir.5.1.e6
21. Lerner JC, Robertson DC, Goldstein SM. Case studies on forecasting for innovative technologies: Frequent revisions improve accuracy. *Health Affairs* 2015;34(2):311-18. doi: 10.1377/hlthaff.2014.1066 PMCID: 25646112
22. Migliore A, Perrini MR, Jefferson T, et al. Implementing a national early awareness and alert system for new and emerging health technologies in Italy: The COTE project. *International Journal of Technology Assessment in Health Care* 2012;28(3):321-26. doi: 10.1017/S0266462312000384
23. Morrison A. Scanning the horizon in a decentralized healthcare system: The Canadian experience. *International Journal of Technology Assessment in Health Care* 2012;28(3):327-32. doi: 10.1017/S0266462312000323
24. Mundy L, Merlin TL, Parrella A, et al. The Australia and New Zealand horizon scanning network. *Australian health review : a publication of the Australian Hospital Association* 2005;29(4):395-97. doi: 10.1071/AH050395
25. O'Malley SP, Jordan E. Horizon scanning of new and emerging medical technology in Australia: Its relevance to medical services advisory committee health technology assessments and public funding. *International Journal of Technology Assessment in Health Care* 2009;25(3):374-82. doi: 10.1017/S0266462309990031
26. Robert G, Stevens A, Gabbay J. 'Early warning systems' for identifying new healthcare technologies. *Health Technology Assessment* 1999;3(13):iii-99. doi: 10.3310/hta3130 PMCID: 10632623
27. Simpson S, Cook A, Miles K. Patient and public involvement in early awareness and alert activities: An example from the United Kingdom. *International Journal of Technology Assessment in Health Care* 2018;34(1):10-17. doi: 10.1017/S0266462317004421
28. Trevitt S, Simpson S, Wood A. Artificial Pancreas Device Systems for the Closed-Loop Control of Type 1 Diabetes: What Systems Are in Development? *Journal of Diabetes Science and Technology* 2016;10(3):714-23. doi: 10.1177/1932296815617968
29. Wong WQ, Lin L, Ju H, et al. Towards greater impact in health technology assessment: Horizon scanning for new and emerging technologies in Singapore. *International Journal of Technology Assessment in Health Care* 2020;36(4):304-10. doi: 10.1017/S0266462320000343
30. Baskici C, Atan S, Ercil Y. Forecasting of innovation in the light of semantic networks. 3rd World Conference on Technology, Innovation and Entrepreneurship, WOCTINE 2019, June 21, 2019 - June 23, 2019. Istanbul, Turkey: Elsevier B.V., 2019:443-49.
31. Bhavnani SP, Parakh K, Atreja A, et al. 2017 Roadmap for Innovation-ACC Health Policy Statement on Healthcare Transformation in the Era of Digital Health, Big Data, and Precision Health: A Report of the American College of Cardiology Task Force on Health Policy Statements and Systems of Care. *Journal of the American College of Cardiology* 2017;70(21):2696-718. doi: 10.1016/j.jacc.2017.10.018
32. Chang SWC, Trappey CV, Trappey AJC, et al. Forecasting dental implant technologies using patent analysis. 2014 Portland International Center for Management of Engineering and Technology, PICMET 2014, July 27, 2014 - July 31, 2014. Kanazawa, Japan: Institute of Electrical and Electronics Engineers Inc., 2014:1483-91.
33. de Souza Antunes AM, de Menezes Alencar MS, da Silva CH, et al. Trends in nanotechnology patents applied to the health sector. *Recent Patents on Nanotechnology* 2012;6(1):29-43. doi: 10.2174/187221012798109309
34. Gelderblom GJ, De Wilt M, Cremers G, et al. Rehabilitation robotics in robotics for healthcare; a roadmap study for the European Commission. 2009 IEEE International Conference on Rehabilitation Robotics, ICORR 2009, June 23, 2009 - June 26, 2009. Kyoto, Japan: IEEE Computer Society, 2009:834-38.
35. Hayashi T. R&D trend analysis of wellbeing AI using panoramic view analytics. 2017 AAAI Spring Symposium, March 27, 2017 - March 29, 2017. Stanford, CA, United states: AI Access Foundation, 2017:673-74.

For peer review only

36. Mühlroth C, Grottke M. Artificial Intelligence in Innovation: How to Spot Emerging Trends and Technologies. *IEEE Transactions on Engineering Management* 2020;PP(99):Jan-18. doi: 10.1109/TEM.2020.2989214

37. Sheikh NJ, Sheikh O. Forecasting of biosensor technologies for emerging point of care and medical IoT applications using bibliometrics and patent analysis. 2016 Portland International Conference on Management of Engineering and Technology, PICMET 2016, September 4, 2016 - September 8, 2016. Honolulu, HI, United states: Institute of Electrical and Electronics Engineers Inc., 2016:3082-93.

38. Thorleuchter D, Van Den Poel D. Weak signal identification with semantic web mining. *Expert Systems with Applications* 2013;40(12):4978-85. doi: 10.1016/j.eswa.2013.03.002

39. Vaganova EV, Syryamkin MV. Forecasting of the development of professional medical equipment engineering based on neuro-fuzzy algorithms. 5th International Scientific Conference on New Operational Technologies, NEWOT 2015, 2015.

40. Varela-Lema L, De La Fuente-Cid R, Lopez-Garcia M. Developing a prioritized list of innovative technologies: the Spanish experience. *International Journal of Technology Assessment in Health Care* 2014;30(6):626-33. doi: 10.1017/S0266462314000774

41. Varela-Lema L, Punal-Rioboo J, Accion BC, et al. Making processes reliable: A validated pubmed search strategy for identifying new or emerging technologies. *International Journal of Technology Assessment in Health Care* 2012;28(4):452-59. doi: 10.1017/S0266462312000578

42. Brown IT, Smale A, Verma A, et al. Medical technology horizon scanning. *Australasian Physical and Engineering Sciences in Medicine* 2005;28(3):200-03. doi: 10.1007/BF03178717 PMCID: 16250477

43. Chalkidou A, Erskine J, Macmillan T, et al. Factors affecting horizon scanning for hospital-based health technology assessment. 16th HTAi's Annual Meeting. Germany: International Journal of Technology Assessment in Health Care, 2019:25-26.

44. Ibargoyen-Roteta N, Gutierrez-Ibarluzea I, Asua J, et al. Scanning the horizon of obsolete technologies: Possible sources for their identification. *International Journal of Technology Assessment in Health Care* 2009;25(3):249-54. doi: 10.1017/S0266462309990249 PMCID: 19619342

45. Itoh S, Kano S. Technology Forecasting for Medical Devices Guidance Formulation: A Case Study in Japan. *Therapeutic Innovation and Regulatory Science* 2019;53(4):481-89. doi: 10.1177/2168479018793370 PMCID: 30175593

46. Postma TJBM, Alers JC, Terpstra S, et al. Medical technology decisions in The Netherlands: How to solve the dilemma of technology foresight versus market research? *Technological Forecasting and Social Change* 2007;74(9):1823-33. doi: 10.1016/j.techfore.2007.05.011

47. Rotolo D, Rafols I, Hopkins MM, et al. Strategic intelligence on emerging technologies: Scientometric overlay mapping. *Journal of the Association for Information Science and Technology* 2017;68(1):214-33. doi: 10.1002/asi.23631

48. Ruggeri M, Cadeddu C, Roazzi P, et al. Multi–criteria–decision–analysis (MCDA) for the horizon scanning of health innovations an application to COVID 19 emergency. *International Journal of Environmental Research and Public Health* 2020;17(21):1-9. doi: 10.3390/ijerph17217823 PMCID: 33114587

49. Stafinski T, Topfer LA, Zakariasen K, et al. The role of surgeons in identifying emerging technologies for health technology assessment. *Canadian Journal of Surgery* 2010;53(2):86-92. PMCID: PMC2845954

50. Storz P, Kolpatzik K, Perleth M, et al. Future relevance of genetic testing: A systematic horizon scanning analysis. *International Journal of Technology Assessment in Health Care* 2007;23(4):495-504. doi: 10.1017/S0266462307070596

51. Flick C, Zamani ED, Stahl BC, et al. The future of ICT for health and ageing: Unveiling ethical and social issues through horizon scanning foresight. *Technological Forecasting and Social Change* 2020;155 doi: 10.1016/j.techfore.2020.119995

52. Hussain M, Tapinos E, Knight L. Scenario-driven roadmapping for technology foresight. *Technological Forecasting and Social Change* 2017;124:160-77. doi: 10.1016/j.techfore.2017.05.005

53. Ouyang Z-L, Fan Y-B, Zhou P, et al. Identification of hotspot technologies in the field of radiation diagnosis based on bibliometric analysis of global patents. 2014 International Conference on Information Science, Electronics and Electrical Engineering, ISEEE 2014, April 26, 2014 - April 28, 2014. Sapporo City, Hokkaido, Japan: Institute of Electrical and Electronics Engineers Inc., 2014:84-88.

54. Trappey CV, Trappey AJC, Peng H-Y, et al. A knowledge centric methodology for dental implant technology assessment using ontology based patent analysis and clinical meta-analysis. *Advanced Engineering Informatics* 2014;28(2):153-65. doi: 10.1016/j.aei.2014.03.001

55. Ye C, Feng L. Future-oriented technology analysis of technology roadmap based on text mining. 2013 10th International Conference on Fuzzy Systems and Knowledge Discovery (FSKD), 2013:1126-30.

For peer review only - http://bmjopen.bmj.com/site/about/guidelines.xhtml

56. Handa N, Mochizuki S, Fujiwara Y, et al. Future development of artificial organs related with cutting edge emerging technology and their regulatory assessment: PMDA's perspective. *Journal of Artificial Organs* 2020;23(3):203-06. doi: 10.1007/s10047-020-01161-4 PMCID: 32112156
57. Packer C. The National Horizon Scanning Centre (NHSC): Early warning for new and emerging health technologies in England. *Evidence-Based Healthcare and Public Health* 2005;9(6):410-13. doi: 10.1016/j.ehbc.2005.09.005
58. Smith J, Ward D, Michaelides M, et al. New and emerging technologies for the treatment of inherited retinal diseases: A horizon scanning review. *Eye (Basingstoke)* 2015;29(9):1131-40. doi: 10.1038/eye.2015.115 PMCID: 26113499
59. Tal O, Hakak N. Early awareness and alert systems for medical technologies in Israel. *International Journal of Technology Assessment in Health Care* 2012;28(3):333-38. doi: 10.1017/S0266462312000396
60. Lee J, Kim J, Hong YJ, et al. Health information technology trends in social media: Using twitter data. *Healthcare Informatics Research* 2019;25(2):99-105. doi: 10.4258/hir.2019.25.2.99
61. Shen Y-C, Wang M-Y, Yang Y-C. Discovering the potential opportunities of scientific advancement and technological innovation: A case study of smart health monitoring technology. *Technological Forecasting and Social Change* 2020;160 doi: 10.1016/j.techfore.2020.120225
62. Wang J, Chen Y-J. A novelty detection patent mining approach for analyzing technological opportunities. *Advanced Engineering Informatics* 2019;42 doi: 10.1016/j.aei.2019.100941
63. Tark JY, Jeong J-Y, Lee M, et al. Early assessment and prediction of potential impact of the implantation of polyurethane scaffolf in partial meniscal lesions: a pilot horizon scanning activity in South Korea. *International Journal of Technology Assessment in Health Care* 2015;31(6):380-89. doi: 10.1017/S0266462315000689
64. Shekelle PG, Goldman DP. Development of a Group Judgment Process for Forecasts of Health Care Innovations. *JAMA network open* 2018;1(7):e185108. doi: 10.1001/jamanetworkopen.2018.5108 PMCID: 30646380
65. Pretorius L, Winzker DH. Biomedical technology: A case study of forecasting in pulsed electro magnetic field therapy. PICMET '10 - Portland International Center for Management of Engineering and Technology, Proceedings - Technology Management for Global Economic Growth: IEEE Computer Society, 2010:248-53.
66. Verbakel JY, Turner PJ, Thompson MJ, et al. Common evidence gaps in point-of-care diagnostic test evaluation: a review of horizon scan reports. *BMJ Open* 2017;7(9):e015760. doi: https://dx.doi.org/10.1136/bmjopen-2016-015760
67. Hospodkova P, Bartak M, Vacíková K, et al. Global centers of medical device technology: United States, Europe and China. *Lekar a Technika* 2018;48(4):136-44.
68. Charlton P, Kean T, Liu RH, et al. Use of environmental scans in health services delivery research: a scoping review. *BMJ Open* 2021;11(11):e050284. doi: 10.1136/bmjopen-2021-050284
69. International Horizon Scanning Initiative. Horizon Scanning System 2023 [Available from: https://ihsi-health.org/horizon-scanning-system/; accessed 20/02/2023].
70. EuroScan International Network. International HealthTechScan (i-HTS) 2022 [Available from: https://www.i-hts.org/network; accessed 20/02/2023].
71. National Institute for Health and Care Research Innovation Observatory (NIHRIO). 2022 [Available from: https://www.io.nihr.ac.uk/; accessed 12/12/2022].

Figure 1. Aims and objectives for using HS/foresight methods in MedTech innovation identification

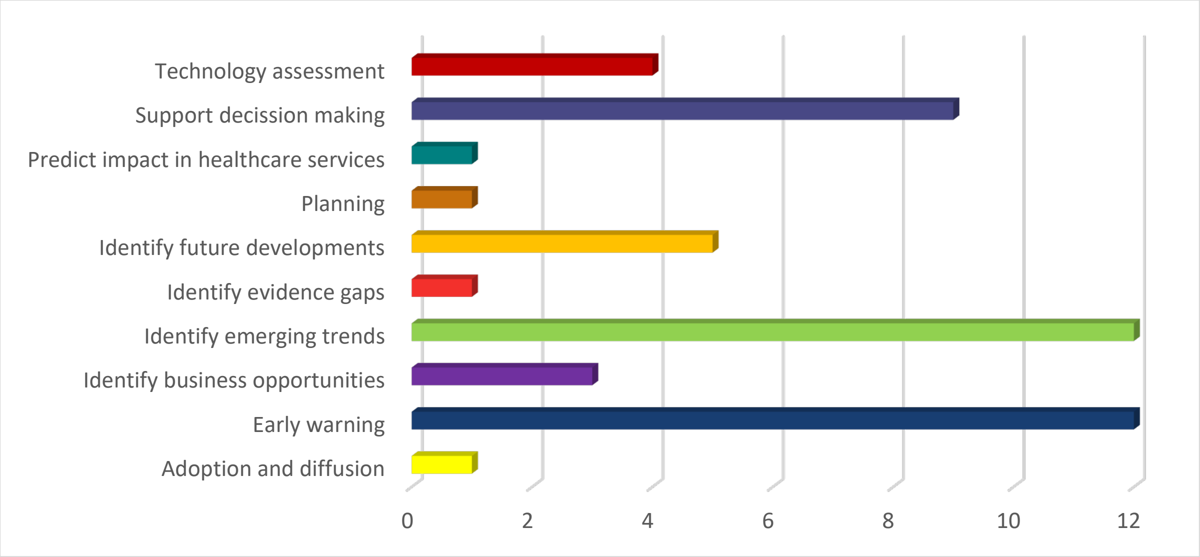

peer review only

Figure 2. Methods and sources overlap

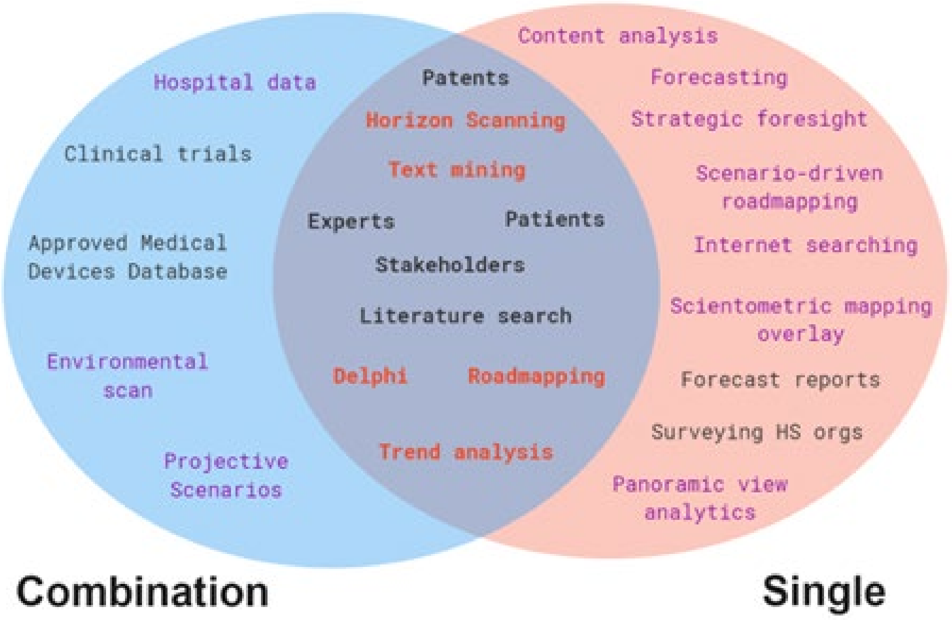

Black denotes sources; Red denotes methods used by single or combination studies; Purple denotes methods only appearing once in any study (combination or single)

review only

Figure 3. Type of sources used as described in the included reports

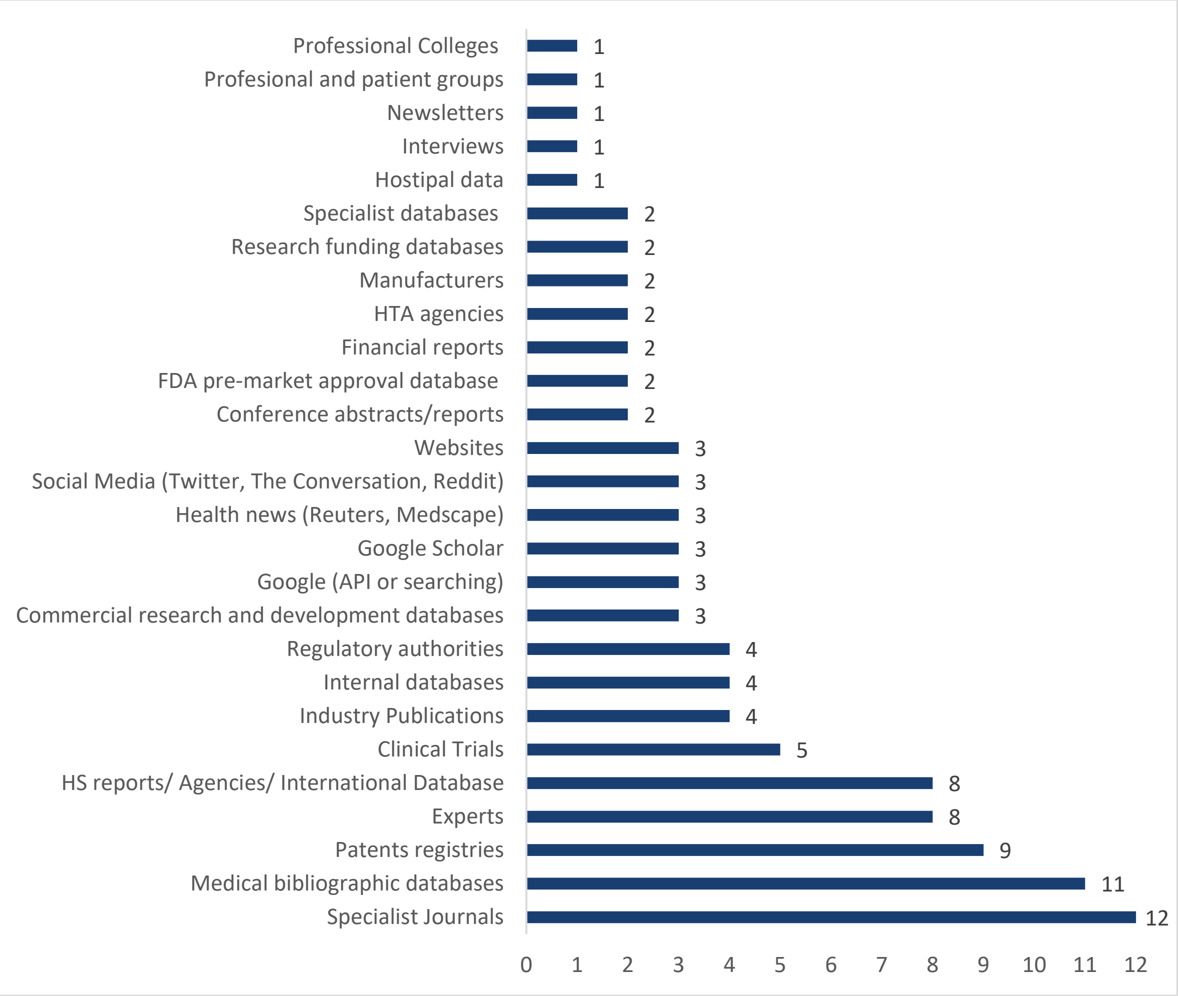

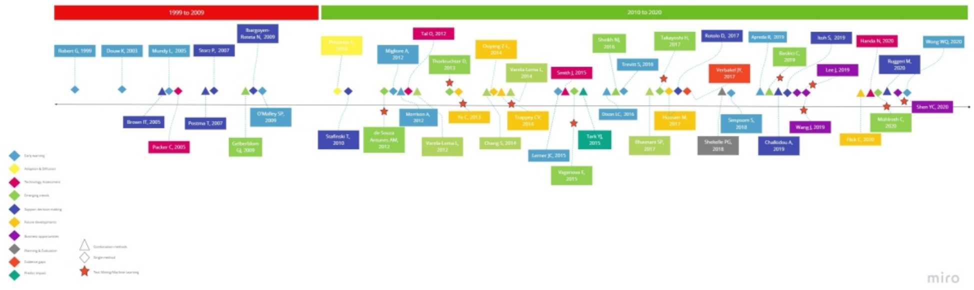

Figure 4. Development of methods over a publication timeline

885x316mm (72 x 72 DPI)

Appendix A

Table A.1. PRISMA-S Checklist

Section/topic	#	Checklist item	Location(s) Reported
**INFORMATION SOURCES AND METHODS**			
Database name	1	Name each individual database searched, stating the platform for each.	Pg. 4-5 and Appendix B
Multi-database searching	2	If databases were searched simultaneously on a single platform, state the name of the platform, listing all of the databases searched.	N/A
Study registries	3	List any study registries searched.	N/A
Online resources and browsing	4	Describe any online or print source purposefully searched or browsed (e.g., tables of contents, print conference proceedings, web sites), and how this was done.	Pg. 4-5 and Appendix B
Citation searching	5	Indicate whether cited references or citing references were examined, and describe any methods used for locating cited/citing references (e.g., browsing reference lists, using a citation index, setting up email alerts for references citing included studies).	Pg. 4-5
Contacts	6	Indicate whether additional studies or data were sought by contacting authors, experts, manufacturers, or others.	N/A
Other methods	7	Describe any additional information sources or search methods used.	Pg. 4-5 and Appendix B
**SEARCH STRATEGIES**			
Full search strategies	8	Include the search strategies for each database and information source, copied and pasted exactly as run.	Appendix B
Limits and restrictions	9	Specify that no limits were used, or describe any limits or restrictions applied to a search (e.g., date or time period, language, study design) and provide justification for their use.	Pg. 4-5
Search filters	10	Indicate whether published search filters were used (as originally designed or modified), and if so, cite the filter(s) used.	N/A

For peer review only

Prior work	11	Indicate when search strategies from other literature reviews were adapted or reused for a substantive part or all of the search, citing the previous review(s).	N/A
Updates	12	Report the methods used to update the search(es) (e.g., rerunning searches, email alerts).	N/A
Dates of searches	13	For each search strategy, provide the date when the last search occurred.	Appendix B
**PEER REVIEW**			
Peer review	14	Describe any search peer review process.	Pg. 4-5
**MANAGING RECORDS**			
Total Records	15	Document the total number of records identified from each database and other information sources.	Appendix B
Deduplication	16	Describe the processes and any software used to deduplicate records from multiple database searches and other information sources.	Pgs. 4-5

PRISMA-S: An Extension to the PRISMA Statement for Reporting Literature Searches in Systematic Reviews
Rethlefsen ML, Kirtley S, Waffenschmidt S, Ayala AP, Moher D, Page MJ, Koffel JB, PRISMA-S Group.
Last updated February 27, 2020.

For peer review only

Appendix B

Figure B.1. MEDLINE search strategy

1	Forecasting/
2	(horizon scan or horizon scanning).ti,ab,kf,tw.
3	(environmental scan or environmental scanning).ti,ab,kf,tw.
4	(foresight or forecasting).ti,ab,kf,tw.
5	("Early awareness" and "alert systems").ti,ab,kf,tw.
6	(readiness adj3 preparedness).ti,ab,kf,tw.
7	(monitor* adj6 ("health technolog*" or "medical technolog*")).ti,ab,kf,tw.
8	("road map" or roadmap).ti,ab,kf,tw.
9	patent analys*s.ti,ab,kf,tw.
10	bibliometric analys*s.ti,ab,kf,tw.
11	trend* analys*s.ti,ab,kf,tw.
12	backcasting.ti,ab,kf,tw.
13	delphi.ti,ab,kf,tw.
14	*delphi technique/
15	or/1-14
16	biomedical technology/
17	exp technology assessment, biomedical/
18	((new or innovat* or emerg* or early) adj6 ("health technolog*" or "medical technolog*")).ti,ab.
19	or/16-18
20	15 and 19

Databases searched

Medline (OVID)

Database: Ovid MEDLINE(R) and Epub Ahead of Print, In-Process, In-Data-Review & Other Non-Indexed Citations, Daily and Versions(R) <1946 to March 25, 2021>

Date: 26/03/2021

Search results: 1003

Search Strategy:

----

1 Forecasting/ (87084)

2 (horizon scan or horizon scanning).ti,ab,kf,tw. (247)

3 (environmental scan or environmental scanning).ti,ab,kf,tw. (1780)

4 (foresight or forecasting).ti,ab,kf,tw. (10290)

5 ("Early awareness" and "alert systems").ti,ab,kf,tw. (9)

6 (readiness adj3 preparedness).ti,ab,kf,tw. (203)

7 (monitor* adj6 ("health technolog*" or "medical technolog*")).ti,ab,kf,tw. (93)

8 ("road map" or roadmap).ti,ab,kf,tw. (7122)

For peer review only

9 patent analys*s.ti,ab,kf,tw. (75)

10 bibliometric analys*s.ti,ab,kf,tw. (2302)

11 trend* analys*s.ti,ab,kf,tw. (5160)

12 backcasting.ti,ab,kf,tw. (29)

13 delphi.ti,ab,kf,tw. (12256)

14 *delphi technique/ (1233)

15 or/1-14 (123740)

16 biomedical technology/ (6719)

17 exp technology assessment, biomedical/ (11322)

18 ((new or innovat* or emerg* or early) adj6 ("health technolog*" or "medical technolog*")).ti,ab. (1640)

19 or/16-18 (18729)

20 15 and 19 (1003)

For peer review only

2. Embase

Database: Embase <1974 to 2021 March 25>

Date: 26/03/2021

Search results: 1295

Search Strategy:

----

1 forecasting/ (46958)

2 (horizon scan or horizon scanning).ti,ab,kw. (341)

3 (environmental scan or environmental scanning).ti,ab,kw. (2175)

4 (foresight or forecasting).ti,ab,kw. (12472)

5 (readiness adj3 preparedness).ti,ab,kw. (218)

6 (monitor* adj6 ("health technolog*" or "medical technolog*")).ti,ab,kw. (142)

7 ("road map" or roadmap).ti,ab,kw. (8918)

8 patent analys*s.ti,ab,kw. (181)

9 bibliometric analys*s.ti,ab,kw. (2705)

10 trend* analys*s.ti,ab,kw. (7382)

11 backcasting.ti,ab,kw. (31)

12 delphi.ti,ab,kw. (16900)

13 *delphi study/ (2074)

14 *bibliometrics/ (3371)

15 *trend study/ (865)

16 or/1-15 (95349)

17 medical technology/ (34204)

18 biomedical technology assessment/ (15008)

19 ((new or innovat* or emerg* or early) adj6 ("health technolog*" or "medical technolog*")).ti,ab. (2248)

20 or/17-19 (49615)

21 16 and 20 (1295)

3. IEEE Xplore

Database URL: https://ieeexplore-ieee-org.libproxy.ncl.ac.uk/search

Search Date: 05032021

Search strategy: database does not allow to combine search strings or save searches. Searched string by string and assessed relevance of results on screen by title and abstract. Those results judged relevant as first screening have been downloaded for further assessment.

No time limit, No language limits

Showing 1-4 of 4 for ("Document Title":"horizon scan" OR "Abstract":"horizon scan" OR "Index Terms":"horizon scan") – search retrieved no relevant results

Showing 1-25 of 437 for ("Publication Title":"foresight" OR "Abstract":"foresight" OR "Index Terms":"foresight") – all 437 results downloaded for further assessment

Conferences (352)

Journals (50)

Magazines (21)

Early Access Articles (12)

Books (1)

Courses (1)

Showing 1-25 of 71 for (("Document Title":"forecast" OR "Abstract":"forecast" OR "Index Terms":"forecast") AND "Document Title":"health") – all 71 results downloaded for further assessment

Conferences (59)

Journals (11)

Magazines (1)

Showing 1-19 of 19 for (("Document Title":"roadmap" OR "Abstract":"roadmap" OR "Index Terms":"roadmap") AND "Document Title":"health") – search retrieved no relevant results

For peer review only

Showing 1-17 of 17 for (("Document Title":"patent" OR "Document Title":"bibliometric" OR "Document Title":"trend" OR "Document Title":"backcasting" OR "Document Title":"delphi") AND "Document Title":"health") – 1 reference downloaded for further assessment/all references downloaded for further assessment.

4. Compendex – Engineering Village (Elsevier)

Database URL: https://www.engineeringvillage.com/search/quick.url

Search date: 26/03/2020

Search strategy:

((((((environmental scanning NEAR/6 health) OR (horizon scanning NEAR/6 health) OR (Forecasting NEAR/6 health) OR (Foresight NEAR/06 health) OR (Roadmap NEAR/06 health) OR (patent analys*s NEAR/06 health) OR (bibliometric analys*s NEAR/06 health) OR (trend analys*s NEAR/06 health) OR (backcasting NEAR/06 health) OR (delphi NEAR/06 health)) WN KY)) NOT ({cp} WN DT)) NOT (({ch} OR {bk} OR {er}) WN DT))

Removed conference proceedings, books and book chapters:

Retrieved results: 3,465

5. Scopus

Advanced search interface

Database URL: https://www.scopus.com/search/form.uri?display=advanced

Search date: 09/03/2021

Retrieved results: 2,396

Search strategy:

(TITLE-ABS-KEY ("horizon scanning") OR TITLE-ABS-KEY (forecasting) OR TITLE-ABS-KEY ("road map" OR roadmap) OR TITLE-ABS-KEY (patent AND anlys*s) OR TITLE-ABS-KEY (bibliometric AND analys*s) OR TITLE-ABS-KEY (trend AND analys*s) OR TITLE-ABS-KEY (backcasting) OR TITLE-ABS-KEY (delphi)) AND (TITLE-ABS-KEY ("medical technology") OR TITLE-ABS-KEY ("health technology"))

6. Web of Science CORE Collection

Advanced search interface

Database URL:
https://apps.webofknowledge.com/WOS_AdvancedSearch_input.do?product=WOS&search_mode=AdvancedSearch&SID=D5cLI4VsqgVb6Yc6hBB&locale=en_US

Date searched 09/03/2021

Retrieved results: 214 all downloaded for further assessment

Search strategy:

For peer review only

Set	Results	Save History / Create AlertOpen Saved History
# 12	214	#11 AND #8<br>*Indexes=SCI-EXPANDED, SSCI, A&HCI, CPCI-S, CPCI-SSH, ESCI Timespan=All years*
# 11	11,875	#10 OR #9<br>*Indexes=SCI-EXPANDED, SSCI, A&HCI, CPCI-S, CPCI-SSH, ESCI Timespan=All years*
# 10	7,049	TI=("health technology" OR "health technologies") OR AB=("health technology" OR "health technologies")<br>*Indexes=SCI-EXPANDED, SSCI, A&HCI, CPCI-S, CPCI-SSH, ESCI Timespan=All years*
# 9	4,942	TI=("medical technology" OR "medical technologies") OR AB=("medical technolo gy" OR "medical technologies")<br>*Indexes=SCI-EXPANDED, SSCI, A&HCI, CPCI-S, CPCI-SSH, ESCI Timespan=All years*
# 8	236,408	#7 OR #6 OR #5 OR #4 OR #3 OR #2 OR #1<br>*Indexes=SCI-EXPANDED, SSCI, A&HCI, CPCI-S, CPCI-SSH, ESCI Timespan=All years*
# 7	19,579	TI=(delphi) OR AB=(delphi)<br>*Indexes=SCI-EXPANDED, SSCI, A&HCI, CPCI-S, CPCI-SSH, ESCI Timespan=All years*
# 6	345	TI=(backcasting) OR AB=(backcasting)<br>*Indexes=SCI-EXPANDED, SSCI, A&HCI, CPCI-S, CPCI-SSH, ESCI Timespan=All years*
# 5	10,036	TI=("trend analysis" OR "trend analyses") OR AB=("trend analysis" OR "trend an alyses")<br>*Indexes=SCI-EXPANDED, SSCI, A&HCI, CPCI-S, CPCI-SSH, ESCI Timespan=All years*
# 4	5,608	TI=("bibliometric analysis" OR "bibliometric analyses") OR AB=("bibliometric ana lysis" OR "bibliometric analyses")<br>*Indexes=SCI-EXPANDED, SSCI, A&HCI, CPCI-S, CPCI-SSH, ESCI Timespan=All years*
# 3	749	TI=("patent analysis" OR "patent analyses") OR AB=("patent analysis" OR "paten t analyses")<br>*Indexes=SCI-EXPANDED, SSCI, A&HCI, CPCI-S, CPCI-SSH, ESCI Timespan=All years*
# 2	21,200	TI=("road map" OR roadmap) OR AB=("road map" OR roadmap)<br>*Indexes=SCI-EXPANDED, SSCI, A&HCI, CPCI-S, CPCI-SSH, ESCI Timespan=All years*
# 1	180,250	TI=(horizon scanning OR forecasting) OR AB=(horizon scanning OR forecasting)

For peer review only

Indexes=SCI-EXPANDED, SSCI, A&HCI, CPCI-S, CPCI-SSH, ESCI Timespan=All years

7. International HTA Database

URL: https://database.inahta.org/search/advanced

Date searched 04/02/2022

Retrieved results: 334

Search strategy:

(forecast*)[Title] OR (horizon scan*)[Title] OR (environmental scan*)[Title] OR (foresight)[Title] OR ("early awareness" AND "alert systems")[Title] OR (roadmap* OR "road map*")[Title] OR ("bibliometric analys*")[Title] OR (backcasting)[Title] OR ("trend analys*")[Title] OR (delphi) AND ((new OR innovative OR early OR emerging))

Retrieved 334. Sifted on screen. 35 results downloaded for further assessment. Included in lit review: 0

Grey literature searches:

Platform: Google Chrome

Search strategy:

horizon scanning filetype:pdf

Date: 26/02/2021

Results: limited to 2020 onwards, checked the first 5 pages of results.

Included for further analysis:

https://www.pcori.org/sites/default/files/PCORI-Health-Care-COVID-19-Horizon-Scanning-System-Supplement-High-Impact-Report-September-2020.pdf

https://ec.europa.eu/info/sites/info/files/srip/2020/rec-19-003_srip_chap-15.pdf

https://www.mdpi.com/2071-1050/12/23/10194/pdf

https://htai.org/wp-content/uploads/2020/03/HTAi_APF-2019-Background-Paper.pdf

Database: CORE

Date searched 26/02/2021

Search strategy: “horizon scanning”

Retrieved results: 5,582

For peer review only

Hand searched the first 5 results pages.

Included for further assessment: 2

Blackburn, Steven, Cudd, Peter and Hawley, Mark (2010) A horizon scanning system for identifying new telehealth innovations. Global Telemedicine and eHealth Updates: Knowledge Resources, 3. pp. 326-330. ISSN 1998-5509 http://eprints.whiterose.ac.uk/11144/

Enhancing Horizon Scanning by utilizing pre-developed scenarios: analysis of current practice and specification of a process improvement to aid the identification of important ‘weak signals’. https://www.sciencedirect.com/science/article/pii/S0040162517300707

Database: International HTA Database

Date searched: 25/01/2022

Search strategy: “horizon scanning” in title

Retrieved results: 310

Sifted on screen, none included

For peer review only

Appendix C

Figure C.1. PRISMA flow chart

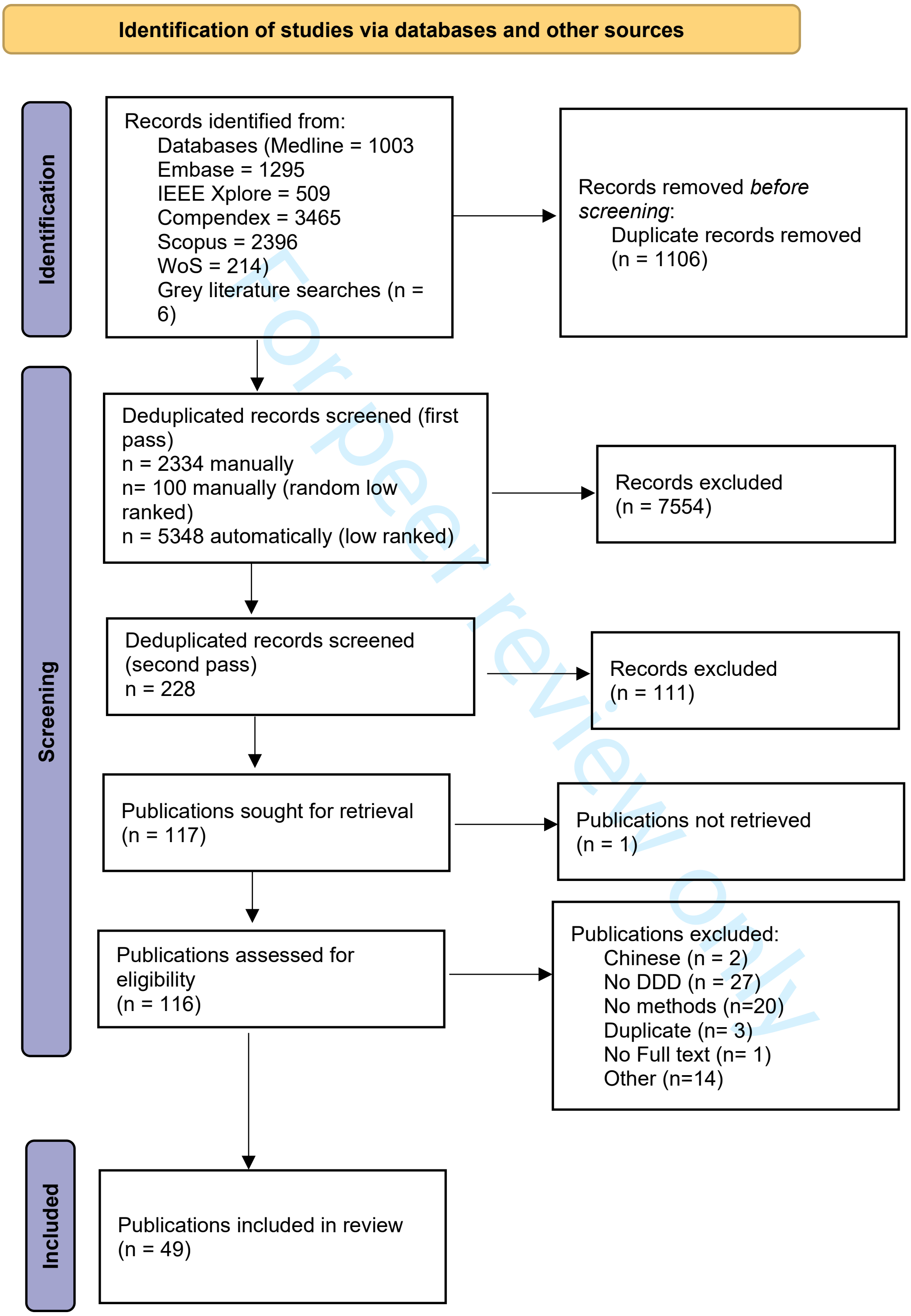

From: Page MJ, McKenzie JE, Bossuyt PM, Boutron I, Hoffmann TC, Mulrow CD, et al. The PRISMA 2020 statement: an updated guideline for reporting systematic reviews. BMJ 2021;372:n71. doi: 10.1136/bmj.n71

For peer review only

BMJ Open

Looking at the fringes of MedTech innovation: a mapping review of horizon scanning and foresight methods

Journal:	*BMJ Open*
Manuscript ID	bmjopen-2023-073730.R1
Article Type:	Original research
Date Submitted by the Author:	06-Jul-2023
Complete List of Authors:	Garcia Gonzalez-Moral, Sonia; Newcastle University, NIHR Innovation Observatory at Population Health Sciences Institute<br>Beyer, Fiona ; Newcastle University, NIHR Innovation Observatory at Population Health Sciences Institute<br>Oyewole, Anne; Newcastle University, Population Health Sciences Institute<br>Richmond, Catherine; Newcastle University, NIHR Innovation Observatory at Population Health Sciences Institute<br>Wainwright, Luke; Newcastle University, Population Health Sciences Institute<br>Craig, Dawn; Newcastle University, NIHR Innovation Observatory at Population Health Sciences Institute
**Primary Subject Heading**:	Health policy
Secondary Subject Heading:	Health services research, Health informatics
Keywords:	Decision Making, Health policy < HEALTH SERVICES ADMINISTRATION & MANAGEMENT, BIOTECHNOLOGY & BIOINFORMATICS, Systematic Review

SCHOLARONE™
Manuscripts

I, the Submitting Author has the right to grant and does grant on behalf of all authors of the Work (as defined in the below author licence), an exclusive licence and/or a non-exclusive licence for contributions from authors who are: i) UK Crown employees; ii) where BMJ has agreed a CC-BY licence shall apply, and/or iii) in accordance with the terms applicable for US Federal Government officers or employees acting as part of their official duties; on a worldwide, perpetual, irrevocable, royalty-free basis to BMJ Publishing Group Ltd ("BMJ") its licensees and where the relevant Journal is co-owned by BMJ to the co-owners of the Journal, to publish the Work in this journal and any other BMJ products and to exploit all rights, as set out in our licence.

The Submitting Author accepts and understands that any supply made under these terms is made by BMJ to the Submitting Author unless you are acting as an employee on behalf of your employer or a postgraduate student of an affiliated institution which is paying any applicable article publishing charge ("APC") for Open Access articles. Where the Submitting Author wishes to make the Work available on an Open Access basis (and intends to pay the relevant APC), the terms of reuse of such Open Access shall be governed by a Creative Commons licence – details of these licences and which Creative Commons licence will apply to this Work are set out in our licence referred to above.

Other than as permitted in any relevant BMJ Author's Self Archiving Policies, I confirm this Work has not been accepted for publication elsewhere, is not being considered for publication elsewhere and does not duplicate material already published. I confirm all authors consent to publication of this Work and authorise the granting of this licence.

For peer review only

Title: Looking at the fringes of MedTech innovation: a mapping review of horizon scanning and foresight methods

Authors:

Lead and Corresponding author: Gonzalez-Moral, Sonia G[1] MSc, Dip(HE), BSc (Hons), ORCID 0000-0003-0431-4771 Sonia.garcia-gonzalez-moral@newcastle.ac.uk

Second Author: Beyer, Fiona R[1] PhD, PGDip, BSc (Hons), ORCID 0000-0002-6396-3467 Fiona.beyer@ncl.ac.uk

Third Author: Oyewole, Anne O[2] PhD, MRes, BSc ORCID 0000-0002-1311-1485 Anne.oyewole@newcastle.ac.uk

Fourth Author: Richmond, Catherine[1] MA, BSc (Hons) wassatwork@gmail.com

Fifth Author: Wainwright, Luke[2] PhD ORCID 0000-0003-3742-6411 luke.wainwright@nhs.net

Last Author: Craig, Dawn[1] MSc, BSc (Hons) Professor of Evidence Synthesis, ORCID 0000-0002-5808-0096 Dawn.craig@ncl.ac.uk

Affiliations

[1] NIHR Innovation Observatory, Population Health Sciences Institute, Newcastle University

[2] Visiting Researcher, Population Health Sciences Institute, Newcastle University

Abstract

Objectives: Horizon-scanning (HS) is a method used to examine signs of change and may be used in foresight practice. HS methods used for the identification of innovative medicinal products cannot be applied in MedTech due to differences in development and regulatory processes. The aim of this study is to identify HS and other methodologies used for MedTech foresight in support to healthcare decision-making.

Method: A mapping review was performed. We searched bibliographic databases including MEDLINE, Embase, Scopus, Web of Science, IEEE Xplore and Compendex Engineering Village and grey literature

For peer review only

sources such as Google, CORE database and the International HTA database. Our searches identified 8,888 records. After deduplication, and manual and automated title, abstracts and full-text screening, forty-nine papers met the inclusion criteria and were data extracted.

Results: Twenty-five single different methods were identified, often used in combination, of these, only three were novel (appearing only once in the literature). Text mining or artificial intelligence solutions appear as early as 2012, often practiced in patent and social media sources. The time horizon used in scanning was not often justified. Some studies regarded experts both as a source and as a method. Literature searching remains one of the most used methods for innovation identification. HS methods were vaguely reported, but often involved consulting with experts and stakeholders.

Conclusion: Heterogeneous methodologies, sources and time horizons are used for HS and foresight of MedTech innovation with little or no justification provided for their use. This review revealed an array of known methods being used in combination to overcome the limitations posed by single methods. The review also revealed inconsistency in methods reporting, with a lack of any consensus regarding best practice. Greater transparency in methods reporting and consistency in methods use would contribute to increased output quality to support informed timely decision-making.

Strengths and limitations of this study

- This review updates the methodological knowledge on horizon scanning practice for health and care decision-making of MedTech innovations.
- This is the first time a systematic literature search is expanded beyond traditional horizon scanning methods to identify other foresight methods applied in MedTech innovation detection and prediction.
- Quality appraisal of the included studies was deemed out of scope and was not performed.

Keywords:

Review; Foresight; Horizon Scanning; Medical Technologies; Technology Assessment, Biomedical; Decision Making

Word count: 3694

For peer review only

Introduction

Horizon-scanning (HS), understood as the a systematic examination of information sources to detect early signs of important developments,[1] is a reliable method to inform legal, regulatory or procurement decisions on new and emerging health technologies.[1,2] HS is a well-established method to support early awareness systems for new and emerging medicines;[3] wider uses of HS include some early work applied to models of care.[4] This paper focuses on the use of HS for emerging innovative medical technologies detection and prioritisation. The strategic importance of HS is recognised and endorsed at the highest level; for example, in the UK, the 2016 independent Accelerated Access Review recommended that the NHS should develop an enhanced HS process to identify products that have the most potential to deliver improved outcomes or efficiencies, acknowledging a more comprehensive and transparent HS system is necessary, especially for non-pharmaceutical products. This task was mandated to the NHS Accelerated Access Collaborative (AAC) by the Secretary of State in 2018. The 2019 'NHS Long Term Plan' sets out to accelerate innovation and patient access to innovative therapies.[5] More recently the 2021 Government's 'Life Sciences Vision' policy statement aims to stimulate industry growth and success for the benefit of patients and the NHS.[6] Both high level national policies advocate for the use of HS to achieve their goals, a sentiment that is echoed in the Government's 'Saving and Improving Lives: The Future of UK Clinical Research Delivery' policy paper (2021) and associated Implementation Plan (2022) which reinforces the AAC's commitment 'to improve identification of the most needed treatments and technologies and rapidly bring these into clinical use' through horizon scanning.

Foresight and HS methods share similar concepts and purpose but differ as follows. The objectives of foresight activities can vary across projects but generally aim to open discussion and debate in anticipation of strategy formulation with the aim of equipping organisations navigating an uncertain future. In HS, however, the aim is the identification of emerging issues or signals and their targets and connections within to elicit a plan of action.[7] The Innovation Observatory (IO) is the horizon scanning and research intelligence organisation funded by the National Institute for Health and Care Research and hosted by Newcastle University in the UK. Based on our experience in the practice of HS in the context of healthcare innovations, we observe that well-conducted HS is a complex and time-consuming process that requires a

For peer review only

range of skills and a depth of knowledge to facilitate the identification, interpretation, and filtration of relevant signals, in addition to highly developed technological tools to support the processing and management of data and knowledge and facilitate its dissemination in a timely manner. Other analytical foresight methods such as patent, bibliometric and trend analyses as well as non-analytical methods such as backcasting, roadmaps or Delphi studies are available that could complement or help solve some of these challenges when applied to the identification and prediction of MedTech innovation, but to these authors' knowledge there is no evidence to support their use in any specific scenario.

Medical technologies (MedTech, including devices, diagnostics and digital health applications and systems) have the potential to transform care pathways, improve health and contribute to cost efficiencies in the healthcare system.[8] HS methods used to signal new medicinal products cannot be directly translated and applied to MedTech due to the challenges posed by the intrinsic characteristics of these technologies' development and regulatory pathways. These challenges are wide ranging and multi-layered, however for the purposes of this paper we will concentrate on those that affect the identification of these technologies ahead of their placement on the market. Firstly, in order to discern where the true value for the healthcare system lies a common definition and interpretation of 'innovation' is needed.[9] Innovation in MedTech may encompass a completely new device class or newer iterations of the same product.[10] Secondly, different regulators of MedTech follow different criteria and requisites for their assessment. These differences have an impact on how and when information is shared with regulators, and whether it can be made available to the wider public. In turn, this has an impact on the ability of HS searches to retrieve and identify signals of commercial readiness. For example, whilst the US Food and Drug Administration (FDA) maintains medical devices databases that can be freely accessed,[11] the EU regulator has not made this information available to the wider public yet.[12] Thirdly, traditional published literature sources might not fully contribute to identify emerging or early innovative MedTech. Whilst this is also the case in HS practice for innovative medicinal products, for MedTech, grey literature, mainly originated by manufacturers, acts as the main source in up to 50% of the time.[13]

Previous reviews into HS methods in healthcare have been undertaken[1,14] however, none of them have looked at how these methods perform when applied to the MedTech innovation pipeline. This review aims

For peer review only

to build from what is already known by broadening the scope to identify foresight methods applied to MedTech innovation identification or prediction, including but not limited to HS; and, to explore the drivers that lead to the application of these methodologies in the context of MedTech to gain an insight into the rationale for choosing a particular method over others. This work is the first step for devising a methodological framework to guide foresight practice in MedTech innovation decision-making.

Methods

In line with the core principles for conducting mapping reviews outlined by the Campbell Collaboration[15], this review aimed to identify evidence of use of HS and foresight methods for MedTech innovation detection. A protocol was agreed amongst the review team which guided the inclusion and exclusion of studies as well as the systematic literature searching in databases and grey literature sources. This review did not undertake quality appraisal of the included studies as this was considered out of scope. PRISMA-S extension for reporting systematic literature searches was followed and reported in Appendix A, Table A.1.[16]

Patient and public involvement

Members of the public were not involved in this review.

Inclusion criteria

Studies were eligible for inclusion if: i) any of the following methods were mentioned in the text: environmental scanning, HS, foresight, forecasting, roadmapping, patent, bibliometric or trend analyses, backcasting or Delphi studies; ii) a medical health technology such as a medical device, diagnostic, digital intervention, or all was included; iii) published in English.

Search strategy

The search strategy consisted of a combination of subject headings and text words in title, abstracts and key word fields. The strategy was peer-reviewed by an independent information specialist using the PRESS review statement.[17] The final MEDLINE strategy was translated and run separately in: Embase, Scopus, Web of Science, IEEE Xplore and Compendex Engineering Village. Grey literature sources (Google and

130 CORE[18]) and International Horizon Scanning organizations (via International HTA Database) were searched. No time or language limits were used. Detailed search strategies are included in Appendix B. Reference lists of included papers were browsed to identify potential papers missed. Search results were downloaded to Endnote20 (Clarivate Analytics) and deduplicated.

Data collection

Results were de-duplicated and screened in two stages using Rayyan, a semi-automated screening tool for systematic reviews.[19] At stage one the main reviewer screened the title and abstracts, with each vote for inclusion or exclusion, Rayyan produced a 5-star ranking for all the references uploaded. A random sample of 100 low-ranked (three stars or less) references were screened to assess the consensus of the semi-automated ranking with the reviewer's manual screening decisions.

At second stage, two reviewers manually double screened the full texts and selected those that met the eligibility criteria. Disagreements were reconciled by discussion between the two reviewers.

Data extraction

A custom data extraction form was designed in Excel to record the relevant characteristics of the included papers. The extracted data captured: 1) author name and publication year, 2) title, 3) objective of the article, 4) study type, 5) methods used, 6) sources used, 7) technology type, 8) time horizon, 9) type of output produced and 10) automated methods used.

Data synthesis

Extracted data was further categorised by objectives described and information on methods used to allow for analysis as some discrepancies in the reporting were present. Data was synthesised visually, tabulated and in narrative formats.

Results

Database and grey literature searches identified 8888 records. After de-duplication 7782 remained. The first screening pass included a total of 228 references (more details on this stage on Appendix C, fig.C.1). Two reviewers manually screened the title and abstract of these and excluded a further 111 references. At

For peer review only

second stage, 117 full-text papers were manually double screened, concluding with 49 papers published between 1999 and 2020 that met the inclusion criteria. PRISMA flow-chart included in Appendix C, fig. C.1.

Analysis of objectives

Ten different categories of aims and objectives were identified (fig. 1).

Figure 1. Aims and objectives for using HS/foresight methods in MedTech innovation identification

Methods and sources

The included studies reported 25 single different methods. Twenty-nine studies used a single method[20-45] and 20 used multiple methods in combination.[46-65] The methods that overlap between the group of papers that reported using one method and the group the used more than one in combination were: expert panels, patent searches/analyses, literature searches, stakeholders, HS, roadmapping, Delphi studies, trend analyses, patient engagement and text mining. A small number of uniquely used methods in the combination group encompassed clinical trial analysis, searching approved medical devices databases, hospital data analysis, environmental scan and projective scenarios; while other were uniquely employed by the single-method group: content analysis, forecasting, internet searching, forecast report analysis, panoramic view analytics, scenario-driven road mapping, scientometric mapping approach to overlay mapping, strategic foresight or surveying other horizon scanning organisations.

Figure 2. Methods and sources overlap

Figure 2 represents a Venn diagram for the overlap of methods as reported, the black writing denotes sources and red and purple denotes methods. Methods and sources were often not differentiated. Across both groups, the most frequent and clearly reported method was HS (n=15) followed by surveying or consulting with experts (n=14), literature searching (n=13), patent searching/analysis (n=10), stakeholder engagement (n=4), text/data mining (n=4), trend analysis (n=3), and roadmaps (n=3). Cited less often were report content analyses (n=2),[25,42] Delphi (n=2),[33,57] clinical trial searches (n=2),[47,63] web scraping or mining (n=2),[38,47] and social media searching (n=2).[24,53]

For peer review only

Methods mentioned only once included: environmental scanning,[56] overlay mapping,[34] panoramic view analytics,[66] strategic foresight,[27] surveying companies[23] and surveying other HS organisations.[22]

Forecasting was mentioned by four different reports.[25,32,40,57] One used a combination of expert group opinion forecasting approach, i.e. the Delphi-technique with a market oriented capacity analysis by using projective scenarios.[57] The three remaining cite forecasting as a single method using multiple sources including literature and patent searches.[25,32,40]

Less clearly reported methods, often embedded in the reporting of the sources used, were engagement with stakeholders (7)[23,26,46,49,56,58,67] and surveying or consulting with patients (3).[52,62,68]

Eleven reports published between 2012 and 2020 reported the use of automated methods.[20,24,27,38,40,43,45,47,61,64,66] The most frequently cited was text mining with seven reports describing its use; detailed descriptions of the software packages used was not provided in all instances. Web scraping or URL crawling was described by two reports separately, machine learning (including unsupervised mode) was cited by two reports, data mining was mentioned by two reports and natural language processing (NLP) by one.

The great majority of the included reports (88%) described the sources of data/intelligence used (fig 3).

Figure 3. Type of sources used as described in the included reports

Thirteen reports described the use of published literature sources.[28,34,40,47,49-51,54,60,61,63-65] Of these, 3 did not specify whether they also searched unpublished literature [28,54,60] and a further 12 used published and unpublished sources of literature.[29,31,36,37,39,41,44,46,48,52,53,56]

Literature searching was used in different ways to support the identification of signals. Whilst some reports used it as the basis of the signal identification,[39,41,47,49,52,56,60,65] others used it as a complement to enhance the strength of signals already identified by other means,[36,46,48,50,63] some reports used it in combination with other sources to study relationships[34,40,51,64,69] or expand the signal identification process.[28,29,31,37,44,53,54]

For peer review only

All studies that reported literature searching as a method also used it as a source, however some studies cited literature searching as a source but did not describe it as a method.[28,29,31,34,36,37,39,40,44,56,64]

The analysis of methods reported in the literature in conjunction with the sources used, identified seven reports[28,29,31,36,37,39,44] that used HS as a single method whilst searching multiple sources. Three of those reports[29,31,37] additionally practised some level of expert, stakeholder or patient engagement.

From the group of nine papers that reported the use of HS in combination with other methods, seven included consultation with experts, stakeholders or patients.[41,48,55,56,58,62,68] Since half of those studies that reported using HS as a method also reported the use of experts or stakeholders, it cannot be extrapolated that HS has always involved consulting with experts unless explicitly specified.

Types of technologies included

The most frequently searched for technology were medical devices (n=28), followed by 16 diagnostics, nine digital health interventions and three procedures. Four reports included medical devices, diagnostics and digital health interventions.[44,52,59]

Fifteen reports recorded the inclusion of all technologies but did not provide details and have been analysed as “all inclusive”.[21,22,25,31,33-35,38,45,47,48,53,57,62,68]

Two reports did not specify any technology within the MedTech scope.[27,29]. Table 1 (Appendix D) presents an overview of the included reports in relation to the technologies in scope and the main purpose of the study.

This review did not identify a relationship between the technologies in scope and the method used for their identification.

Time horizons

Time horizons were not always described accurately nor was a rationale for the use of a given time limit provided. Some reports mentioned the time horizon as described in the reporting of the searches and often provided both an upper and a lower time limit for technology identification. The timeframes were

For peer review only

variable and depended on the type of methods used and aim of the study. The longest timeframe analysed was 29 years and the shortest 18 months. Longer timeframes have been associated with patent mining or searching. Time horizons were used retrospectively or prospectively depending on the time endpoint and aim of the study. For example, retrospective searching was used to identify technologies in scope for established early warning systems[52] or to identify opportunities for future technology development by retrospective searching of patents and analysing results[51]. Prospective methods mostly involved consultation with experts such as clinicians, industry representatives and decision-makers for the prioritisation, prediction or validation of signals.[28,35,53]

Figure 4. Development of methods over a publication timeline

As shown in figure 4 ten studies published between 1999 and 2009 used HS, surveys, a Delphi study and internet searches or a combination of methods mainly consisting of literature searches, expert engagement via interviews and stakeholder surveys. A decade later the range of methods had exponentially increased, more authors use a combination of methods, and these are applied to different decision-making scenarios. Automated and machine learning approaches appear as early as 2012 and underpin almost a third of the methodologies reported.

Discussion

This review set out to study how foresight methodologies are used to identify innovative MedTech to support healthcare decision-making. To our knowledge, this review is the first to study the range of foresight methods alongside HS in the context of healthcare decision-making. This review also presents up-to-date information on the sources used for MedTech identification and intelligence analysis in this field. Despite the common understanding that MedTech reports are not usually disseminated by published literature channels, published literature remains as one of the main sources used for MedTech identification which undermines the ability to discover innovation in a timely fashion.[13]

We discovered some interesting methods that laid outside the limits of the established HS techniques more traditionally defined and used by organisations operating in the health technology space. Methods such as a scientometric mapping approach using overlay mapping of three geographical, social and cognitive

For peer review only

spaces[34] provide a visual approach to synthesize complex information and help strategic decision making. Panoramic view analytics[66] also exploit the benefits of visualisation to explore multi-dimensional technological information. Scenario-driven road-mapping[67], a hybrid method developed from traditional road-mapping and scenarios for technology foresight, overcomes some of the limitations of using these methods independently and provides a viable novel method particularly useful in forecasting technology adoption.

The timeframes used by the included studies revealed great variability and lacked justification. We recommend the justification of the timeframe used in consideration with the life cycles of the technology subject of study (e.g., MedTech products 18 to 24 months). Automation techniques generally used for the identification of signals had been reported within the last decade. There is evidence of their use in patents often in combination with other sources such as clinical trial data and social media platforms such as Twitter or the Internet in general. There is potential for application of these techniques to other bodies of data such as news, conference proceedings or multimedia social platforms such as YouTube but they remain still to be explored for the identification of MedTech signals.

This review has unveiled the broad and vaguely reported use of the term 'horizon scanning' often used as an umbrella to describe the range of methodologies used for the identification of signals, which frequently involve searching in multiple sources and potentially consulting with experts, stakeholders or patients and the public. This inconsistency has been reported previously by Hines et al.[1] and most recently by Charlton and colleagues.[70] Given the important role that HS plays in health and care policy and decision-making as outlined in the introduction to this study, there is an urgent need for consistency and robust methods standardisation and reporting. We think that HS methods terminology and robust reporting standards will contribute to increase the precision in the identification of published HS literature. An internationally agreed methods guide could provide the groundwork for better standardisation and retrieval of HS reports. The International Horizon Scanning Initiative[71] and the International HealthTechScan[72] are two networks operating in this space that have the potential to contribute towards greater methods standardisation and the IO has a seat in those two networks to influence and share in enhanced methods initiatives.

For peer review only

Finally, experts, broadly encompassing clinicians, patients and the public, were often used as a method to identify, prioritize or predict technology innovation, as well as a source to compare signals identified by other means. Expert engagement methods were not always specified nor were reported the type of experts (patients, public, and professionals) or their professional backgrounds (clinicians, entrepreneurs, regulators, consultancy companies, industry). There is evidence that suggests expert engagement alone can bias predictions[46] therefore it would be advisable to practice transparency in the reporting of expert selection and engagement methods.

The use of a systematic literature search and reporting methods to identify published and unpublished literature allowed for a robust and reproducible search in a broad range of databases beyond the traditional medical databases. Further, we believe that this is the first mapping review that aims to discover foresight and HS methods for MedTech innovations to support decision-making. The analysis of objectives, methods and sources together may contribute to answer some of the epistemological questions around which foresight method to use to detect future MedTech development relevant to healthcare decision-makers.[9,73] This review has some limitations. Firstly, the search was geared towards identifying several 'known' methods and that may have limited the possibility of identifying new or unknown methods. However, as already demonstrated, new methods were identified possibly due to the broad inclusion criteria. Secondly, screening was undertaken mostly by one author with the support of an automated screening software and a second checker. Although some random sampling for screening was undertaken, some potentially valid records could have been missed due to poor indexing or lack of abstract. Concept visualisation of those records could have been used to identify further potentially relevant records; however, due to time and resource constrains this option was not explored. Lastly, the last search was undertaken in 2021 and no further searches have been run since then. Although it is possible that new methods have been published since, they are unlikely to change the conclusions of this review.

Conclusion and recommendations

In the healthcare field there is global interest in understanding the MedTech innovation pipeline, with a view on system preparedness and acceleration of technology in areas of unmet need. Exploiting HS and foresight methods will support advanced signalling and system preparedness to harness the opportunities

For peer review only

posed by innovative MedTech, but also allow us time to address some of the future challenges ahead of those technologies being placed on the market. This review has revealed the use of a range of different methods and tools, used singly or in combination; manually applied or supported with AI (text mining and machine learning). These methods have not been applied in a transparent or consistent manner making an assessment of their validity and value challenging. However, they may offer potential solutions to the current methods limitations and further research in this space should be encouraged. There is also a need to understand the value of alternative sources of unpublished literature such as pre-print repositories, conference proceedings or specialist technology-focussed sources to mitigate the publication bias already acknowledged. The IO has successfully used pre-print repositories for the identification of novel treatments during the recent COVID-19 pandemic[74] and will continue to build on this work. In many areas of research expert engagement is commonly used. Following an equitable and transparent approach to recruitment and involvement, similar to schemes for Patient and Public Involvement and Engagement (PPI&E) such as the UK Standards for Public Involvement in Research,[75] should be standard in the practice of foresight. Most importantly, this review clearly highlighted a scientific hindrance due to the lack of methods standardisation and reporting. Based on the findings from this study some recommendations to improve methods reporting have been suggested in Box 1.

Studying impact of HS intelligence in the decision-making process was out of scope of this review, nevertheless, is an important future research need. This review has provided the foundation work for the next steps in the programme of the National Institute for Health and Care Research Innovation Observatory[76] work which will focus on the development of agreed standards for reporting methods and the development of HS methods guides.

Box 1. Recommendations for future methods standardisation

1. Uniformly use of terminology for the description of methods
2. Robust reporting of sources searched, and strategies used
3. Rationale for the use of time limits
4. Practice transparency when reporting the background of experts involved

For peer review only

5. Report functionality of automated methods when used

Author Contributions:

Sonia G Gonzalez-Moral was responsible for the conceptualization of this project, methodology, writing of the original draft, visualization of results and the overall project administration. Fiona R Beyer, Anne O Oyewole and Luke Wainwright were involved in the revising and editing of this review and the supervision of this work. Catherine Richmond was involved in the validation of the search strategy, record sifting and writing of original draft. Dawn Craig was responsible for writing and editing of this review, supervision of this work and funding acquisition.

Competing Interests:

None declared

Funding:

This study/project is funded by the National Institute for Health & Care Research (NIHR) [NIHRIO/project reference HSRIC-2016-10009]. The views expressed are those of the author(s) and not necessarily those of the NIHR or the Department of Health and Social Care.

Data sharing statement:

All data relevant to the study are included in the article or uploaded as online supplemental information.

Ethics statements:

Patient consent for publication was not applicable. This research did not require institutional review board approval as the data were publicly available and collected from existing online databases. This research did not involve any human subjects.

References

1. Hines P, Hiu Yu L, Guy RH, et al. Scanning the horizon: a systematic literature review of methodologies. *BMJ Open* 2019;9(5):e026764-e64. doi: 10.1136/bmjopen-2018-026764
2. Amanatidou E, Butter M, Carabias V, et al. On concepts and methods in horizon scanning: Lessons from initiating policy dialogues on emerging issues. *Science and Public Policy* 2012;39(2):208-21. doi: doi.org/10.1093/scipol/scs017
3. Stevens A, Packer C, Robert G. Early Warning of New Health Care Technologies in the United Kingdom. *Int J Technol Assess Health Care* 1998;14(4):680-86. doi: 10.1017/S0266462300011995. [published Online First: 2009/03/10]
4. Kjelsnes AA, Feiring E. Models of integrated care for older people with frailty: a horizon scanning review. *BMJ Open* 2022;12(4):e060142. doi: 10.1136/bmjopen-2021-060142
5. National Health Service (NHS). The NHS Long Term Plan: NHS 2019.
6. HM Government. Life Sciences Vision, 2021.

For peer review only

7. Cuhls KE. Horizon Scanning in Foresight – Why Horizon Scanning is only a part of the game. *Futures & Foresight Science* 2020;2(1):e23. doi: 10.1002/ffo2.23
8. MedTech Europe. MedTech Europe: About the Industry 2020 [Available from: https://www.medtecheurope.org/about-the-industry/].
9. Bauer W, Schimpf S. Back to the Future: Scoping Dynamics in Industrial Innovation. *IEEE Engineering Management Review* 2020;48(2):72-82.
10. Fuchs S, Olberg B, Panteli D, et al. HTA of medical devices: Challenges and ideas for the future from a European perspective. *Health Policy* 2017;121(3):215-29. doi: 10.1016/j.healthpol.2016.08.010
11. U.S. Food and Drugs Administration. FDA Medical Device Databases 2021 [Available from: https://www.fda.gov/medical-devices/device-advice-comprehensive-regulatory-assistance/medical-device-databases].
12. European Commission. EUDAMED - European Database on Medical Devices [Available from: https://ec.europa.eu/tools/eudamed/#/screen/home; accessed 22 January 2023].
13. Farrah K, Mierzwinski-Urban M. Almost half of references in reports on new and emerging nondrug health technologies are grey literature. *J Med Libr Assoc* 2019;107(1):43-48. doi: doi.org/10.5195/jmla.2019.539
14. Doos L, Packer C, Ward D, et al. Past speculations of the future: a review of the methods used for forecasting emerging health technologies. *BMJ Open* 2016;6(3):e010479. doi: 10.1136/bmjopen-2015-010479
15. White H, Albers B, Gaarder M, et al. Guidance for producing a Campbell evidence and gap map. *Campbell Systematic Reviews* 2020;16(4):e1125. doi: 10.1002/cl2.1125
16. Rethlefsen ML, Kirtley S, Waffenschmidt S, et al. PRISMA-S: an extension to the PRISMA Statement for Reporting Literature Searches in Systematic Reviews. *Syst Rev* 2021;10(1):39. doi: 10.1186/s13643-020-01542-z PMCID: PMC7839230. [published Online First: 2021/01/28]
17. McGowan J, Sampson M, Salzwedel DM, et al. PRESS Peer Review of Electronic Search Strategies: 2015 Guideline Statement. *Journal of Clinical Epidemiology* 2016;75:40-46. doi: 10.1016/j.jclinepi.2016.01.021
18. The Open University. CORE [updated 27/02/2023. Available from: https://core.ac.uk/].
19. Ouzzani M, Hammady H, Fedorowicz Z, et al. Rayyan-a web and mobile app for systematic reviews. *Syst Rev* 2016;5(1):210. doi: 10.1186/s13643-016-0384-4 PMCID: PMC5139140. [published Online First: 2016/12/07]
20. de Souza Antunes AM, de Menezes Alencar MS, da Silva CH, et al. Trends in nanotechnology patents applied to the health sector. *Recent Patents on Nanotechnology* 2012;6(1):29-43. doi: 10.2174/187221012798109309
21. Douw K, Vondeling H, Eskildsen D, et al. Use of the internet in scanning the horizon for new and emerging health technologies: A survey of agencies involved in horizon scanning. *Journal of Medical Internet Research* 2003;5(1):33-45. doi: 10.2196/jmir.5.1.e6
22. Ibargoyen-Roteta N, Gutierrez-Ibarluzea I, Asua J, et al. Scanning the horizon of obsolete technologies: Possible sources for their identification. *International Journal of Technology Assessment in Health Care* 2009;25(3):249-54. doi: 10.1017/S0266462309990249 PMCID: 19619342
23. Itoh S, Kano S. Technology Forecasting for Medical Devices Guidance Formulation: A Case Study in Japan. *Therapeutic Innovation and Regulatory Science* 2019;53(4):481-89. doi: 10.1177/2168479018793370 PMCID: 30175593
24. Lee J, Kim J, Hong YJ, et al. Health information technology trends in social media: Using twitter data. *Healthcare Informatics Research* 2019;25(2):99-105. doi: 10.4258/hir.2019.25.2.99
25. Lerner JC, Robertson DC, Goldstein SM. Case studies on forecasting for innovative technologies: Frequent revisions improve accuracy. *Health Affairs* 2015;34(2):311-18. doi: 10.1377/hlthaff.2014.1066 PMCID: 25646112
26. Migliore A, Perrini MR, Jefferson T, et al. Implementing a national early awareness and alert system for new and emerging health technologies in Italy: The COTE project. *International Journal of Technology Assessment in Health Care* 2012;28(3):321-26. doi: 10.1017/S0266462312000384
27. Mühlroth C, Grottke M. Artificial Intelligence in Innovation: How to Spot Emerging Trends and Technologies. *IEEE Transactions on Engineering Management* 2020;PP(99):Jan-18. doi: 10.1109/TEM.2020.2989214
28. Mundy L, Merlin TL, Parrella A, et al. The Australia and New Zealand horizon scanning network. *Australian health review : a publication of the Australian Hospital Association* 2005;29(4):395-97. doi: 10.1071/AH050395

29. O'Malley SP, Jordan E. Horizon scanning of new and emerging medical technology in Australia: Its relevance to medical services advisory committee health technology assessments and public funding. *International Journal of Technology Assessment in Health Care* 2009;25(3):374-82. doi: 10.1017/S0266462309990031
30. Ouyang Z-L, Fan Y-B, Zhou P, et al. Identification of hotspot technologies in the field of radiation diagnosis based on bibliometric analysis of global patents. 2014 International Conference on Information Science, Electronics and Electrical Engineering, ISEEE 2014, April 26, 2014 - April 28, 2014. Sapporo City, Hokkaido, Japan: Institute of Electrical and Electronics Engineers Inc., 2014:84-88.
31. Packer C. The National Horizon Scanning Centre (NHSC): Early warning for new and emerging health technologies in England. *Evidence-Based Healthcare and Public Health* 2005;9(6):410-13. doi: 10.1016/j.ehbc.2005.09.005
32. Pretorius L, Winzker DH. Biomedical technology: A case study of forecasting in pulsed electro magnetic field therapy. PICMET '10 - Portland International Center for Management of Engineering and Technology, Proceedings - Technology Management for Global Economic Growth: IEEE Computer Society, 2010:248-53.
33. Robert G, Stevens A, Gabbay J. 'Early warning systems' for identifying new healthcare technologies. *Health Technology Assessment* 1999;3(13):iii-99. doi: 10.3310/hta3130 PMCID: 10632623
34. Rotolo D, Rafols I, Hopkins MM, et al. Strategic intelligence on emerging technologies: Scientometric overlay mapping. *Journal of the Association for Information Science and Technology* 2017;68(1):214-33. doi: 10.1002/asi.23631
35. Stafinski T, Topfer LA, Zakariasen K, et al. The role of surgeons in identifying emerging technologies for health technology assessment. *Canadian Journal of Surgery* 2010;53(2):86-92. PMCID: PMC2845954
36. Storz P, Kolpatzik K, Perleth M, et al. Future relevance of genetic testing: A systematic horizon scanning analysis. *International Journal of Technology Assessment in Health Care* 2007;23(4):495-504. doi: 10.1017/S0266462307070596
37. Tal O, Hakak N. Early awareness and alert systems for medical technologies in Israel. *International Journal of Technology Assessment in Health Care* 2012;28(3):333-38. doi: 10.1017/S0266462312000396
38. Thorleuchter D, Van Den Poel D. Weak signal identification with semantic web mining. *Expert Systems with Applications* 2013;40(12):4978-85. doi: 10.1016/j.eswa.2013.03.002
39. Trevitt S, Simpson S, Wood A. Artificial Pancreas Device Systems for the Closed-Loop Control of Type 1 Diabetes: What Systems Are in Development? *Journal of Diabetes Science and Technology* 2016;10(3):714-23. doi: 10.1177/1932296815617968
40. Vaganova EV, Syryamkin MV. Forecasting of the development of professional medical equipment engineering based on neuro-fuzzy algorithms. 5th International Scientific Conference on New Operational Technologies, NEWOT 2015, 2015.
41. Varela-Lema L, Punal-Rioboo J, Accion BC, et al. Making processes reliable: A validated pubmed search strategy for identifying new or emerging technologies. *International Journal of Technology Assessment in Health Care* 2012;28(4):452-59. doi: 10.1017/S0266462312000578
42. Verbakel JY, Turner PJ, Thompson MJ, et al. Common evidence gaps in point-of-care diagnostic test evaluation: a review of horizon scan reports. *BMJ Open* 2017;7(9):e015760. doi: https://dx.doi.org/10.1136/bmjopen-2016-015760
43. Wang J, Chen Y-J. A novelty detection patent mining approach for analyzing technological opportunities. *Advanced Engineering Informatics* 2019;42 doi: 10.1016/j.aei.2019.100941
44. Wong WQ, Lin L, Ju H, et al. Towards greater impact in health technology assessment: Horizon scanning for new and emerging technologies in Singapore. *International Journal of Technology Assessment in Health Care* 2020;36(4):304-10. doi: 10.1017/S0266462320000343
45. Ye C, Feng L. Future-oriented technology analysis of technology roadmap based on text mining. 2013 10th International Conference on Fuzzy Systems and Knowledge Discovery (FSKD), 2013:1126-30.
46. Apreda R, Bonaccorsi A, dell'Orletta F, et al. Expert forecast and realized outcomes in technology foresight. *Technological Forecasting and Social Change* 2019;141:277-88. doi: 10.1016/j.techfore.2018.12.006
47. Baskici C, Atan S, Ercil Y. Forecasting of innovation in the light of semantic networks. 3rd World Conference on Technology, Innovation and Entrepreneurship, WOCTINE 2019, June 21, 2019 - June 23, 2019. Istanbul, Turkey: Elsevier B.V., 2019:443-49.
48. Bhavnani SP, Parakh K, Atreja A, et al. 2017 Roadmap for Innovation-ACC Health Policy Statement on Healthcare Transformation in the Era of Digital Health, Big Data, and Precision Health: A Report of the American College of Cardiology Task Force on Health Policy Statements and Systems of Care. *Journal of the American College of Cardiology* 2017;70(21):2696-718. doi: 10.1016/j.jacc.2017.10.018

For peer review only

49. Brown IT, Smale A, Verma A, et al. Medical technology horizon scanning. *Australasian Physical and Engineering Sciences in Medicine* 2005;28(3):200-03. doi: 10.1007/BF03178717 PMCID: 16250477
50. Chalkidou A, Erskine J, Macmillan T, et al. Factors affecting horizon scanning for hospital-based health technology assessment. 16th HTAi's Annual Meeting. Germany: International Journal of Technology Assessment in Health Care, 2019:25-26.
51. Chang SWC, Trappey CV, Trappey AJC, et al. Forecasting dental implant technologies using patent analysis. 2014 Portland International Center for Management of Engineering and Technology, PICMET 2014, July 27, 2014 - July 31, 2014. Kanazawa, Japan: Institute of Electrical and Electronics Engineers Inc., 2014:1483-91.
52. Dixon LC, Ward DJ, Smith J, et al. New and emerging technologies for the diagnosis and monitoring of chronic obstructive pulmonary disease. *Chronic Respiratory Disease* 2016;13(4):321-36. doi: 10.1177/1479972316636994
53. Flick C, Zamani ED, Stahl BC, et al. The future of ICT for health and ageing: Unveiling ethical and social issues through horizon scanning foresight. *Technological Forecasting and Social Change* 2020;155 doi: 10.1016/j.techfore.2020.119995
54. Gelderblom GJ, De Wilt M, Cremers G, et al. Rehabilitation robotics in robotics for healthcare; a roadmap study for the European Commission. 2009 IEEE International Conference on Rehabilitation Robotics, ICORR 2009, June 23, 2009 - June 26, 2009. Kyoto, Japan: IEEE Computer Society, 2009:834-38.
55. Handa N, Mochizuki S, Fujiwara Y, et al. Future development of artificial organs related with cutting edge emerging technology and their regulatory assessment: PMDA's perspective. *Journal of Artificial Organs* 2020;23(3):203-06. doi: 10.1007/s10047-020-01161-4 PMCID: 32112156
56. Morrison A. Scanning the horizon in a decentralized healthcare system: The Canadian experience. *International Journal of Technology Assessment in Health Care* 2012;28(3):327-32. doi: 10.1017/S0266462312000323
57. Postma TJBM, Alers JC, Terpstra S, et al. Medical technology decisions in The Netherlands: How to solve the dilemma of technology foresight versus market research? *Technological Forecasting and Social Change* 2007;74(9):1823-33. doi: 10.1016/j.techfore.2007.05.011
58. Ruggeri M, Cadeddu C, Roazzi P, et al. Multi–criteria–decision–analysis (MCDA) for the horizon scanning of health innovations an application to COVID 19 emergency. *International Journal of Environmental Research and Public Health* 2020;17(21):1-9. doi: 10.3390/ijerph17217823 PMCID: 33114587
59. Sheikh NJ, Sheikh O. Forecasting of biosensor technologies for emerging point of care and medical IoT applications using bibliometrics and patent analysis. 2016 Portland International Conference on Management of Engineering and Technology, PICMET 2016, September 4, 2016 - September 8, 2016. Honolulu, HI, United states: Institute of Electrical and Electronics Engineers Inc., 2016:3082-93.
60. Shekelle PG, Goldman DP. Development of a Group Judgment Process for Forecasts of Health Care Innovations. *JAMA network open* 2018;1(7):e185108. doi: 10.1001/jamanetworkopen.2018.5108 PMCID: 30646380
61. Shen Y-C, Wang M-Y, Yang Y-C. Discovering the potential opportunities of scientific advancement and technological innovation: A case study of smart health monitoring technology. *Technological Forecasting and Social Change* 2020;160 doi: 10.1016/j.techfore.2020.120225
62. Smith J, Ward D, Michaelides M, et al. New and emerging technologies for the treatment of inherited retinal diseases: A horizon scanning review. *Eye (Basingstoke)* 2015;29(9):1131-40. doi: 10.1038/eye.2015.115 PMCID: 26113499
63. Tark JY, Jeong J-Y, Lee M, et al. Early assessment and prediction of potential impact of the implantation of polyurethane scaffolf in partial meniscal lesions: a pilot horizon scanning activity in South Korea. *International Journal of Technology Assessment in Health Care* 2015;31(6):380-89. doi: 10.1017/S0266462315000689
64. Trappey CV, Trappey AJC, Peng H-Y, et al. A knowledge centric methodology for dental implant technology assessment using ontology based patent analysis and clinical meta-analysis. *Advanced Engineering Informatics* 2014;28(2):153-65. doi: 10.1016/j.aei.2014.03.001
65. Varela-Lema L, De La Fuente-Cid R, Lopez-Garcia M. Developing a prioritized list of innovative technologies: the Spanish experience. *International Journal of Technology Assessment in Health Care* 2014;30(6):626-33. doi: 10.1017/S0266462314000774
66. Hayashi T. R&D trend analysis of wellbeing AI using panoramic view analytics. 2017 AAAI Spring Symposium, March 27, 2017 - March 29, 2017. Stanford, CA, United states: AI Access Foundation, 2017:673-74.

67. Hussain M, Tapinos E, Knight L. Scenario-driven roadmapping for technology foresight. *Technological Forecasting and Social Change* 2017;124:160-77. doi: 10.1016/j.techfore.2017.05.005

68. Simpson S, Cook A, Miles K. Patient and public involvement in early awareness and alert activities: An example from the United Kingdom. *International Journal of Technology Assessment in Health Care* 2018;34(1):10-17. doi: 10.1017/S0266462317004421

69. Hospodkova P, Bartak M, Vacíková K, et al. Global centers of medical device technology: United States, Europe and China. *Lekar a Technika* 2018;48(4):136-44.

70. Charlton P, Kean T, Liu RH, et al. Use of environmental scans in health services delivery research: a scoping review. *BMJ Open* 2021;11(11):e050284. doi: 10.1136/bmjopen-2021-050284

71. International Horizon Scanning Initiative. Horizon Scanning System 2023 [Available from: https://ihsi-health.org/horizon-scanning-system/; accessed 20/02/2023].

72. EuroScan International Network. International HealthTechScan (i-HTS) 2022 [Available from: https://www.i-hts.org/network; accessed 20/02/2023].

73. Lauster M, Hansen-Casteel S. On some fundamental methodological aspects in foresight processes. *European Journal of Futures Research* 2018;6(1):11. doi: 10.1186/s40309-018-0140-1

74. Gonzalez-Moral SG, Al-Assaf A, Pandey S, et al. OP218 Searching Preprint Repositories For COVID-19 Therapeutics Using A Semi-Automated Text-Mining Tool. *Int J Technol Assess Health Care* 2021;37(S1):6-6. doi: 10.1017/S0266462321000799. [published Online First: 2021/12/03]

75. UK Standards for Public Involvement. 2020 [Available from: https://sites.google.com/nihr.ac.uk/pi-standards/home; 29/06/2023].

76. National Institute for Health and Care Research Innovation Observatory (NIHRIO). 2022 [Available from: https://www.io.nihr.ac.uk/; accessed 12/12/2022].

For peer review only

Figure 1. Aims and objectives for using HS/foresight methods in MedTech innovation identification

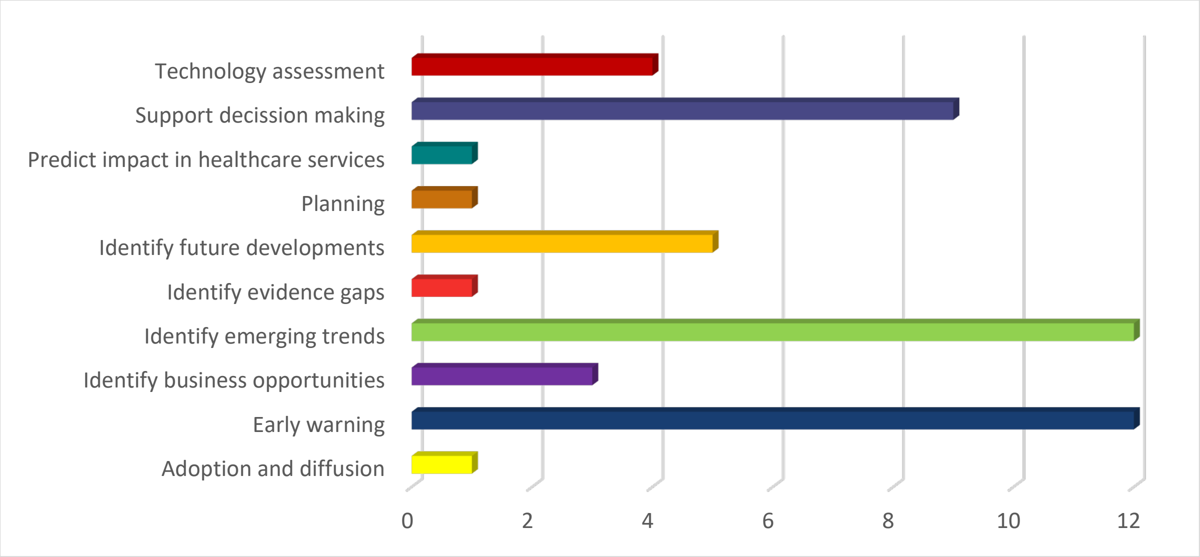

Early warning (refs 18- 29); Identification of emerging trends (refs 30-41); Supporting decision-making (refs 42-50); Identification of future developments (refs 51-55); Supporting technology assessment processes (refs 56-59); Identification of business opportunities (refs 60-62); Predict impact on healthcare services (ref 63); Planning and evaluation (ref 64); Prediction of adoption and diffusion (ref 65); and Identification of evidence gaps (ref 66).

For peer review only - http://bmjopen.bmj.com/site/about/guidelines.xhtml

Figure 2. Methods and sources overlap

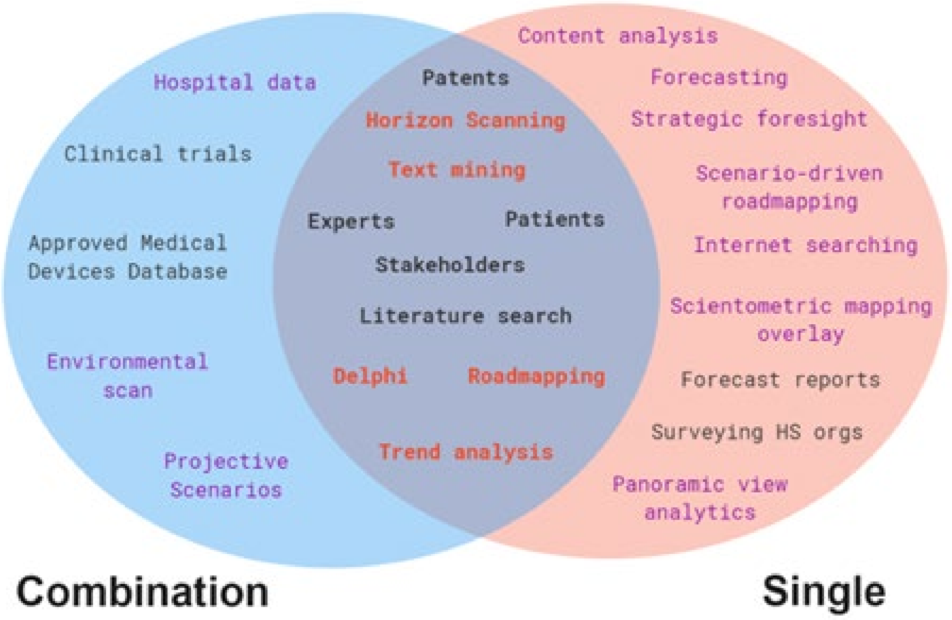

Black denotes sources; Red denotes methods used by single or combination studies; Purple denotes methods only appearing once in any study (combination or single)

For peer review only

Figure 3. Type of sources used as described in the included reports

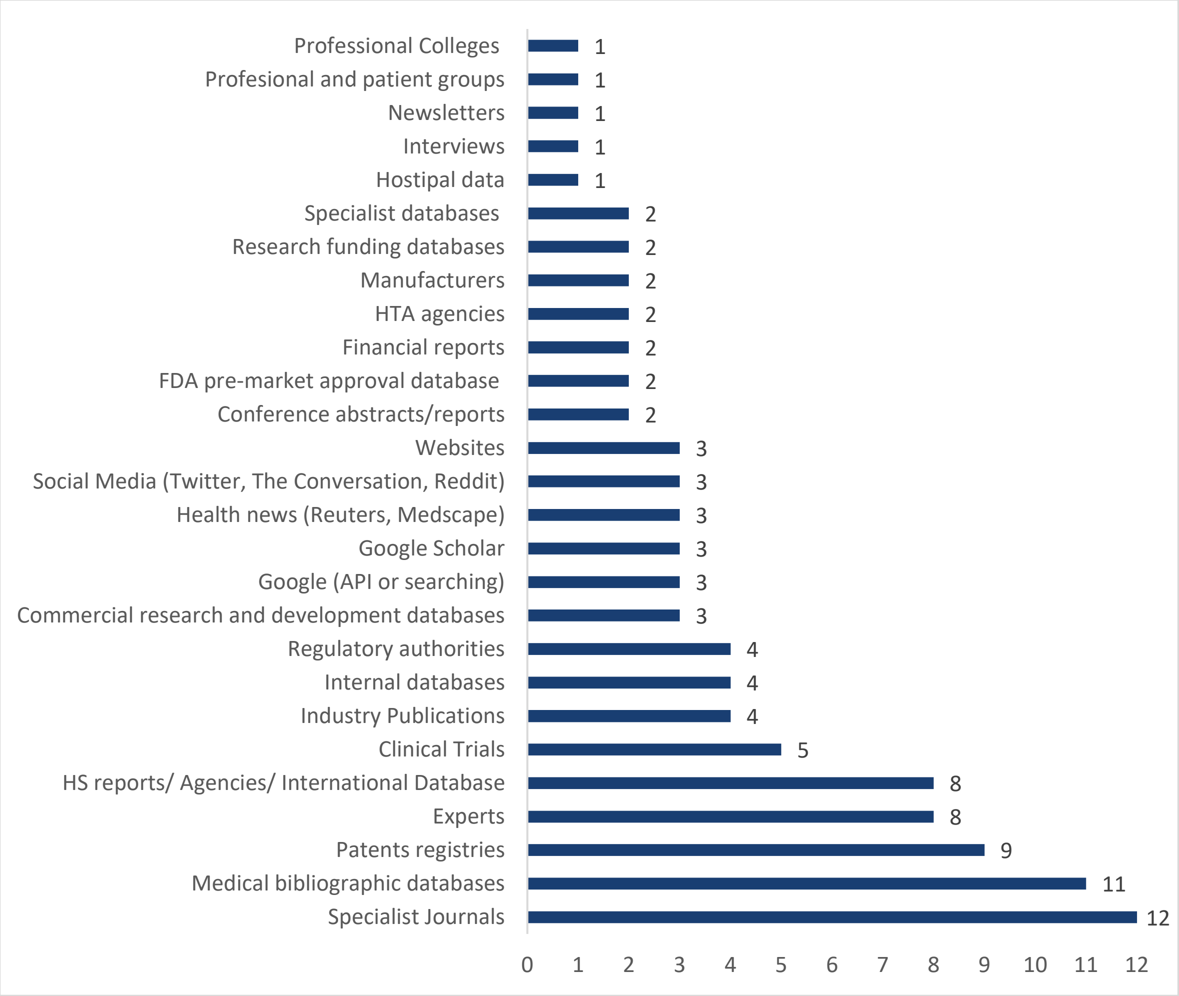

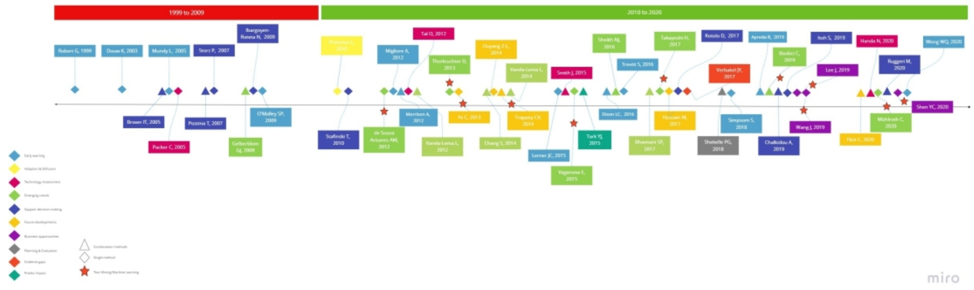

Figure 4. Development of methods over a publication timeline

885x316mm (72 x 72 DPI)

Appendix A

Table A.1. PRISMA-S Checklist

Section/topic	#	Checklist item	Location(s) Reported
**INFORMATION SOURCES AND METHODS**			
Database name	1	Name each individual database searched, stating the platform for each.	Pg. 4-5 and Appendix B
Multi-database searching	2	If databases were searched simultaneously on a single platform, state the name of the platform, listing all of the databases searched.	N/A
Study registries	3	List any study registries searched.	N/A
Online resources and browsing	4	Describe any online or print source purposefully searched or browsed (e.g., tables of contents, print conference proceedings, web sites), and how this was done.	Pg. 4-5 and Appendix B
Citation searching	5	Indicate whether cited references or citing references were examined, and describe any methods used for locating cited/citing references (e.g., browsing reference lists, using a citation index, setting up email alerts for references citing included studies).	Pg. 4-5
Contacts	6	Indicate whether additional studies or data were sought by contacting authors, experts, manufacturers, or others.	N/A
Other methods	7	Describe any additional information sources or search methods used.	Pg. 4-5 and Appendix B
**SEARCH STRATEGIES**			
Full search strategies	8	Include the search strategies for each database and information source, copied and pasted exactly as run.	Appendix B
Limits and restrictions	9	Specify that no limits were used, or describe any limits or restrictions applied to a search (e.g., date or time period, language, study design) and provide justification for their use.	Pg. 4-5
Search filters	10	Indicate whether published search filters were used (as originally designed or modified), and if so, cite the filter(s) used.	N/A

For peer review only

Prior work	11	Indicate when search strategies from other literature reviews were adapted or reused for a substantive part or all of the search, citing the previous review(s).	N/A
Updates	12	Report the methods used to update the search(es) (e.g., rerunning searches, email alerts).	N/A
Dates of searches	13	For each search strategy, provide the date when the last search occurred.	Appendix B
**PEER REVIEW**			
Peer review	14	Describe any search peer review process.	Pg. 4-5
**MANAGING RECORDS**			
Total Records	15	Document the total number of records identified from each database and other information sources.	Appendix B
Deduplication	16	Describe the processes and any software used to deduplicate records from multiple database searches and other information sources.	Pgs. 4-5

PRISMA-S: An Extension to the PRISMA Statement for Reporting Literature Searches in Systematic Reviews
Rethlefsen ML, Kirtley S, Waffenschmidt S, Ayala AP, Moher D, Page MJ, Koffel JB, PRISMA-S Group.
Last updated February 27, 2020.

For peer review only

Appendix B

Figure B.1. MEDLINE search strategy

1	Forecasting/
2	(horizon scan or horizon scanning).ti,ab,kf,tw.
3	(environmental scan or environmental scanning).ti,ab,kf,tw.
4	(foresight or forecasting).ti,ab,kf,tw.
5	("Early awareness" and "alert systems").ti,ab,kf,tw.
6	(readiness adj3 preparedness).ti,ab,kf,tw.
7	(monitor* adj6 ("health technolog*" or "medical technolog*")).ti,ab,kf,tw.
8	("road map" or roadmap).ti,ab,kf,tw.
9	patent analys*s.ti,ab,kf,tw.
10	bibliometric analys*s.ti,ab,kf,tw.
11	trend* analys*s.ti,ab,kf,tw.
12	backcasting.ti,ab,kf,tw.
13	delphi.ti,ab,kf,tw.
14	*delphi technique/
15	or/1-14
16	biomedical technology/
17	exp technology assessment, biomedical/
18	((new or innovat* or emerg* or early) adj6 ("health technolog*" or "medical technolog*")).ti,ab.
19	or/16-18
20	15 and 19

Databases searched

Medline (OVID)

Database: Ovid MEDLINE(R) and Epub Ahead of Print, In-Process, In-Data-Review & Other Non-Indexed Citations, Daily and Versions(R) <1946 to March 25, 2021>

Date: 26/03/2021

Search results: 1003

Search Strategy:

----

1 Forecasting/ (87084)

2 (horizon scan or horizon scanning).ti,ab,kf,tw. (247)

3 (environmental scan or environmental scanning).ti,ab,kf,tw. (1780)

4 (foresight or forecasting).ti,ab,kf,tw. (10290)

5 ("Early awareness" and "alert systems").ti,ab,kf,tw. (9)

6 (readiness adj3 preparedness).ti,ab,kf,tw. (203)

7 (monitor* adj6 ("health technolog*" or "medical technolog*")).ti,ab,kf,tw. (93)

8 ("road map" or roadmap).ti,ab,kf,tw. (7122)

For peer review only

9 patent analys*s.ti,ab,kf,tw. (75)

10 bibliometric analys*s.ti,ab,kf,tw. (2302)

11 trend* analys*s.ti,ab,kf,tw. (5160)

12 backcasting.ti,ab,kf,tw. (29)

13 delphi.ti,ab,kf,tw. (12256)

14 *delphi technique/ (1233)

15 or/1-14 (123740)

16 biomedical technology/ (6719)

17 exp technology assessment, biomedical/ (11322)

18 ((new or innovat* or emerg* or early) adj6 ("health technolog*" or "medical technolog*")).ti,ab. (1640)

19 or/16-18 (18729)

20 15 and 19 (1003)

2. Embase

Database: Embase <1974 to 2021 March 25>

Date: 26/03/2021

Search results: 1295

Search Strategy:

----

1 forecasting/ (46958)

2 (horizon scan or horizon scanning).ti,ab,kw. (341)

3 (environmental scan or environmental scanning).ti,ab,kw. (2175)

4 (foresight or forecasting).ti,ab,kw. (12472)

5 (readiness adj3 preparedness).ti,ab,kw. (218)

6 (monitor* adj6 ("health technolog*" or "medical technolog*")).ti,ab,kw. (142)

7 ("road map" or roadmap).ti,ab,kw. (8918)

8 patent analys*s.ti,ab,kw. (181)

9 bibliometric analys*s.ti,ab,kw. (2705)

10 trend* analys*s.ti,ab,kw. (7382)

11 backcasting.ti,ab,kw. (31)

12 delphi.ti,ab,kw. (16900)

13 *delphi study/ (2074)

For peer review only

14 *bibliometrics/ (3371)

15 *trend study/ (865)

16 or/1-15 (95349)

17 medical technology/ (34204)

18 biomedical technology assessment/ (15008)

19 ((new or innovat* or emerg* or early) adj6 ("health technolog*" or "medical technolog*")).ti,ab. (2248)

20 or/17-19 (49615)

21 16 and 20 (1295)

3. IEEE Xplore

Database URL: https://ieeexplore-ieee-org.libproxy.ncl.ac.uk/search

Search Date: 05032021

Search strategy: database does not allow to combine search strings or save searches. Searched string by string and assessed relevance of results on screen by title and abstract. Those results judged relevant as first screening have been downloaded for further assessment.

No time limit, No language limits

Showing 1-4 of 4 for ("Document Title":"horizon scan" OR "Abstract":"horizon scan" OR "Index Terms":"horizon scan") – search retrieved no relevant results

Showing 1-25 of 437 for ("Publication Title":"foresight" OR "Abstract":"foresight" OR "Index Terms":"foresight") – all 437 results downloaded for further assessment

Conferences (352)

Journals (50)

Magazines (21)

Early Access Articles (12)

Books (1)

Courses (1)

Showing 1-25 of 71 for (("Document Title":"forecast" OR "Abstract":"forecast" OR "Index Terms":"forecast") AND "Document Title":"health") – all 71 results downloaded for further assessment

Conferences (59)

Journals (11)

Magazines (1)

Showing 1-19 of 19 for (("Document Title":"roadmap" OR "Abstract":"roadmap" OR "Index Terms":"roadmap") AND "Document Title":"health") – search retrieved no relevant results

For peer review only

Showing 1-17 of 17 for (("Document Title":"patent" OR "Document Title":"bibliometric" OR "Document Title":"trend" OR "Document Title":"backcasting" OR "Document Title":"delphi") AND "Document Title":"health") – 1 reference downloaded for further assessment/all references downloaded for further assessment.

4. Compendex – Engineering Village (Elsevier)

Database URL: https://www.engineeringvillage.com/search/quick.url

Search date: 26/03/2020

Search strategy:

((((((environmental scanning NEAR/6 health) OR (horizon scanning NEAR/6 health) OR (Forecasting NEAR/6 health) OR (Foresight NEAR/06 health) OR (Roadmap NEAR/06 health) OR (patent analys*s NEAR/06 health) OR (bibliometric analys*s NEAR/06 health) OR (trend analys*s NEAR/06 health) OR (backcasting NEAR/06 health) OR (delphi NEAR/06 health)) WN KY)) NOT ({cp} WN DT)) NOT (({ch} OR {bk} OR {er}) WN DT))

Removed conference proceedings, books and book chapters:

Retrieved results: 3,465

5. Scopus

Advanced search interface

Database URL: https://www.scopus.com/search/form.uri?display=advanced

Search date: 09/03/2021

Retrieved results: 2,396

Search strategy:

(TITLE-ABS-KEY ("horizon scanning") OR TITLE-ABS-KEY (forecasting) OR TITLE-ABS-KEY ("road map" OR roadmap) OR TITLE-ABS-KEY (patent AND anlys*s) OR TITLE-ABS-KEY (bibliometric AND analys*s) OR TITLE-ABS-KEY (trend AND analys*s) OR TITLE-ABS-KEY (backcasting) OR TITLE-ABS-KEY (delphi)) AND (TITLE-ABS-KEY ("medical technology") OR TITLE-ABS-KEY ("health technology"))

6. Web of Science CORE Collection

Advanced search interface

Database URL: https://apps.webofknowledge.com/WOS_AdvancedSearch_input.do?product=WOS&search_mode=AdvancedSearch&SID=D5cLI4VsqgVb6Yc6hBB&locale=en_US

Date searched 09/03/2021

Retrieved results: 214 all downloaded for further assessment

Search strategy:

Set	Results	Save History / Create AlertOpen Saved History
# 12	214	#11 AND #8<br>*Indexes=SCI-EXPANDED, SSCI, A&HCI, CPCI-S, CPCI-SSH, ESCI Timespan=All years*
# 11	11,875	#10 OR #9<br>*Indexes=SCI-EXPANDED, SSCI, A&HCI, CPCI-S, CPCI-SSH, ESCI Timespan=All years*
# 10	7,049	TI=("health technology" OR "health technologies") OR AB=("health technology" OR "health technologies")<br>*Indexes=SCI-EXPANDED, SSCI, A&HCI, CPCI-S, CPCI-SSH, ESCI Timespan=All years*
# 9	4,942	TI=("medical technology" OR "medical technologies") OR AB=("medical technolo gy" OR "medical technologies")<br>*Indexes=SCI-EXPANDED, SSCI, A&HCI, CPCI-S, CPCI-SSH, ESCI Timespan=All years*
# 8	236,408	#7 OR #6 OR #5 OR #4 OR #3 OR #2 OR #1<br>*Indexes=SCI-EXPANDED, SSCI, A&HCI, CPCI-S, CPCI-SSH, ESCI Timespan=All years*
# 7	19,579	TI=(delphi) OR AB=(delphi)<br>*Indexes=SCI-EXPANDED, SSCI, A&HCI, CPCI-S, CPCI-SSH, ESCI Timespan=All years*
# 6	345	TI=(backcasting) OR AB=(backcasting)<br>*Indexes=SCI-EXPANDED, SSCI, A&HCI, CPCI-S, CPCI-SSH, ESCI Timespan=All years*
# 5	10,036	TI=("trend analysis" OR "trend analyses") OR AB=("trend analysis" OR "trend an alyses")<br>*Indexes=SCI-EXPANDED, SSCI, A&HCI, CPCI-S, CPCI-SSH, ESCI Timespan=All years*
# 4	5,608	TI=("bibliometric analysis" OR "bibliometric analyses") OR AB=("bibliometric ana lysis" OR "bibliometric analyses")<br>*Indexes=SCI-EXPANDED, SSCI, A&HCI, CPCI-S, CPCI-SSH, ESCI Timespan=All years*
# 3	749	TI=("patent analysis" OR "patent analyses") OR AB=("patent analysis" OR "paten t analyses")<br>*Indexes=SCI-EXPANDED, SSCI, A&HCI, CPCI-S, CPCI-SSH, ESCI Timespan=All years*
# 2	21,200	TI=("road map" OR roadmap) OR AB=("road map" OR roadmap)<br>*Indexes=SCI-EXPANDED, SSCI, A&HCI, CPCI-S, CPCI-SSH, ESCI Timespan=All years*
# 1	180,250	TI=(horizon scanning OR forecasting) OR AB=(horizon scanning OR forecasting)

For peer review only

Indexes=SCI-EXPANDED, SSCI, A&HCI, CPCI-S, CPCI-SSH, ESCI Timespan=All years

7. International HTA Database

URL: https://database.inahta.org/search/advanced

Date searched 04/02/2022

Retrieved results: 334

Search strategy:

(forecast*)[Title] OR (horizon scan*)[Title] OR (environmental scan*)[Title] OR (foresight)[Title] OR ("early awareness" AND "alert systems")[Title] OR (roadmap* OR "road map*")[Title] OR ("bibliometric analys*")[Title] OR (backcasting)[Title] OR ("trend analys*")[Title] OR (delphi) AND ((new OR innovative OR early OR emerging))

Retrieved 334. Sifted on screen. 35 results downloaded for further assessment. Included in lit review: 0

Grey literature searches:

Platform: Google Chrome

Search strategy:

horizon scanning filetype:pdf

Date: 26/02/2021

Results: limited to 2020 onwards, checked the first 5 pages of results.

Included for further analysis:

https://www.pcori.org/sites/default/files/PCORI-Health-Care-COVID-19-Horizon-Scanning-System-Supplement-High-Impact-Report-September-2020.pdf

https://ec.europa.eu/info/sites/info/files/srip/2020/rec-19-003_srip_chap-15.pdf

https://www.mdpi.com/2071-1050/12/23/10194/pdf

https://htai.org/wp-content/uploads/2020/03/HTAi_APF-2019-Background-Paper.pdf

Database: CORE

Date searched 26/02/2021

Search strategy: “horizon scanning”

Retrieved results: 5,582

For peer review only

Hand searched the first 5 results pages.

Included for further assessment: 2

Blackburn, Steven, Cudd, Peter and Hawley, Mark (2010) A horizon scanning system for identifying new telehealth innovations. Global Telemedicine and eHealth Updates: Knowledge Resources, 3. pp. 326-330. ISSN 1998-5509 http://eprints.whiterose.ac.uk/11144/

Enhancing Horizon Scanning by utilizing pre-developed scenarios: analysis of current practice and specification of a process improvement to aid the identification of important 'weak signals'. https://www.sciencedirect.com/science/article/pii/S0040162517300707

Database: International HTA Database

Date searched: 25/01/2022

Search strategy: "horizon scanning" in title

Retrieved results: 310

Sifted on screen, none included

For peer review only

Appendix C

Figure C.1. PRISMA flow chart

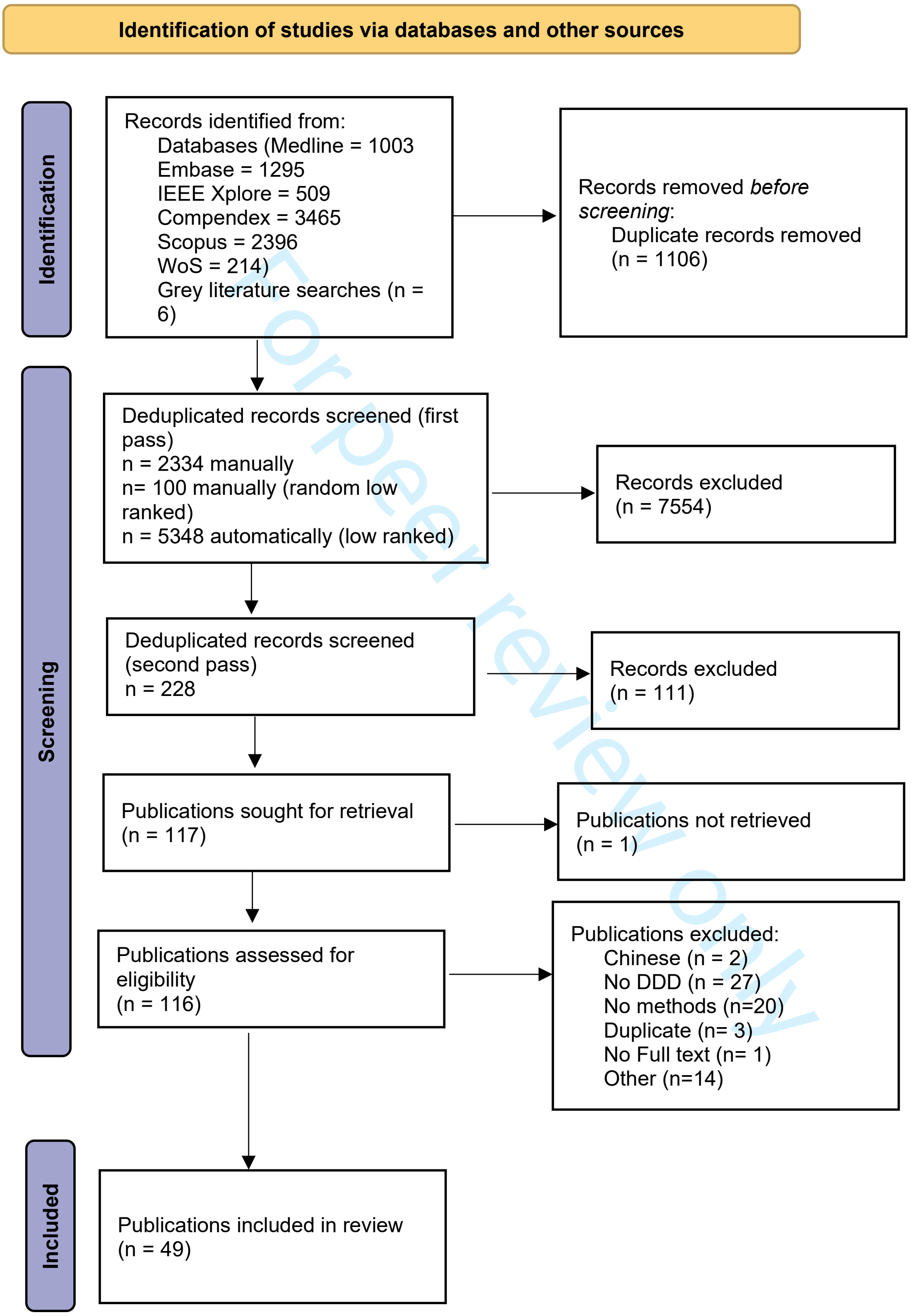

From: Page MJ, McKenzie JE, Bossuyt PM, Boutron I, Hoffmann TC, Mulrow CD, et al. The PRISMA 2020 statement: an updated guideline for reporting systematic reviews. BMJ 2021;372:n71. doi: 10.1136/bmj.n71

For peer review only

Appendix D. Table 1: Included studies characteristics: methods, sources, technology types and time horizon

Authors	Organization/ Geography	Study Type	Main purpose	Methods used	Sources	Technology type	Horizon
**Apreda R, 2019**	University of Pisa and Research Institute for the Evaluation of Public Policies (Italy)	Methods research paper	Early warning	Combination	Experts Stakeholders	Medical devices	Prospective<br>5-year horizon<br>Scan undertaken in 2010-2011
				Expert panel; Desk research on patents and literature searches for scientific publications; Functional analysis; Consultation of results with external stakeholders	Published and Unpublished literature		
					Patents		
**Baskici C, 2019**	Baskent University (Turkey)	Conference paper	Identify emerging trends	Combination	Experts	All	Retrospective<br>2013-2015 (retrospective search)
				Text mining; Web scrapping; Patent analysis; Expert consultation	Patents<br>Clinical trials		
					Published literature		

Authors	Organization/ Geography	Study Type	Main purpose	Methods used	Sources	Technology type	Horizon
**Bhavnani SP, 2017**	American College of Cardiology (USA)	Policy	Identify emerging trends	Combination	Experts	All (digital health, big data, and precision health)	Not specified
				Horizon scanning; Literature searches; network for gathering and sharing information	Published/ Unpublished literature		
**Brown IT, 2005**	Department of Electrical & Computer Systems Engineering, Monash University (Australia)	Published educational note	Support decision making	Combination	Stakeholders; Experts	Medical devices for remote patient monitoring	Not specified
				Systematic literature review; Stakeholders' Survey; Experts consultation	Published literature		
					Approved commercial devices database		

For peer review only

Authors	Organization/ Geography	Study Type	Main purpose	Methods used	Sources	Technology type	Horizon
**Chalkidou A, 2019**	University College London<br>(London, UK)	Conference abstract	Support decision making	Combination	Experts	MedTech: a hybrid theatre incorporating a biplane, intraoperative MRI (iMRI), multi-detector computed tomography (CT) scanners, and an EOS imaging system	Not specified
				Literature search; Experts	Published literature		
					Hospital data		
**Chang SWC, 2014**	National Tsing Hua University and National Chiao Tung University<br>(Taiwan)	Conference paper	Identify emerging trends	Combination	Published literature	Dental implants	Retrospective<br>1998 - 2002
				Literature search; patent search	Patents		
**Dixon LC, 2016**	NIHR Horizon Scanning		Early warning	Combination	Clinical experts and patients	All (Devices, Diagnostics,	Prospective and Retrospective

For peer review only

Authors	Organization/<br>Geography	Study Type	Main purpose	Methods used	Sources	Technology type	Horizon
	Research & Intelligence Centre (HSRIC)<br>(Birmingham, UK)	Horizon Scanning report		Literature search; Clinical experts; patients	Published/Unpu blished literature	Digital and "Other")	Technologies licensed within the last 2 years or in development planned to launch within the next 18 months
**Douw K, 2003**	Collaboration between Public Health, HTA and HS organisations<br>(Denmark & UK)	Methods research paper	Early warning	Single<br>Searching the internet	Internet	All	Not specified
**Flick C, 2020**	Collaboration across UK and non-UK universities and research institutes<br>(EU)	Methods research paper	Identify future developments	Single<br>Horizon scanning	Published/Unpu blished literature<br>Social media	All	Retrospective<br>2014-2015 (scan undertaken in 2015) and<br>2016-2017 (scan undertaken in 2017)

For peer review only

Authors	Organization/ Geography	Study Type	Main purpose	Methods used	Sources	Technology type	Horizon
**Gelderblom GJ, 2009**	European Commission Directorate General Information Society and Media<br>(EU)	Conference paper	Identify emerging trends	Combination	Published (potentially including unpublished) literature	Robotics	Prospective<br>2009 to 2025
				Literature search; Expert and stakeholders survey and interviews; workshop for drafting the roadmap	Experts		
**Handa N, 2020**	Pharmaceuticals and Medical Devices Agency of Japan (PMDA)<br>(Japan)	Horizon Scanning report	Technology assessment	Combination	Experts;	Artificial organs	Not specified
				Horizon scanning and expert consultation			
**Hussain M, 2017**	Aston University<br>(Birmingham, UK)	Methods research paper	Identify future developments	Single	Stakeholders	Radio Frequency IDentification (RFID) technology	Not specified
				Scenario-Driven roadmapping			

For peer review only

Authors	Organization/ Geography	Study Type	Main purpose	Methods used	Sources	Technology type	Horizon
**Ibargoyen-Roteta N, 2009**	Horizon scanning organisation linked to EuroScan Network<br><br>(Spain)	Horizon Scanning methods paper	Support decision making	Single<br><br>Surveying other HS organizations	Stakeholders (other HS agencies)	Potentially Obsolete Technologies (POT)	Not applicable
**Itoh S, 2019**	Japan's Ministry of Health, Labour and Welfare and Ministry of Economy, Trade and Industry (METI) (MHLW)<br><br>(Japan)	Methods research paper	Support decision making	Single<br><br>Survey with experts, clinicians and companies	Stakeholders	Medical devices	Retrospective<br><br>2004 to 2014
**Lee J, 2019**	Researchers<br><br>(Korea)	Methods research paper	Identify business opportunities	Single<br><br>Twitter data related to health technology	Twitter	Medical health technologies including devices and digital health	Retrospective<br><br>January 2010 to October 2016
**Lerner JC, 2019**	ECRI Institute<br><br>(USA)	Horizon Scanning	Early warning	Single	Forecasts published by ECRI Institute	All (innovations that could	Retrospective<br><br>2007-2010

Authors	Organization/ Geography	Study Type	Main purpose	Methods used	Sources	Technology type	Horizon
		case study		Content analysis	used as case studies	create significant shifts in modes of care delivery, patient health outcomes, health care costs, technology utilization, and infrastructure needed to deliver care)	
**Migliore A, 2012**	Agenzia Nazionale per i Servizi Sanitari Regionali through its project COTE (Centro Osservazione Tecnologie sanitarie Emergenti) (Italy)	Horizon Scanning methods paper	Early warning	Single<br>Stakeholder engagement	Stakeholders	Medical devices	Not specified

For peer review only

Authors	Organization/ Geography	Study Type	Main purpose	Methods used	Sources	Technology type	Horizon
**Morrison A, 2012**	Canadian Agency for Drugs and Technologies in Health (CADTH)<br>(Canada)	Horizon Scanning methods paper	Early warning	Combination	Stakeholders	Drug and non-drug medical health technologies including devices and diagnostics	Prospective<br>2 to 3 years to licence or diffuse
				Horizon scanning and environmental scans; Stakeholder engagement	Published/<br>Unpublished literature		
**Mühlroth C, 2020**	Friedrich-Alexander-Universität Erlangen-Nürnberg<br>(Germany)	Methods research paper	Identify emerging trends	Single	Not reported	All	Not specified
				Strategic foresight			
**Mundy L, 2005**	Australia and New Zealand Horizon Scanning Network (ANZHSN)<br>(Australia & New Zealand)	Horizon Scanning methods paper	Early warning	Single	Published (potentially including unpublished) literature	Medical devices, diagnostics and procedures	Prospective<br>3 years to licence
				Horizon Scanning	News		
					Regulatory information		

For peer review only

Authors	Organization/ Geography	Study Type	Main purpose	Methods used	Sources	Technology type	Horizon
**O'Malley S P, 2009**	Australia and New Zealand Horizon Scanning Network (ANZHSN)<br>(Australia & New Zealand)	Horizon Scanning methods paper	Early warning	Single	Stakeholders (other HS agencies)<br>Experts	Medical devices and diagnostics	Prospective<br>> 10 years
				Horizon Scanning	Published/ Unpublished literature		
					News		
					Regulatory information		
**Zhao-Lian Ouyang Yu-Bo Fan, 2014**	Beijing University of Aeronautics & Astronautics and Chinese Academy of Medical Sciences<br>(China)	Methods research paper	Identify future developments	Single	Patents	Medical radiation diagnosis devices	Retrospective<br>2003 to 2012
				Patent bibliometric analysis			
**Packer C, 2005**	NIHR Horizon Scanning Research & Intelligence Centre (HSRIC)	Horizon scanning methods paper	Technology assessment	Single	Experts; Industry; Stakeholders (other HS agencies)	All	Not specified

For peer review only

Authors	Organization/<br>Geography	Study Type	Main purpose	Methods used	Sources	Technology type	Horizon
	(Birmingham, UK)			Horizon Scanning	Published/Unpublished literature		
**Postma T, 2017**	University of Groningen and Dutch Cancer Society and Dutch Cancer Society<br>(The Netherlands)	Methods research paper	Support decision making	Combination<br>Group expert opinion forecasting using the Delphi-technique, and a market-oriented capacity analysis using projective scenarios	Experts	All	Not specified
**Pretorius L, 2017**	University of Pretoria<br>(South Africa)	Horizon Scanning case study	Adoption and diffusion	Single<br>Trend analysis	Bibliometric data from Google Scholar and SciDirect	Medical devices and diagnostic	Retrospective<br>10-15 years
**Robert G, 1999**	NHS Research and Development HTA programme	Horizon scanning	Early warning	Single	Experts	All	Not specified

For peer review only

Authors	Organization/ Geography	Study Type	Main purpose	Methods used	Sources	Technology type	Horizon
	(UK)	methods paper		Delphi technique			
**Rotolo D, 2015**	University of Sussex; University of Valencia; University of Amsterdam; University of Sussex<br>(EU)	Methods research paper	Support decision making	Single	Published literature	All	Retrospective<br>1982 to 2011
				Scientometric mapping approach: overlay mapping	Patents		
**Ruggeri M, 2020**	National Centre for HTA, Istituto Superiore di Sanità<br>(Italy)	Horizon scanning case study	Support decision making	Combination	Experts; Stakeholders	All (devices and digital apps)	Not specified
				Horizon scanning and experts panel			
**Sheikh NS, 2016**	Department of Technology and Society, University of Korea<br>(Korea)	Conferen ce paper	Identify emerging trends	Combination	Bibliometric data	Devices (biosensor technologies based on human saliva, breath or blood)	Not specified
				Bibliometric and trend analyses	Patents		

For peer review only

Authors	Organization/ Geography	Study Type	Main purpose	Methods used	Sources	Technology type	Horizon
**Shekelle PG, 2018**	Researchers at University of Southern California Institutional Report Board and the RAND Corporation Human Subject Protection Committee (USA)	Methods research paper	Planning & Evaluation	Combination	Experts	Medical devices and diagnostics	Retrospective analysis of a prospective prediction 20 years
				Systematic literature searches and multidisciplinary experts	Published (potentially including unpublished) literature		
**Shen Y C, 2020**	Research department of Biobusiness Management (Taiwan)	Methods research paper	Identify business opportunities	Single	Published literature	Medical devices	Retrospective from database inception to 2015
				Literature search and patent search	Patents		
**Simpson S, 2018**	NIHR Horizon Scanning Research &	Methods research paper	Early warning	Combination	Patients and the Public	All	Not specified

For peer review only

Authors	Organization/ Geography	Study Type	Main purpose	Methods used	Sources	Technology type	Horizon
	Intelligence Centre (HSRIC) (Birmingham, UK)			Horizon scanning and PPIE			
**Smith J, 2015**	NIHR Horizon Scanning Research & Intelligence Centre (HSRIC) (Birmingham, UK)	Horizon scanning report	Technology assessment	Combination<br>Horizon scanning, consultation with clinical experts and PPIE	Patients and the Public<br>Experts	All	Not specified
**de Souza Antunes AM, 2012**	Instituto Nacional de Propriedade Industrial (Brazil)	Methods research paper	Identify emerging trends	Single<br>Patent trend analysis	Patents	Nanotechnology applied to the screening, diagnosis, and treatment of disease	Retrospective<br>2000 to 2010
**Stafinski T, 2020**	Canadian Agency for Drugs and Technologies in Health (CADTH) (Canada)	Methods research paper	Support decision making	Single<br>Expert consultation	Experts	All	Prospective<br>5 years ahead

For peer review only

Authors	Organization/ Geography	Study Type	Main purpose	Methods used	Sources	Technology type	Horizon
**Storz P, 2007**	German HTA in collaboration with the Federal Association of the Local Health Insurance Funds (AOK Bundesverband)<br>(Germany)	Horizon scanning report	Support decision making	Combination<br>Horizon Scanning; Literature searching	Published/ Unpublished literature<br>Internet<br>ZIM (private) database	Genetic testing	Not specified
**Takayoshi H, 2017**	VALUENEX Japan Inc<br>(Japan)	Methods research paper	Identify emerging trends	Single<br>Panoramic view analytics	Patents	Artificial intelligence applied to health and wellbeing	Retrospective<br>Published between 2001 and 2016
**Tal O, 2012**	Israeli Center for Technology Assessment in Health Care (ICTAHC)<br>(Israel)	Horizon Scanning methods paper	Technology assessment	Single<br>Horizon scanning using 3 time horizons	Experts<br>Industry<br>Published/	Medical devices	Prospective<br>Three horizons: short term 1 year; medium term and long-term

For peer review only

Authors	Organization/ Geography	Study Type	Main purpose	Methods used	Sources	Technology type	Horizon
					Unpublished literature<br>Internet		
					Clinical trials		
**Tark YJ, 2015**	Horizon Scanning Service of Innovative Global Health Technology (H-SIGHT)<br>(South Korea)	Horizon scanning report	Predict impact in healthcare services	Combination	Published literature	Medical device (polyurethane scaffolds)	Retrospective evidence search<br>Prospective prediction of impact (1 to 5 years) from launch
				Horizon scanning; Literature report; Clinical trials search	Clinical trials		
**Thorleuchter D, 2013**	Fraunhofer Institute for Technological Trend Analysis INT<br>(Germany)	Methods research paper	Identify emerging trends	Single	Internet	All	Not specified
				Web mining and latent semantic indexing			
**Trappey CV, 2014**	National Tsing Hua University and National		Identify future developments	Combination	Patents	Dental implant connectors	Retrospective (published

For peer review only

Authors	Organization/ Geography	Study Type	Main purpose	Methods used	Sources	Technology type	Horizon
	Chiao Tung University (Taiwan)	Methods research paper		Text mining and literature searches	Published clinical trials		between 1992 and 2012)
**Trevitt S, 2016**	NIHR Horizon Scanning Research & Intelligence Centre (HSRIC) (Birmingham, UK)	Horizon Scanning report	Early warning	Single	Published/ Unpublished literature	Wearable medical devices second and third generation Artificial Pancreas Device systems	Not specified
				Horizon scanning	Clinical trials		
					Internet; Internal databases; Third party databases; Manufacturer's websites		
**Vaganova EV, 2015**	National Research Tomsk State University (Russia)	Conference paper	Identify emerging trends	Single	Published literature	Medical devices and digital interventions (e-health and m-health, telemedicine and personalised smart devices)	Not specified
				Forecasting	Patents		

For peer review only

Authors	Organization/ Geography	Study Type	Main purpose	Methods used	Sources	Technology type	Horizon
**Varela-Lema L, 2012**	Galician Agency for Health Technology Assessment (avalia-t) (Spain)	Methods research paper	Identify emerging trends	Combination	Experts	Medical devices, procedures and diagnostics	Retrospective (4 to 5 years)
				Horizon scanning and expert consultation	Published/ Unpublished literature		
**Varela-Lema L, 2014**	Galician Agency for Health Technology Assessment (avalia-t) (Spain)	Methods research paper	Identify emerging trends	Single	Published literature	Medical devices, procedures and diagnostics	Prospective (1 to 2 years from availability)
				Bibliographic database search			
**Verbakel Jan Y, 2017**	National Institute for Health Research (NIHR) Diagnostic Evidence Co-operative Oxford at Oxford Health Foundation Trust (Oxford, UK)	Horizon scanning report	Identify evidence gaps	Single	Horizon scanning reports	Diagnostic tests	Not specified
				Content analysis			
				Single	Patents	Telehealth	

For peer review only

Authors	Organization/ Geography	Study Type	Main purpose	Methods used	Sources	Technology type	Horizon
**Wang J, 2019**	National Chung Hsing University (Taiwan)	Methods research paper	Identify business opportunities	Patent trend analysis			Retrospective (1991 to 2015)
**Wong Q W, 2020**	Singapore HS Organization (Singapore)	Methods research paper	Early warning	Single<br>Horizon Scanning	Commercial developer websites<br>Regulatory authorities (FDA and EMA)<br>Medical news media; Scientific journals; Conference proceedings<br>Reports from other horizon scanning organizations (CADTH, NIHR IO)<br>Trial registries	All (disruptive medicines, devices, digital and diagnostics)	Prospective (3 years to regulatory approval for drugs, different time horizon for DDD)
**Ye C, 2013**				Single			Not specified

For peer review only

Authors	Organization/ Geography	Study Type	Main purpose	Methods used	Sources	Technology type	Horizon
	Beijing City University (China)	Conference paper	Identify future developments	Technology roadmap	Nanotechnology Roadmap of U.S. NASA	All (not specified)	

For peer review only
